# Machine learning prediction of methane, nitrogen, and natural gas mixture viscosities under normal and harsh conditions

**DOI:** 10.1038/s41598-024-64752-8

**Published:** 2024-07-02

**Authors:** Sayed Gomaa, Mohamed Abdalla, Khalaf G. Salem, Karim Nasr, Ramadan Emara, Qingsheng Wang, A. N. El-hoshoudy

**Affiliations:** 1 Mining and Petroleum Engineering Department, Faculty of Engineering, Al-Azhar University, Cairo, Egypt; 2 Department of Petroleum Engineering, Faculty of Engineering and Technology, Future University in Egypt (FUE), Cairo, 11835 Egypt; 3 Department of Multidisciplinary Engineering, Texas A&M University, College Station, TX USA; 4 Artie McFerrin Department of Chemical Engineering, Texas A&M University, College Station, TX USA; 5 Department of Reservoir Engineering, South Valley Egyptian Petroleum Holding Company (GANOPE), Cairo, Egypt; 6 Petroleum Engineering and Gas Technology Department, Faculty of Energy and Environmental Engineering, British University in Egypt (BUE), El Shorouk City, Cairo Egypt; 7 PVT Lab, Production Department, Egyptian Petroleum Research Institute, Cairo, 11727 Egypt; 8 PVT Service Center, Egyptian Petroleum Research Institute, Cairo, 11727 Egypt

**Keywords:** Gas viscosity, Machine learning, Artificial neural network (ANN), Regression models, Pressure–volume-temperature (PVT) tests, Sensitivity analysis, Chemical engineering, Natural gas, Crude oil

## Abstract

The accurate estimation of gas viscosity remains a pivotal concern for petroleum engineers, exerting substantial influence on the modeling efficacy of natural gas operations. Due to their time-consuming and costly nature, experimental measurements of gas viscosity are challenging. Data-based machine learning (ML) techniques afford a resourceful and less exhausting substitution, aiding research and industry at gas modeling that is incredible to reach in the laboratory. Statistical approaches were used to analyze the experimental data before applying machine learning. Seven machine learning techniques specifically Linear Regression, random forest (RF), decision trees, gradient boosting, K-nearest neighbors, Nu support vector regression (NuSVR), and artificial neural network (ANN) were applied for the prediction of methane (CH_4_), nitrogen (N_2_), and natural gas mixture viscosities. More than 4304 datasets from real experimental data utilizing pressure, temperature, and gas density were employed for developing ML models. Furthermore, three novel correlations have developed for the viscosity of CH_4_, N_2_, and composite gas using ANN. Results revealed that models and anticipated correlations predicted methane, nitrogen, and natural gas mixture viscosities with high precision. Results designated that the ANN, RF, and gradient Boosting models have performed better with a coefficient of determination (R^2^) of 0.99 for testing data sets of methane, nitrogen, and natural gas mixture viscosities. However, linear regression and NuSVR have performed poorly with a coefficient of determination (R^2^) of 0.07 and − 0.01 respectively for testing data sets of nitrogen viscosity. Such machine learning models offer the industry and research a cost-effective and fast tool for accurately approximating the viscosities of methane, nitrogen, and gas mixture under normal and harsh conditions.

## Introduction

Natural gas is one of the major naturally occurring and efficient energy sources, consisting of a complex mixture of light hydrocarbon compounds (C_1_–C_11_, where methane > 90% v/v) and a minor inorganics (non-hydrocarbon) compound such as (CO_2_, N_2_, H_2_S, and He) as well as a few heavy metals^1–3^. It is considered a subcategory of petroleum fluids which is a versatile, abundant, and clean energy source for both domestic and industrial processes owing to smaller greenhouse gas emissions and air pollution^4^. Global energy demand increases annually which led to the utilization of virgin HPHT reservoirs^5^. A precise estimation of the natural gas thermophysical properties and other hydrocarbon components including gas viscosity is crucial for gas engineering problems and physicochemical calculations^2^. Viscosity denotes the fluid’s resistance to the shearing action caused by molecular diffusion, which transfers momentum between flow layers^6^. It is an efficient theoretical and practical property for gas processes optimization including phase behavior analysis, the study of single-phase equations, reservoir simulation and characterization, estimation of pressure gradient for gas wells, fluid-flow dynamics in transportation pipelines, optimal exploitation, multiphase flow in porous media through gas and oil reservoirs, enhanced oil recovery^7–9^, gas reserves estimation, and molecular interactions including transport of momentum occur in a fluid motion and tubing^1,6,10^. For HPHT gas reservoirs, low gas viscosity errors adversely affect the inflow performance relationship (IPR) curves and, as a result, the reserves estimates^11^. Gases exhibit non-traditional reactions at high and low pressures. Under lower pressure, the gas viscosity increases with temperature, since elevated temperature degrees increase the gas molecules’ interactions, leading to reduced gas stream flow. An increase in viscosity is indicative of this effect. Conversely, as gases compress and their molecular distances decrease, they exhibit liquid behavior at higher pressures. Furthermore, the tight bonds that hold gas molecules together loosen as the temperature rises, which reduces the viscosity of gas flow^3^. These variations in natural gas viscosity as a measure of reservoir temperature and pressure, in addition to an extensive spectrum of possible gas combinations, impede some degree of measurement error^3,12^. Therefore, it is calculated theoretically by leveraging equations of state (EOS) relevant to the reduced/critical properties and gas composition, or empirical correlations as a dependence of temperature, pressure, molecular weight, gas density, and gravity^1,2^. When working with gas mixtures, EOS may yield low accuracy, whereas empirical correlations can involve one or two-step techniques^1^.

Several pieces of literature discuss gas viscosity correlations and mathematical models. Fayazi et al.^6^ and Dargahi-Zarandi et al.^3^ summarize the most common gas viscosity correlations. In the following discussion, the authors detailed the published correlations that considered gas viscosity.

Bicher Jr and Katz^13^ introduced the first gas viscosity correlation as a function of pressure ranges from (400–500) psia, temperature (77–437 °F), and molecular weight with a 5.8% average deviation. They employed a rolling ball inclined tube viscometer to estimate the viscosities of CH_4_, C_3_H_8_, and four binary mixtures of CH_4_. Smith and Brown^14^ a_ppr_oximate the fluid viscosity through EOS. Their theorem is based on the similarity of the physical properties of different substances with similar values of reduced temperature (*T*_*r*_) and pressure (*P*_*r*_). Their correlation was based on replacing the molecular weight with the molar average molecular weight and replacing reduced temperature and pressure with pseudo-critical properties. Their results showed that the correlation is not applicable for methane and gives a better estimation for liquid than gas viscosities. Smith also stated that the correlation is well-fitted for C_2_H_6_ and higher paraffin viscosities. Comings et al.^15^ proposed a graphical correlation using data of CO_2_, N_2_, NH_3_, H_2_O, CH_4_, C_3_H_8_, and C_4_H_10_ viscosities to approximate the gas viscosity through reduced pressure (*P*_*r*_) charts and viscosity ratio. They assessed the viscosity of N_2_, CO_2_, CH_4_, C_2_H_6_, and C_3_H_8_ at (*P* = 14.7–14,196 psia and, *T* = 14 to 374 °F) and improved their previous correlation by manipulating the analogy between the kinetic pressure and the viscosity. Their improved correlation was found to be applicable with pure gases and the results showed that the highest P and T for the CH_4_ viscosity are 2514 psia and 203 °F respectively. Also, their correlation gives a 10–12% error for C_2_H_6_ viscosity at (*P*_*r*_ > 2 psia). Carr et al.^16^ presented a correlation known as (CKB) as a function of *P*_*pr*_*, T*_*pr*_, and viscosity ratio, to predict the gas viscosity at (*T* = 32–400 °F, *P* < 1200 psia, and gas gravities = 0.55–1.55). Their correlation is built upon 30 data points including the measurements of pure components such as N_2_, CO_2_, CH_4_, C_2_H_4_, C_3_H_8_, and natural gases. Jossi et al.^17^ built a correlation referred to as JST for calculating the gas mixture’s viscosity based on experimental measurement of pure components including Ar, N_2_, O_2_, CO_2_, SiO_2_, (C_1_–C_5_) hydrocarbons. The correlation between the reduced density $({\rho }_{r})$ and the residual viscosity modulus ($({\mu }_{g}-{\mu }^{*})\varepsilon$ is the basis of the suggested correlation and the gas properties including *P*_*c*_*, T*_*c*_, density, and molecular weight are considered inputs. Lohrenz et al.^18^ developed an empirical correlation referred to as LBC for gas mixture viscosity estimation. Their model used the same equation and coefficient for pure fluid in the Jossi et al.^17^ correlation and they concluded that their model is highly reliant on the density measurements^2^. Dempsey^19^ approximates the gas viscosity ratio at a specified pressure to the gas viscosity at ambient conditions. Lee et al.^20^ derived a correlation relevant to gas density, temperature, and gas molecular weight to forecast gas viscosities at reservoir conditions^2^. The correlation is accurate for estimating the viscosity of natural gas below HPHT. Londono^21^ optimized both the JST and LGE correlations using a total of 13,656 data points and anticipated two models for gas viscosity prediction. Then another new optimized empirical formula for gas viscosity relevant to the gas density and temperature was developed. Jeje and Mattar^22^ paralleled the LGE correlation to Carr et al.^16^ for sour and sweet gases. The results showed that the two correlations give identical results for sweet gases. Whereas, for sour gases, there is a significant difference between the compared correlations. Sutton^23^ correlated viscosities of gas condensate and associated gases as a function of CH_4_, C_3_H_8_, CH_4_/C_3_H_8_, CH_4_/C_4_H_10_, CH_4_/C_10_H_22_, and natural gas viscosity. They estimated the gas condensate behavior by utilizing the CH_4_/C_10_H_22_ binary mixtures. Viswanathan^24^ implemented NIST values at (*T* = 100–400°F and *P* = 5000 to 30,000 psia) to improve LGE correlation at HPHT conditions. Ohirhian and Abu^25^ estimated the natural gas viscosity using experimental data values obtained from routine PVT analysis of the Nigerian crude oil at reservoir conditions (*P*_*res*_ = 144–4100 psia and *T*_*res*_ = 130–220 °F). The correlation was also compared with the viscosity values derived from Carr et al.^16^ charts and Lee et al.^20^ viscosity equation, and the results revealed that the new correlation showed precise results.

Recently, computational techniques, mathematical models, and equations of states (EOSs) have been implemented in various industrial applications including petroleum processes^26,27^. Various predictive models including conventional and hybrid ANNs models, and adaptive neuro-fuzzy inference system models^6^ have been estimated. Londono et al.^28^ established a gas viscosity correlation relevant to gas density, and reservoir temperature, considering non-hydrocarbon compounds including carbon dioxide, helium, and nitrogen, with an AARE of 3.05%. Quraishi and Shokir^2^ developed two gas viscosity and density models through genetic programming (GP) and alternating conditional expectation (ACE) algorithms using 4445 datasets with an AARE of 3.95% and 5.4%, respectively. Yang et al.^29^ predict gas viscosity for gas samples containing high carbon dioxide concentrations. The formulated correlation is built with 1,539 data points of 9 natural gas samples measured at 250 to 450 K and 0.10 to 140 MPa. The correlation reported an (ARE < 0.98%) between the calculated and the experimental values. Dargahi-Zarandi et al.^3^ developed a robust model algorithm based on the GMDH neural network for estimating sour and natural gas viscosity as a function of *T*_pr_, *P*_pr_, molecular weight, and density using more than 3800 data points reported in the literature. Rostami et al.^1^ modeled the natural gas viscosities using integrated smart intelligent strategies with optimized algorithms, where more than 3800 data points were screened for their (*T*_*pr*_, *P*_*pr*_, and molecular weight) to develop the required models.

The fuzziness, non-linearity, and complexity of the reservoir behavior necessitate a prevailing tool to overwhelm these challenges^6,12^. In this regard, soft computations and machine learning gained incremental spread in petroleum and chemical engineering issues^12,30–33^. These approaches include various predictive models such as ANN, GP, ANFIS, SVM, and their optimized hybrids^1,34–36^. ANNs used to outperform multivariate nonlinear regression processes^37^. This work introduces the utilization of machine learning algorithms to precisely estimate the viscosities of CH_4_, N_2_, and gas mixture under normal and harsh conditions relevant to pressure, temperature, and gas density based on the measured PVT database reported in the literature to develop and test the models. The network’s prediction was validated against the published one. The results displayed better performance for the developed models concerning viscosities of CH_4_, N_2_, and gas mixture. Seven machine learning techniques specifically Linear Regression, Random Forest (RF), Decision Trees (DT), Gradient Boosting, K-Nearest Neighbors (KNN), Nu Support Vector Regression (NuSVR), and artificial neural network (ANN) were applied for the prediction of methane (CH4), nitrogen (N_2_), and natural gas mixture viscosities. The reasons behind using the considered ML types in modeling the gas viscosity lie in the viscosities of gas mixtures exhibit non-linear behavior under different conditions. Moreover, using machine learning models can help uncover and model these complicated relationships. The considered Machine learning models offer flexibility in modeling different types of data and handling various inputs. Machine learning models can handle multiple input variables and their interactions efficiently, potentially providing accurate predictions across diverse scenarios. Furthermore, machine learning is a data-driven approach, meaning it can learn patterns and relationships directly from the available data.

Unlike most of the literature on ML models, the viscosity of three distinct gases is predicted using seven different machine-learning techniques. Previous research focuses only on the modeling of gas mixture viscosity^2,38^. In addition, seven different machine-learning techniques were utilized for the prediction of gas viscosity. The previous research was mostly directed only at artificial neural networks (ANNs) and adaptive neuro-fuzzy inference systems (ANFIS)^1,2,39,40^. To the best of the author’s knowledge limited studies have applied ML techniques for Methane and Nitrogen viscosity prediction. Almost all viscosity models developed using ML algorithms are suitable for gas mixtures. Moreover, no work has been quoted in the literature that predicts methane, nitrogen, and natural gas mixture viscosities under normal and harsh conditions using seven distinct machine-learning techniques.

## Materials and methods

**Figure 1:**
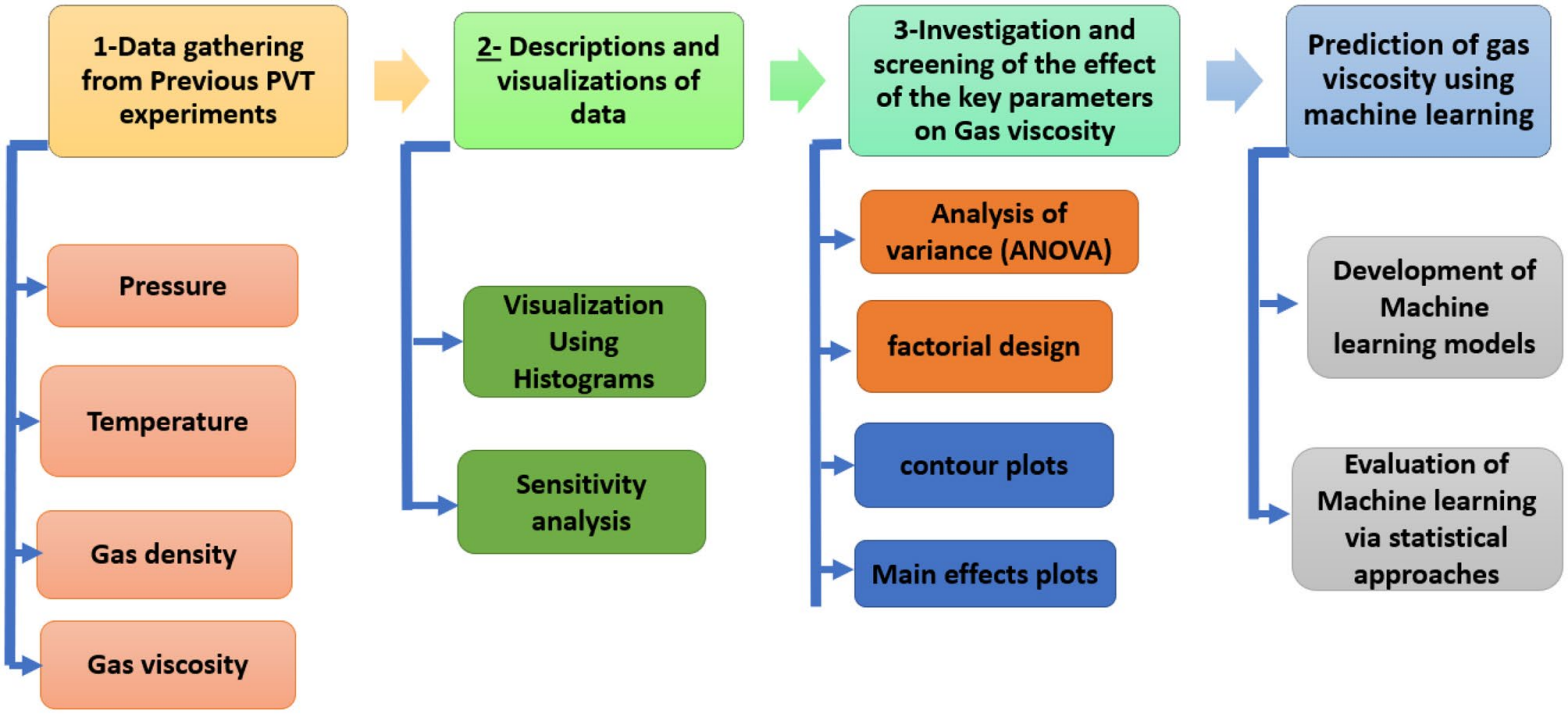
Framework of research methodology.

This article utilizes statistical approaches for analyzing the results of published PVT experiments for CH_4_, N_2_, and gas mixture at standard conditions as well as severe conditions relevant to high temperature and pressure. The goal is to clarify the significance of important elements (pressure, temperature, and gas density) on the viscosity of CH_4_, N_2_, and gas mixture during PVT experiments. The research’s methodological approach is shown in Fig. 1. Initially, the published experimental PVT data and results were gathered. Additionally, the database was shown and explained using a histogram including pressure, temperature, and gas density together with the viscosity of CH_4_, N_2_, and gas mixture. Moreover, relationships between viscosity versus pressure, temperature, and gas density were established using sensitivity analysis, analysis of variance, factorial design, contour plot, and main effects plot. Finally, ML algorithms were applied to predict the viscosity of CH_4_, N_2_, and gas mixture at standard and harsh conditions of temperature and pressure based on PVT data experiments.

The novelty of this research exists in the approach to present an advanced Machine learning model to predict methane, nitrogen, and natural gas mixture viscosities under a wide range of data. More than 4304 datasets from real experimental data utilizing pressure, temperature, and gas density were employed for developing ML models. Moreover, the article exhibits additional statistical approaches by which relationships between viscosity versus pressure, temperature, and gas density were established. The article also provides three new ANN-based correlations related to methane, nitrogen, and natural gas mixture viscosities. Therefore, this research is remarkable in its approach, as it extensively predicts methane, nitrogen, and natural gas mixture viscosities using seven distinct machine-learning algorithms for the first time.

### Exploratory data analysis

Establishing an accurate predictive machine learning model (ML) strongly relies on the quality of the input dataset. The accurate prediction capability of ML models is determined by the effect of input feature significance on the output parameters. Analysis of variance (ANOVA) test was employed using Minitab software, to analyze several variables of the generated model. The relationship between the viscosities of the methane, nitrogen, and natural gas mixture and the number of independent variables was examined using the values of P and F. If the *P*-value < 0.05, the factor’s statistical importance is proven. To highlight the impact of different independent parameters even more on the methane, nitrogen, and natural gas mixture viscosities, factorial, contour plots, and main effects plots were analyzed.

### Machine learning (ML) models

Seven machine learning algorithms have been applied to predict methane, nitrogen, and natural gas mixture viscosities under normal and harsh conditions: Linear Regression, Decision Trees (DT), Gradient Boosting, Random Forest (RF), Nu Support Vector Regression (NuSVR), Artificial Neural Network (ANN), and K-Nearest Neighbors (KNN). The definitions of such ML are as follows:

1. *Linear regression* is a statistical technique that uses the association between two or more quantitative variables to predict a response or outcome variable based on the others^30^. This methodology is frequently utilized in numerous disciplines.
2. *The decision tree (DT)* is a flowchart-like model, where the internal node signifies an attribute test, each branch signifies a test result, and each leaf node provides a class label or prediction^41^. The DT is simple to grasp and analyze, and it can deal with both category and numerical data. Furthermore, the DT can be utilized for classification and regression problems, as well as a foundation for more complex ensemble approaches^42^.
3. *Random forest* is an ensemble learning method that allows forecasts by combining many Decision trees. It generates many Decision trees and outputs the mode or mean prediction of each tree^42,43^. By incorporating randomization into the tree-building process, the Random Forest avoids the overfitting propensity of Decision trees and increases generalization.
4. *Gradient boosting* is a popular ensemble method for integrating numerous weak models to build a strong prediction model^44^. Gradient boosting also contains regularization techniques to prevent overfitting, as well as parallel processing and tree pruning, making it efficient and very accurate. It has gained traction in numerous machine learning competitions and is frequently utilized in the industry.
5. *Support vector regression* is a machine learning theory that is founded on the notion of structural risk minimization^45^. Support vector regression methods are a promising data mining and knowledge-finding technique. In the 1990s, support vector regression was introduced as a nonlinear solution for regression analysis. It was created for pattern recognition tasks using the paradigm of statistical learning theory. Support vector regression has gathered considerable interest as it has been recognized to be very robust for modeling numerous complex problems^42^.
6. *K-nearest neighbour (KNN)* is a widely used statistical method for pattern recognition and classification that is quick and easy to use in several different fields^46^. K-Nearest Neighbour is built on the premise that similar input points will produce similar outputs. These related input points are first sorted into groups in n-dimensional space. For new point output, the k points that are closest to the new point are selected, and the group with the highest number of points near the new point is found by analysis. After that, the points are tallied, and the group with the greatest number of points within the hypercube is approximated. Finally, the new point is allocated to that group, and the cluster prediction method is used to anticipate its output.
7. *Artificial neural network (ANN)* is an ML model that was stimulated by the anatomy of the human brain^30,47^. The ANN is made up of linked neurons that are organized into layers^31^. Each neuron processes input data and sends it to the next layer, allowing for complicated computations^48^. The ANN may learn from a variety of data and be applied to tasks such as classification, regression, and pattern recognition. Fully connected neural networks are useful in petroleum-related applications where the inputs and outputs have nonlinear correlations^32,49,50^.

### Models evaluation and error analysis

For examining the model applicability and precision, several traditional statistical measures and graphical error analyses are used to assess the accuracy, validity, and reliability of the produced models, and predict the performance of the created machine learning algorithms^12,51^. Furthermore, relative error distribution graphs and Cross-plots were also employed.

#### Statistical error analysis

It is a vital tool to assess the model’s performance relative to the experimental values^52^. In the current work, the developed paradigms’ reliability was screened by some statistical principles involving (ARE%)^1^, (AARE%), (RMSE), (SD), and (R^2^)^12^. The following formulas are used to compute these statistical parameters^3,4,37^.

1 $$ARE\% = \frac{100}{N}\sum\limits_{i = 1}^{N} {\left( {\frac{{\mu_{i}^{\exp } - \mu_{i}^{cal} }}{{\mu_{i}^{\exp } }}} \right)}$$

2 $$AARE\% = \frac{100}{N}\sum\limits_{i = 1}^{N} {\left| {\frac{{\mu_{i}^{\exp } - \mu_{i}^{cal} }}{{\mu_{i}^{\exp } }}} \right|}$$

3 $$SD = \left[ {\frac{1}{N - 1}\sum\limits_{i = 1}^{N} {\left( {\frac{{\mu_{i}^{\exp } - \mu_{i}^{cal} }}{{\mu_{i}^{\exp } }}} \right)}^{2} } \right]^{1/2}$$

4 $$RMSE = \left[ {\frac{{\sum\limits_{i = 1}^{N} {\left( {\mu_{i}^{\exp } - \mu_{i}^{cal} } \right)^{2} } }}{N}} \right]^{1/2}$$

5 $$R^{2} = 1 - \frac{{\sum\limits_{i = 1}^{N} {(\mu_{i}^{\exp } - \mu_{i}^{cal} )^{2} } }}{{\sum\limits_{i = 1}^{N} {\left[ {\mu_{i}^{\exp } - avg(\mu_{i}^{cal} )} \right]^{2} } }}$$

6 $$MAD = \max \left( {\frac{{\mu_{i}^{\exp } - \mu_{i}^{cal} }}{{\mu_{i}^{\exp } }}} \right)$$

#### Graphical error evaluation

The model performance is visualized through graphical error analyses, where crossplots and error distribution are reported^3^. Three visualization techniques, including cross plots, cumulative frequency plots versus AARE%, and error distribution curves, were implemented to screen the model accuracy^4^.

##### Cross plot

The predicted/experimental data points are plotted versus each other to assess the model’s competence in the prediction of the experimental one^3^. The diagram capability is evaluated through the grade of data gathering around the equality line and the deviation from the 45° line.

##### Error distribution diagram

It displays the error distribution around the zero-error line^3,4^, where the benchmark is the neighboring data amount around the zero-deviation line^1^. The gathering of predictions around the zero-error line is very reasonable.

##### Cumulative frequency versus AARE%

It is the sum of classes in a frequency distribution (i.e. adding up a value and all of the values that came before it^4^.

## Results and discussion

### Data acquisition and analysis

Reliable data were gathered from the published literature^51^. The dataset collected from literature relevant to the viscosities of CH_4_, N_2_, and natural gas mixture with their correlating parameter ranges are indicated in Table 1. Methane and nitrogen viscosities are correlated relevant to reservoir temperature (T_res_) and pressure (P_res_), while the viscosity of the natural gas mixture is correlated relative to reservoir pressure, temperature, and gas density. The collected data were treated to develop an ANN model for viscosity calculation.

**Table 1:** Ranges of correlating parameters for methane, nitrogen, and natural gas viscosities.

Parameters	Methane	Nitrogen	Natural gas mixture
No. of dataset	1664	1672	968
P, psia	14.7 to 25,015	14.7 to 24,515	14.7 to 25,000
T, °F	77 to 482 °F	− 343 to 1880	− 8.6 to 360
Gas viscosity, cp	0.011 to 0.07	0.006 to 0.391	0.01 to 0.121
Gas density, lb/ft^3^	….	….	0.02 to 12.016

**Figure 2:**
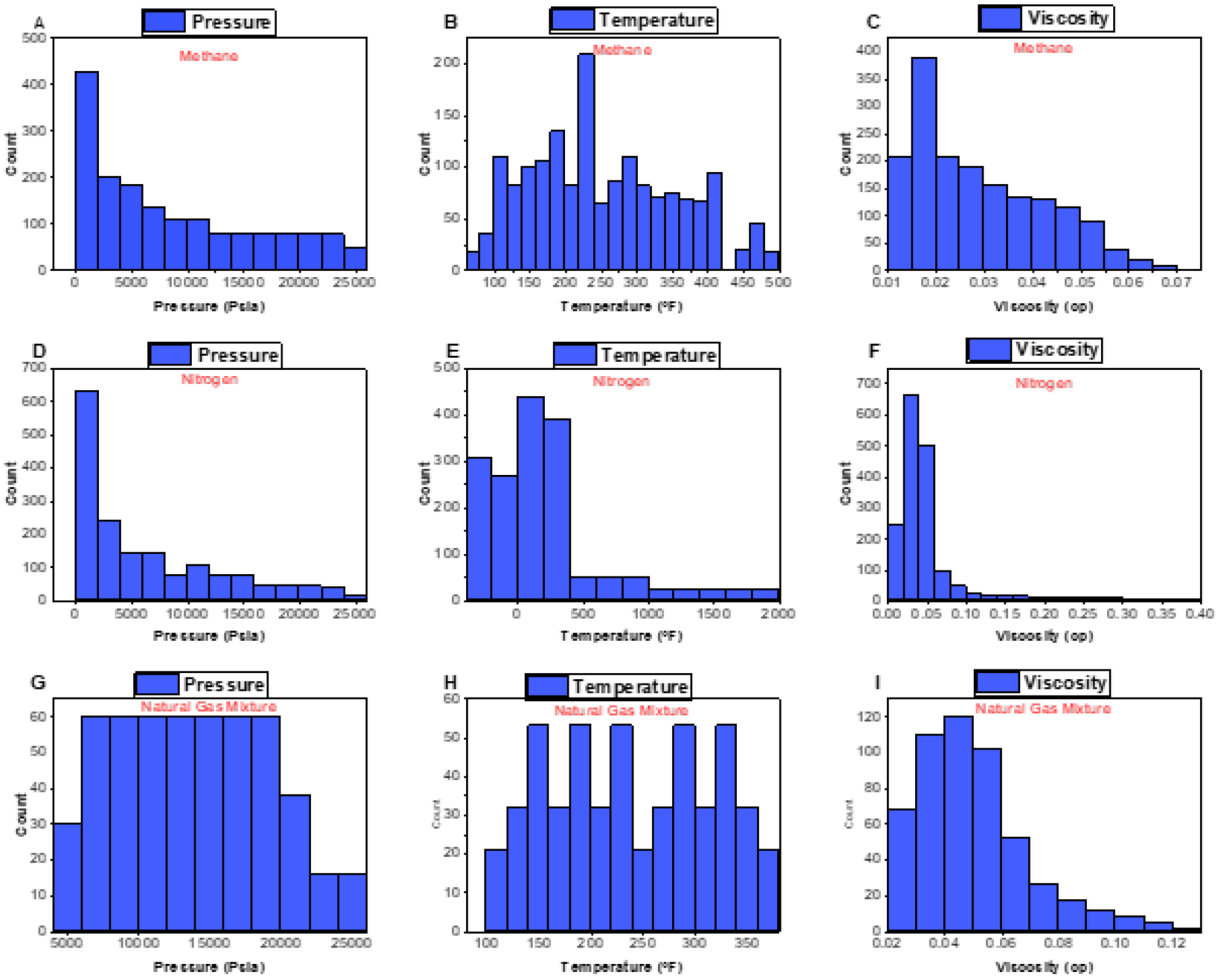
Histogram plots based on the gathered data from literature: (**A**) methane pressure, (**B**) methane temperature, (**C**) methane viscosity, (**D**) nitrogen pressure, (**E**) nitrogen temperature, (**F**) nitrogen viscosity, (**G**) natural gas mixture pressure, (**H**) natural gas mixture temperature, and (**I**) natural gas mixture viscosity data.

The dataset was graphically displayed, and the sampling distribution was explained using histograms. Figure 2A–C shows the histograms of the data sets for methane case (pressure, temperature, and viscosity) respectively. The datasets encompass a variety of pressure from 14.7 psia up to 25,000 psia (harsh conditions). However, most of the data points range from 14.7 and 10,000 psi. Furthermore, the data on temperature showed a large range of values (77–482 °F). The majority dataset of temperature falls between 100 and 350 °F. Regarding methane viscosity, the most common values varied from 0.011 to 0.05 cp. Figure 2D–F shows the histograms of the data sets for nitrogen viscosity. A variety of pressures are covered by the datasets, ranging from 14.7 psia up to 24,000 psia (harsh conditions) but most of the data points lie between 14.7 and 12,000 psi. Likewise, a large range was present in the temperature data (− 343 to 1880 °F). Most temperature data is in the range of − 340 to 350 °F. The most frequent values for methane viscosity ranged from 0.006 to 0.0.07cp. Figure 2G–I depict the histograms of pressure, density, temperature, and viscosity for mixture gas from the reported studies. The databank covers a range of pressure from 14.7 psia up to 25,000 psia (harsh conditions). However, most of the data points range from 14.7 to 10,000 psi. Similarly, the data on temperature showed a large range of values. (− 8.6 to 360 °F). The most frequent values for gas mixture viscosity ranged from 0.01 to 0.1cp. Furthermore, the density of gas mixture data had an extensive range of values (0.02 to 0.6 lb/ft^3^).

### Sensitivity analysis

Sensitivity analysis relies on the absolute relevancy factor (r) analysis and is used to evaluate the effect of input parameters on the viscosities of methane, nitrogen, and natural gas mixture^3,32^. The effect of the input parameter is directly correlated with its relevancy factor’s absolute value as follows^4^:

7 $$r(I_{j} ,p) = abs\left( {\frac{{\sum\limits_{i = 1}^{N} {(I_{j,i} - I_{j} )(p_{i} - \overline{p})} }}{{\sqrt {\sum\limits_{i = 1}^{N} {(I_{j,i} - I_{j} )^{2} \sum\limits_{i = 1}^{N} {(p_{i} - \overline{p})^{2} } } } }}} \right)$$

(*i*)_subscript_, The data point index; *Ij,i*, is jth input parameter; *Ij*, Corresponds to the average value of Ij, I; *P* and* Ṕ*, Correspond to the predicted value and its average, respectively.

**Figure 3:**
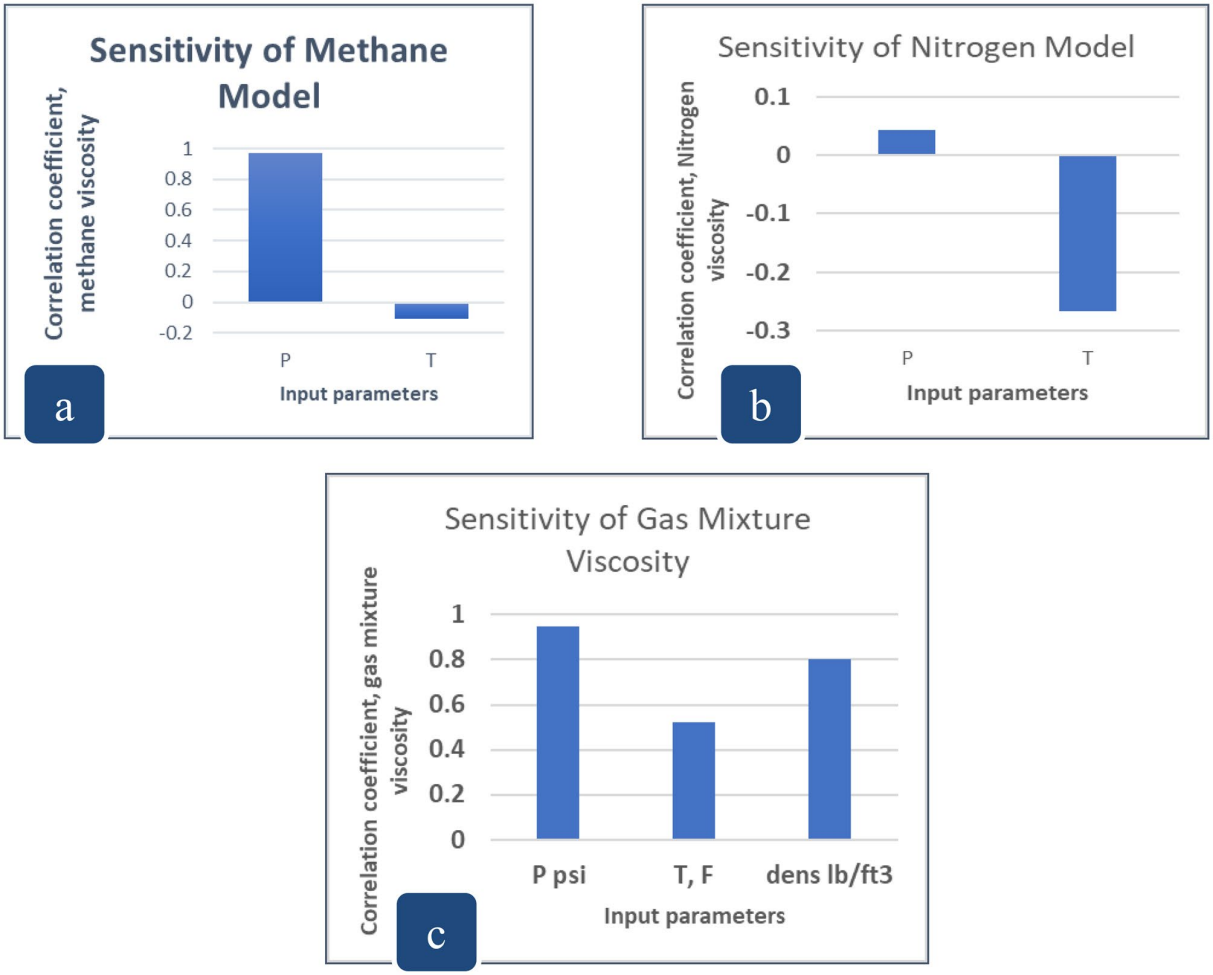
Sensitivity of (**a**) methane, (**b**) nitrogen, (**c**) natural gas mixture viscosities to pressure and temperature.

Nitrogen and methane viscosities are directly proportional to the pressure with a correlation coefficient of 4% and 97% respectively, and inversely proportional to temperature with a correlation coefficient of 27% and 10% respectively. The viscosity of the natural gas mixture is directly proportional to the pressure with a correlation coefficient of 95%, temperature with a correlation coefficient of 52%, and gas density with a correlation coefficient of 80%. Figure 3a–c display the implications of independent correlating parameters (pressure, temperature, and gas density) on the viscosity values of N_2_, CH_4_, and gas mixture, where (P, T) has the highest impact in the case of N_2_, CH_4_ viscosity, while gas density greatly affects in case of gas mixture viscosity^2,4^. Correlation coefficients range from (0–1), whereas 1.0 indicates a strong correlation, while 0.0 indicates no correlation between the input and the targeted variables^53,54^.

Sensitivity analysis aims to measure the model’s sensitivity towards the independent variables. The variable’s influence on the prediction decreases with decreasing percent values for the designated independent variables. The analysis results assign the selection of independent variable sets for more precise prediction. A low-impact variable can be removed. The relative impact of the trained set variables may vary more in different data sets. The statistical parameters of the utilized datasets modeling through this study are summarized in Table 2. The important parameters are skewness, and kurtosis which are defined as:

i. *Skewness* which signifies the property asymmetry degree relevant to its mean value. Skewness ranges from (− 1) to (0) to (+ 1), whereas zero skewness corresponds to the normal distribution, while (+ 1 and − 1) correspond to non-normal probability distribution.
ii. *Kurtosis* describes the distribution of data with the normal probability shape. The normal distribution has zero kurtosis. Furthermore, compared to the normal distribution, the positive and negative kurtosis correspond to a more peaked and flatter distribution^1^.

**Table 2:** Statistical parameters of datasets used to develop ANN model for methane, nitrogen, and natural gas mixture respectively.

Statistical error	Methane			Nitrogen			Natural Gas Mixture		
	P, psia	T, °F	Viscosity, cp	P, psia	T, °F	Viscosity, cp	P, psia	T, °F	Viscosity, cp
Mean	8491.934	248.0514	0.02896	6258.769	204.85	0.049	7674.291	152.743	6.145
Median	6264.7	230	0.025975	3626	134	0.038	6000	120	10.596
Mode	14.7	280	0.016	14.504	260	0.018	5000	8.6	11.07
Standard Deviation	7450.489	102.2796	0.013288	6450.345	468.183	0.048	7217.802	104.162	5.485
Sample Variance	55,509,785	10,461.11	0.000177	41,606,945.83	219,195.723	0.002	52,096,665	10,849.786	30.08
Kurtosis	− 0.79866	− 0.71048	− 0.57821	0.153	3.159	14.061	− 0.976	− 1.034	− 1.958
Skewness	0.687633	0.354249	0.670356	1.095	1.734	3.488	0.598	0.378	− 0.133
Range	25,000	405	0.05902	24,500.196	2223	0.386	24,986.28	351.4	12.014
Minimum	14.7	77	0.011	14.504	− 343	0.006	13.718	8.6	0.002
Maximum	25,014.7	482	0.07002	24,514.7	1880	0.391	25,000	360	12.016
Count	1664	1664	1664	1672	1672	1672	968	968	968

### Investigation and screening of the impact of key factors on the gas viscosity

The pressure and temperature values greatly influence the gas viscosity. Consequently, statistical approaches were utilized to screen the highest meaningful factors. These statistical analyses were performed using Minitab software^55^.

#### The analysis of variance (ANOVA)

ANOVA is a statistical approach used in analyzing many problems in the upstream oil industry^34,56^. In this regard, Methane, nitrogen, and mixture gas were the three types of gases whose viscosity was studied. ANOVA was used to obtain a quantitative interpretation of the investigated parameters. The results of ANOVA are exhibited in Tables 3, 4, and 5. As displayed in the first analysis (methane viscosity), P, and T have a significant impact on the viscosity of methane based on the *P*-value of to a lesser extent than 0.05. Furthermore, pressure displays the greatest F-value (119,178.47), which indicates the stronger influence of such a parameter compared with the temperature parameter. In contrast, for the second analysis (nitrogen viscosity), the temperature has a meaningful effect on the viscosity of nitrogen based on the *P*-value and the F-values of 0 and 25.99 respectively. According to the *P*-value of less than 0.05 for the third case (gas mixture), temperature, pressure, and gas density, all significantly affect the gas mixture’s viscosity. Furthermore, the pressure displays the highest F-value (33,426.02) which indicates the strongest influence of the pressure parameter compared with other parameters, which also reflects its significance on gas viscosity.

**Table 3:** Analysis of variance for methane viscosity.

Basis	DF	Adj. SS	Adj. MS	F-value	*P*-value
Model	3	0.290634	0.096878	53,210.57	0.000
Linear	2	0.258394	0.129197	70,961.97	0.000
P, psia	1	0.216983	0.216983	119,178.47	0.000
T, °F	1	0.010118	0.010118	5557.07	0.000
Two-way interactions	1	0.010529	0.010529	5783.12	0.000
P, psia*T, F	1	0.010529	0.010529	5783.12	0.000
Error	1660	0.003022	0.000002		
Lack-of-fit	1656	0.003022	0.000002	194.41	0.000
Pure error	4	0.000000	0.000000		
Total	1663	0.293656

**Table 4:** Analysis of variance for nitrogen viscosity.

Basis	DF	Adj. SS	Adj. MS	F-value	*P*-value
Model	3	0.29400	0.098000	45.66	0.000
Linear	2	0.19731	0.098654	45.96	0.000
P, psia	1	0.00354	0.003536	1.65	0.199
T, F	1	0.05579	0.055788	25.99	0.000
Two-way interactions	1	0.00003	0.000032	0.01	0.903
P, psia*T, F	1	0.00003	0.000032	0.01	0.903
Error	1668	3.58008	0.002146		
Total	1671	3.87408

**Table 5:** Analysis of variance for gas mixture viscosity.

Basis	DF	Adj SS	Adj MS	F-value	*P*-value
Model	7	0.188610	0.026944	8427.54	0.000
Linear	3	0.179757	0.059919	18,741.28	0.000
P, psia	1	0.106868	0.106868	33,426.02	0.000
T, F	1	0.019430	0.019430	6077.24	0.000
gas density, lb/ft3	1	0.022949	0.022949	7177.97	0.000
Two-way interactions	3	0.010632	0.003544	1108.52	0.000
P, psia*T, F	1	0.002622	0.002622	820.14	0.000
P, psia*gas density, lb/ft3	1	0.004526	0.004526	1415.48	0.000
T, F*gas density, lb/ft3	1	0.001919	0.001919	600.19	0.000
3-way interactions	1	0.000303	0.000303	94.65	0.000
P, psia*T, F*gas density, lb/ft3	1	0.000303	0.000303	94.65	0.000
Error	512	0.001637	0.000003		
Total	519	0.190246			

#### Factorial design

**Figure 4:**
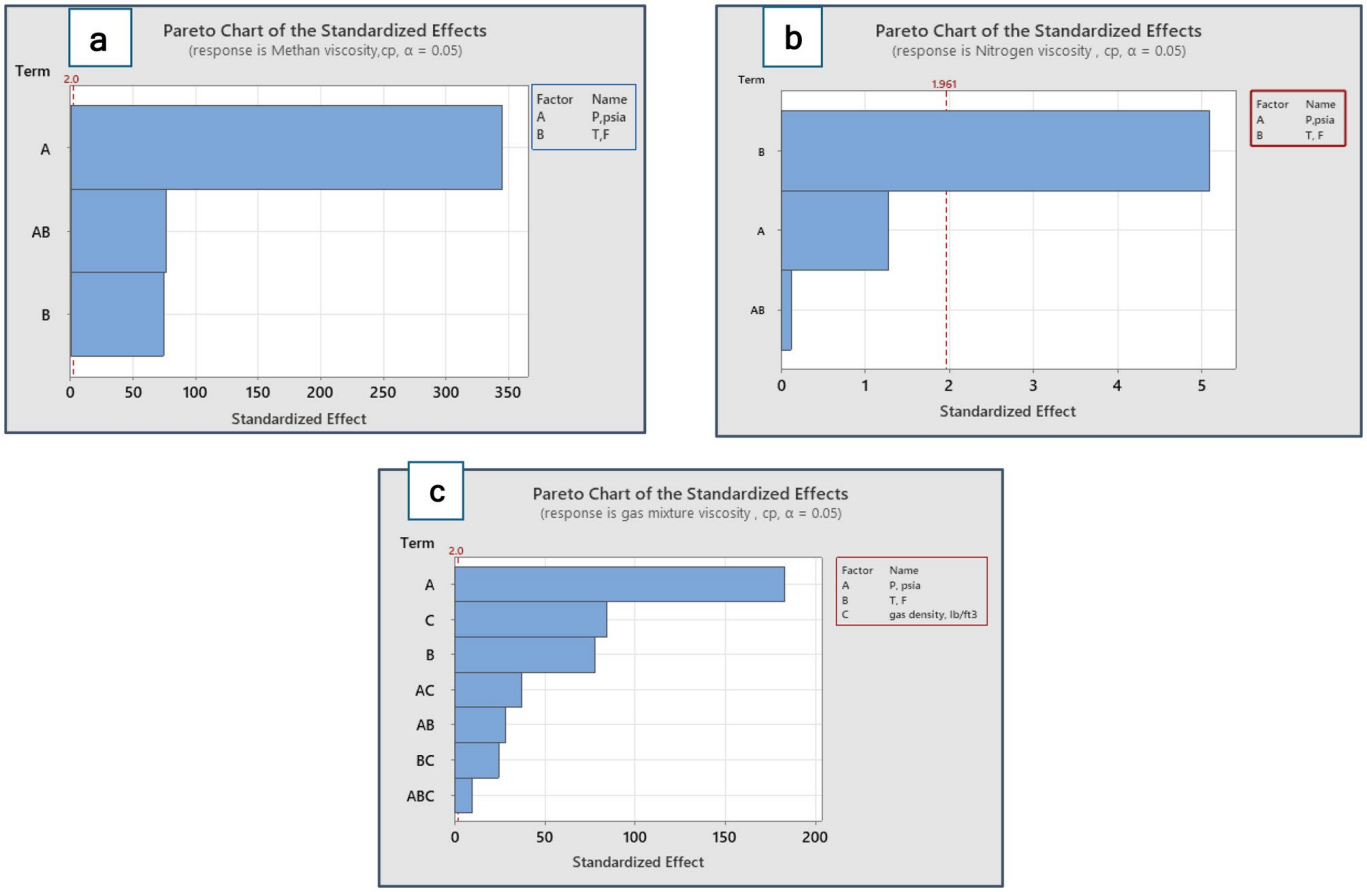
Pareto chart for results of (**a**) methane viscosity, (**b**) nitrogen viscosity, and (**c**) gas mixture viscosity.

Factorial design is an important statistical method for optimizing and simplifying laboratory experiments by investigating the effects of multiple controllable factors on interest responses^34,57^. These statistical evaluations of gas viscosity were conducted using Minitab software^55^. Additionally, the results of the factorial design are shown in the Pareto chart. The Pareto plot uses horizontal bars to show the influences of the parameters from maximum impact to smallest impact. To further indicate which factors are statistically significant, a reference line is drawn on the Pareto chart. In the current analysis, the assessment was achieved using pressure, temperature, and gas density. The factorial design was performed for the three cases: methane, nitrogen, and gas mixture. The factorial design-generated Pareto chart for the methane viscosity results is shown in Fig. 4a. Pressure is found to have the most significant effect on the performance of methane viscosity. Figure 4b describes the Pareto chart for the results of nitrogen viscosity, where temperature was found to have the most dominant performance in nitrogen viscosity. Figure 4c exhibits the Pareto chart generated by factorial design for the results of mixture gas viscosity. According to the Pareto graph, the factors that do better than the reference line are statistically significant. Therefore, it is apparent that pressure, gas density, and temperature were found to be the leading parameters respectively in the viscosity of the gas mixture. In conclusion, the factorial design statistical analysis agrees with the ANOVA test results.

#### Contour plots

**Figure 5:**
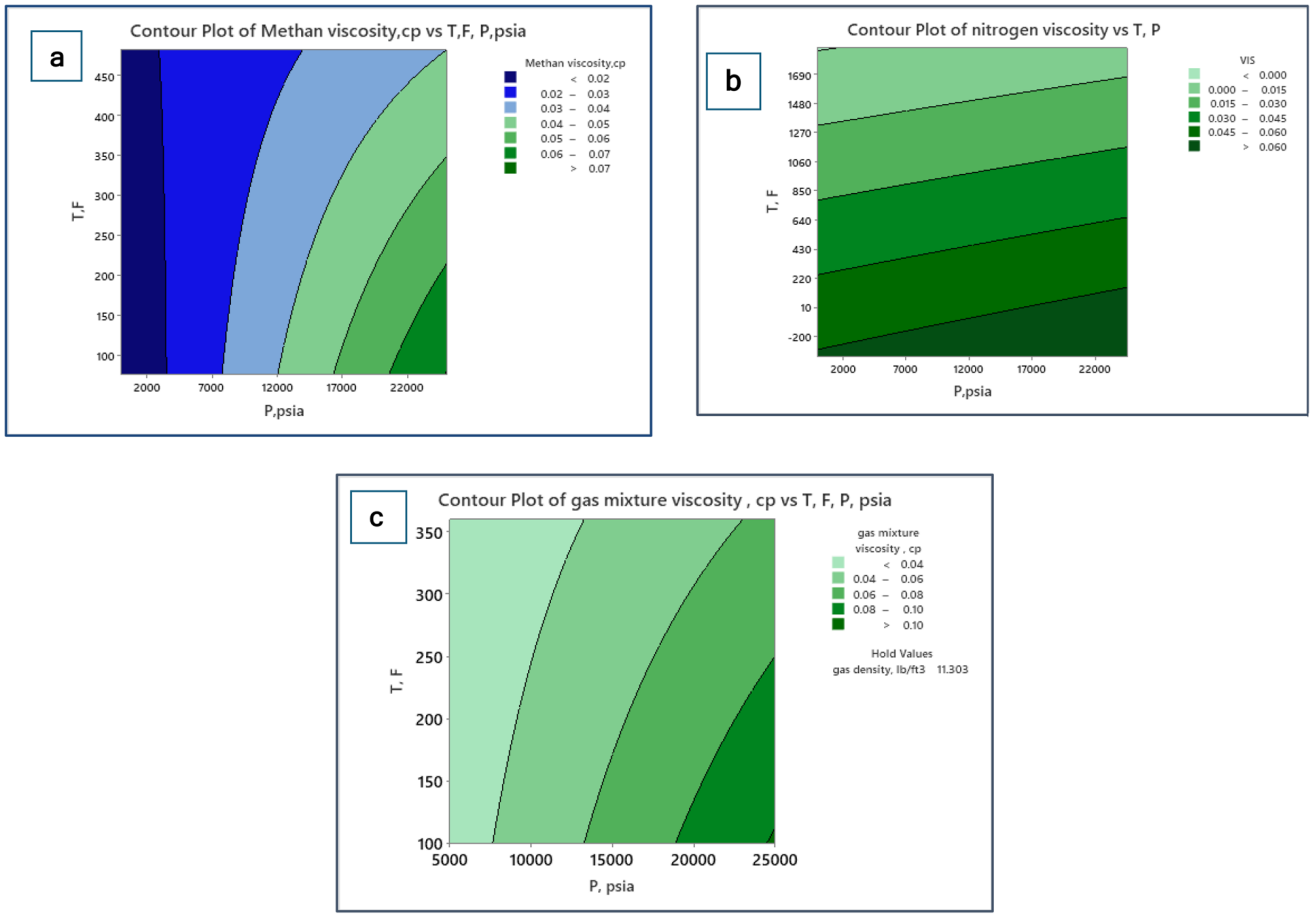
Contour map of viscosity correlated with T and P: (**a**) methane viscosity, (**b**) nitrogen viscosity, and (**c**) gas mixture viscosity.

A contour plot was employed to evaluate the influence of input variables on methane viscosity. Two parameters’ results were scrutinized concurrently on the methane viscosity values. Figure 5a shows the contour plot for methane viscosity to analyze the mutual effects of pressure and temperature. The contour plot reveals that the highest values of methane viscosity > 7.0 cp were found in the elevated pressure (above 22,000 psi) and low-temperature values. In contrast, the lowest values of methane viscosity < 0.02 cp were found in the low pressure (above 3000 psi) and all ranges of temperatures. Likewise, Fig. 5b shows the surface plot for nitrogen viscosity. The valley in the lower region of the graph represents the highest values of nitrogen viscosity > 0.06 cp, which represents all ranges of pressure and lower values of temperature. At the constancy of pressure and increasing temperature, the nitrogen viscosity moves to the lower regions of viscosity. Therefore, the upper valley of the graph represents the lowest values of nitrogen viscosity, which represents all ranges of pressure and the highest values of temperature. Likewise, Fig. 5c shows the mapping of pressure and temperature for mixture gas viscosity represented by a contour plot. As shown in the contour plot, the region of the left part of the graph represents the lowest values of nitrogen viscosity < 0.04 cp, which represents all ranges of temperature and lower values of pressure. Additionally, the corner in the lower right region of the graph represents the highest values of gas mixture viscosity > 0.1 cp, which represents higher values of pressure and low-mid values of temperature.

#### Main effect plots

**Figure 6:**
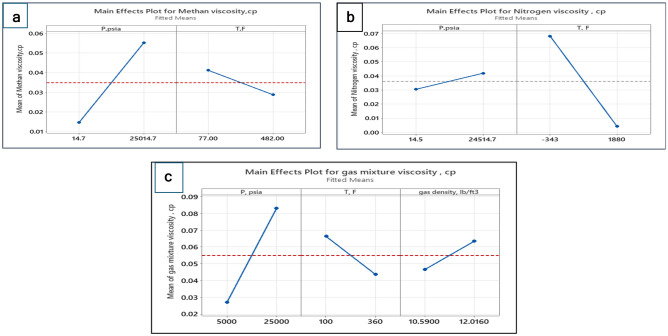
Main effect plot: (**a**) methane viscosity, (**b**) nitrogen viscosity, and (**c**) gas mixture viscosity.

Figure 6a shows the main effects plot for the methane viscosity. The red line horizontal line represents the mean value of methane viscosity. At a low-pressure level, the viscosity is below the mean value then methane viscosity increases with increasing pressure until reaches its maximum value at the highest level of pressure. In contrast, at a low-temperature level, the methane viscosity is above the mean value, then methane viscosity decreases with increasing temperature until reaches its lowest value at the highest level of temperature. The graph implies that pressure is the greatest powerful factor in methane viscosity. Likewise, the effects of P and T on nitrogen and gas mixture viscosity (Fig. 6b,c) respectively exhibit the same performance as in the case of methane. Furthermore, at a low level of gas density, the viscosity is lower than the mean value, then gas mixture viscosity improves with rising the pressure until reaches its maximum value at the highest level of gas density.

### Development of machine learning (ML) models

In pursuit of accurate gas viscosity predictions crucial for optimizing petroleum processes, we undertook a comprehensive investigation employing a diverse set of machine learning models. These models were chosen for their ability to capture intricate nonlinear relationships and patterns within the available complex datasets. In the development of the current model, 1664, 1672, and 968 data points were collected to model the viscosities of CH_4_, N_2_, and natural gas mixture respectively. The collected database was divided randomly into three subgroups as follows^4^.

i. *Training set* is used to control the weights and biases of the network to reduce training errors and identify the best predictive model. Once the difference between the model targets and outputs is minimized, the model adjusts biases and weights to generate the proper networks between the input and output.
ii. *Validation set* to prevent the likelihood of overfitting in training by screening the generalization of the process, where the inputs and target were normalized in the range of [1, + 1] by applying the subsequent formula^37^:
  8 $$p_{n} = \frac{{2(p - p_{\min } )}}{{(p_{\max } - p_{\min } )}} - 1$$
iii. *Testing set* An invisible, independent set is used to evaluate the trained network model’s performance.

#### Linear regression

**Figure 7:**
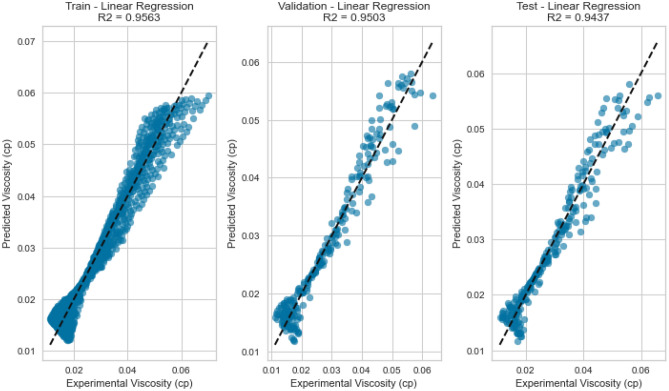
Cross plot for actual versus predicted values of methane viscosity (linear regression model).

**Figure 8:**
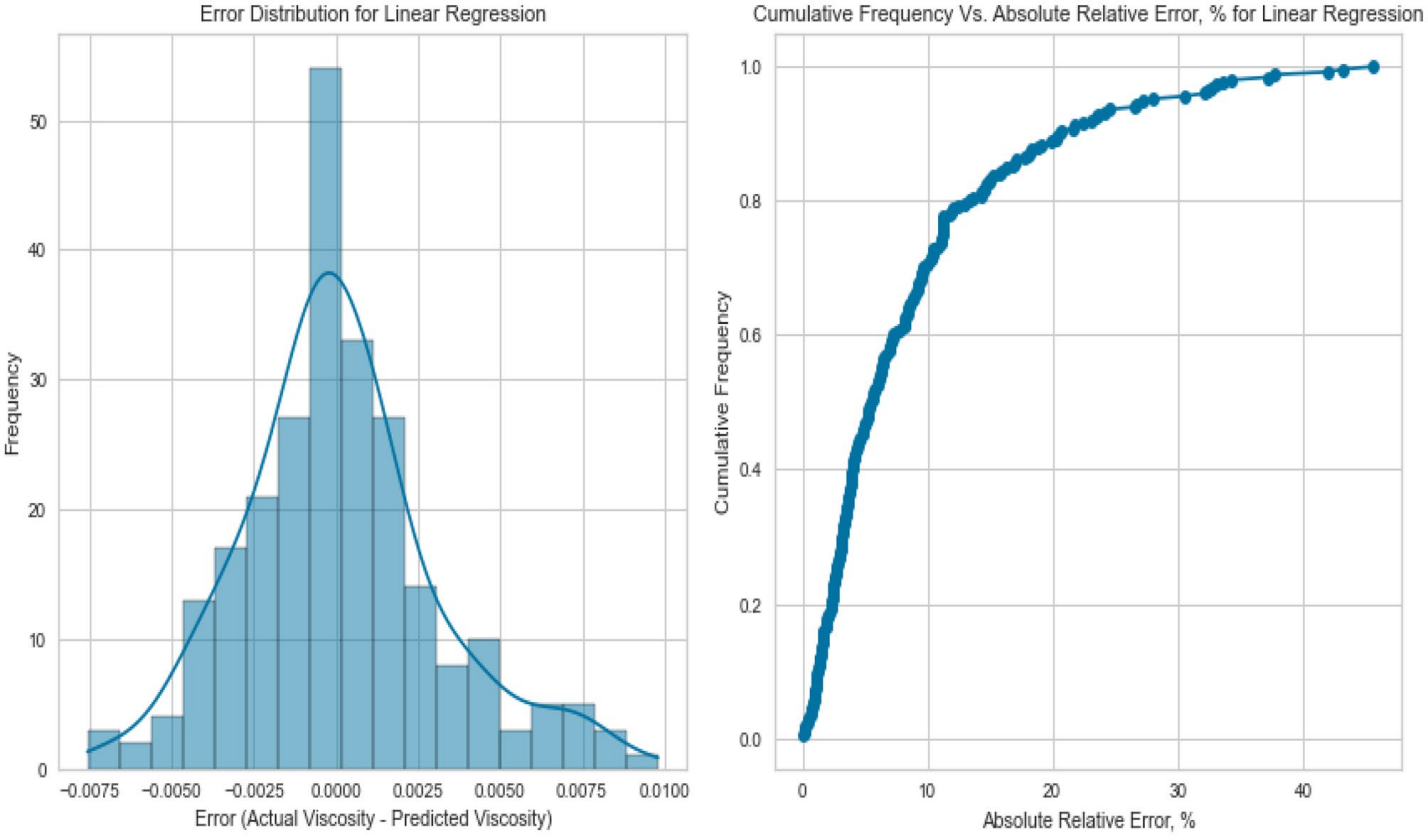
Error distribution for linear regression model (methane case).

**Figure 9:**
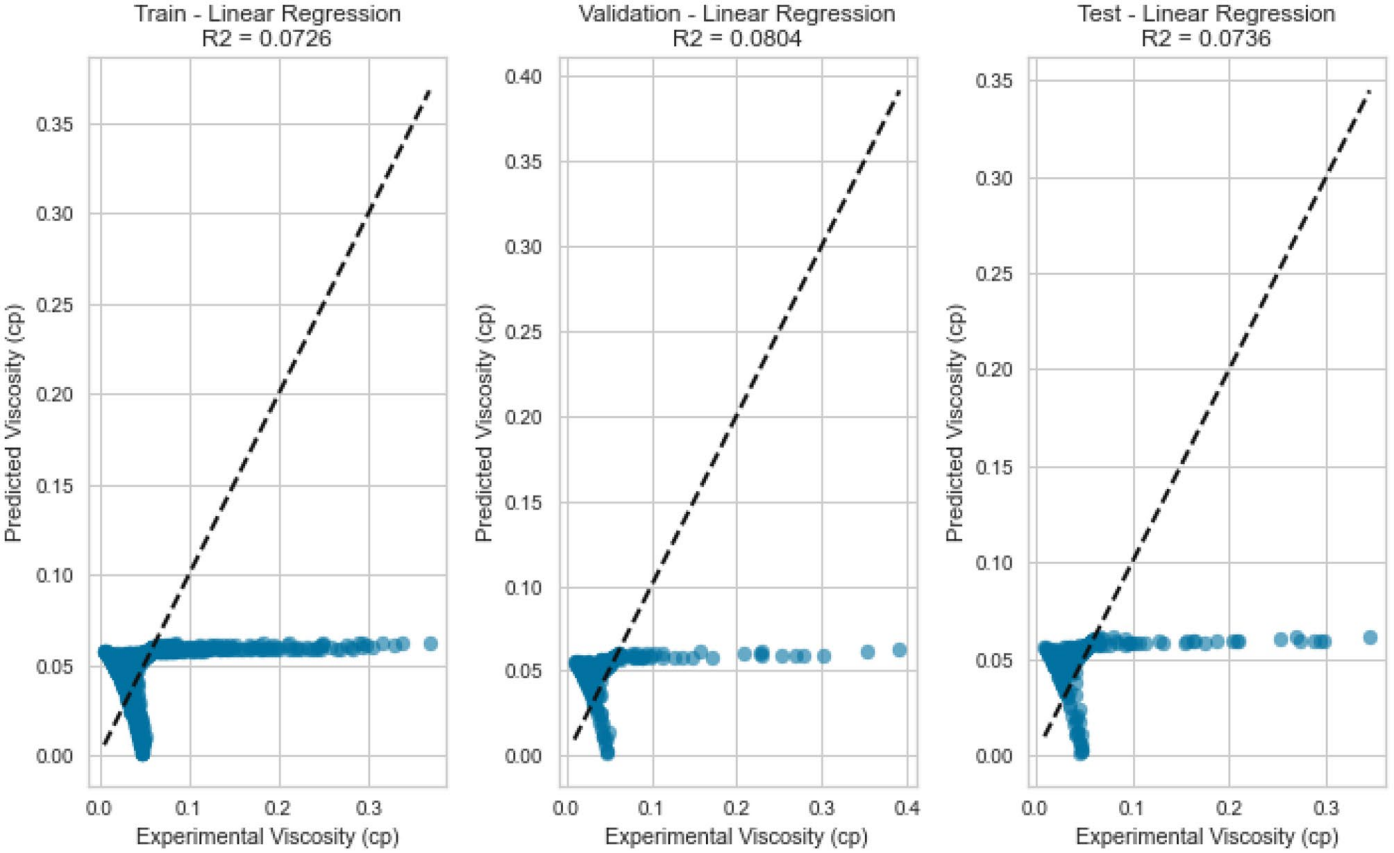
Cross plots of actual versus predicted values for nitrogen viscosity (linear regression model).

**Figure 10:**
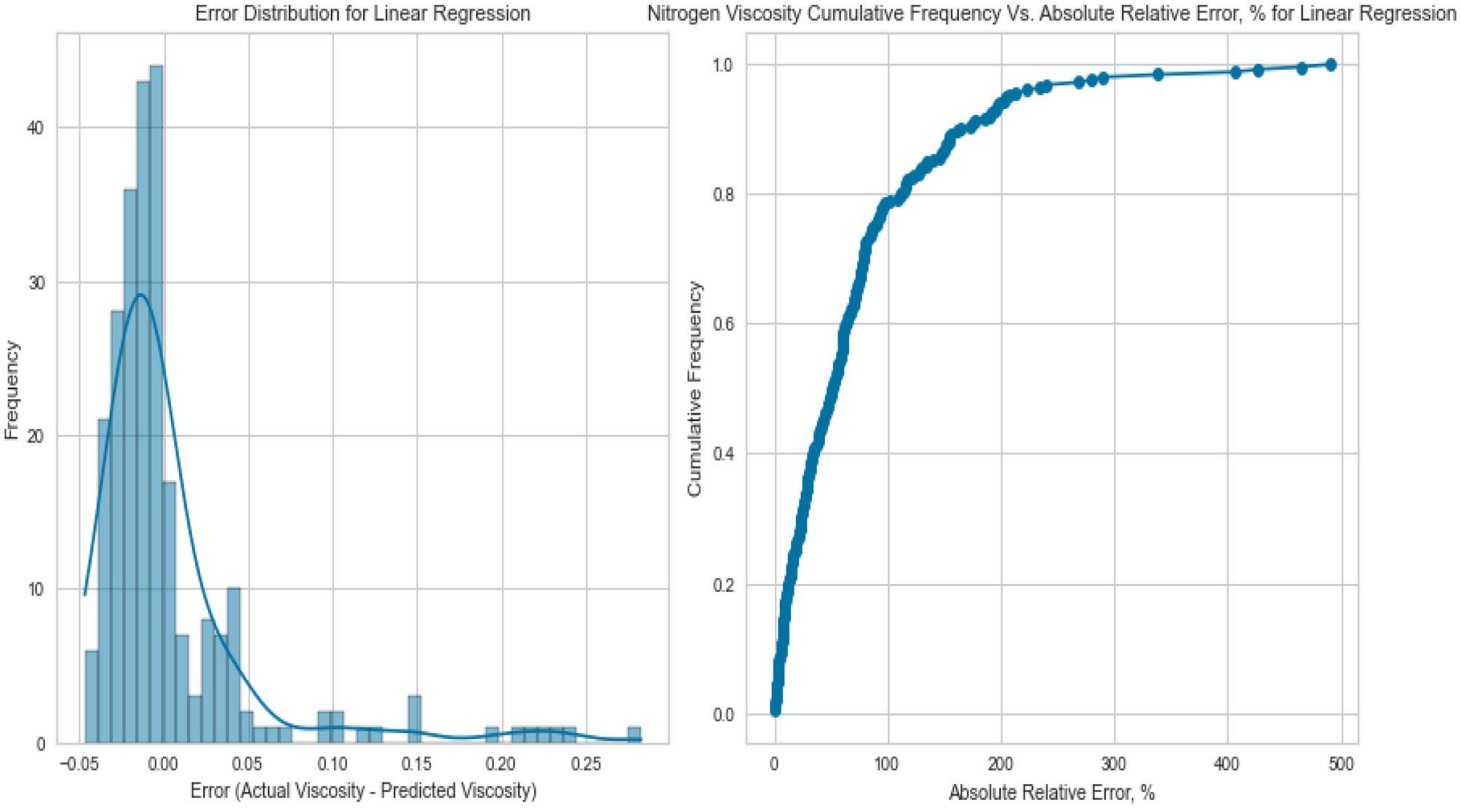
Error distribution with Q-Q plot for linear regression model (nitrogen case).

**Figure 11:**
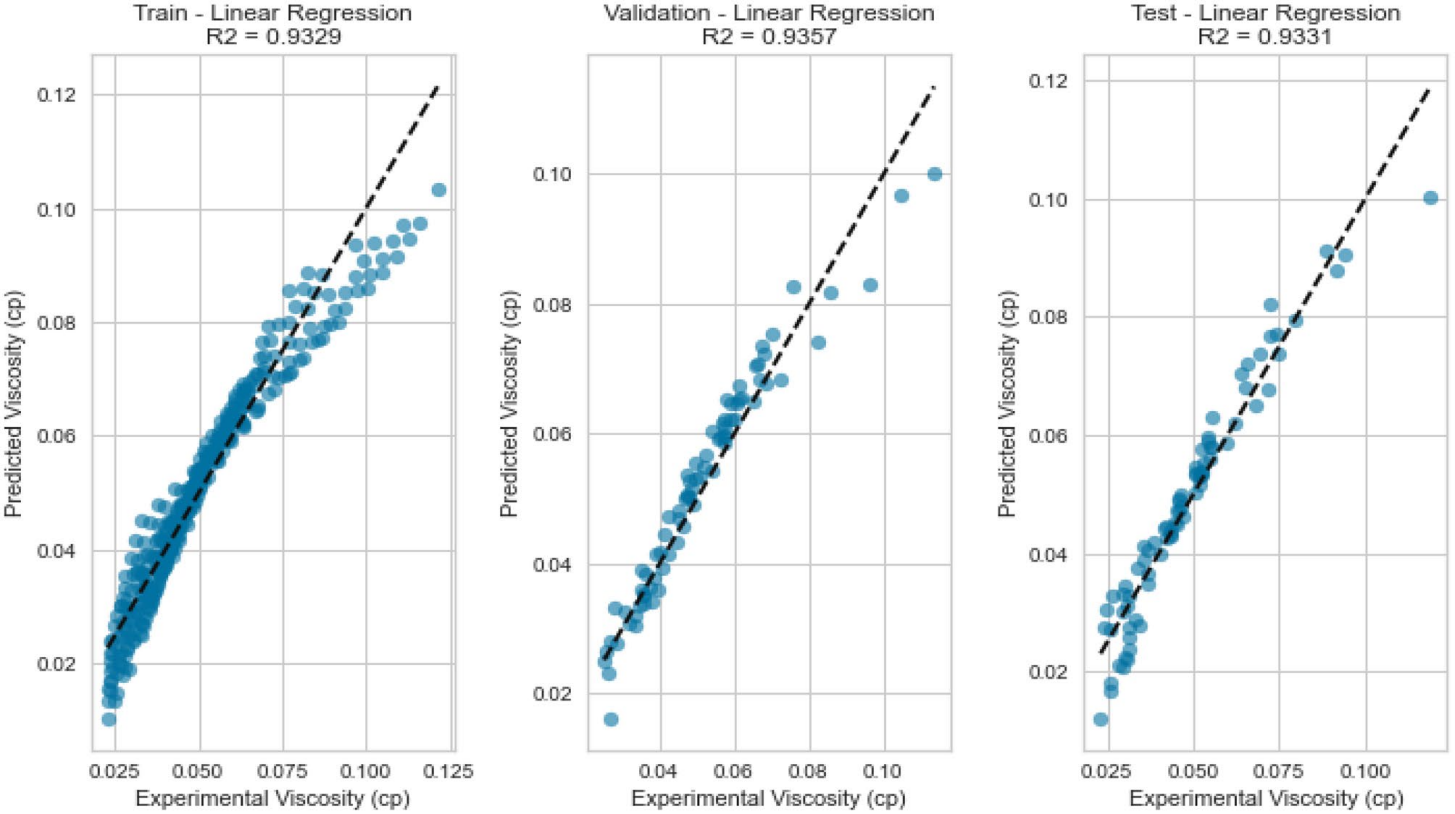
Cross plots of actual versus predicted values for natural gas mixtures viscosity (linear regression model).

**Figure 12:**
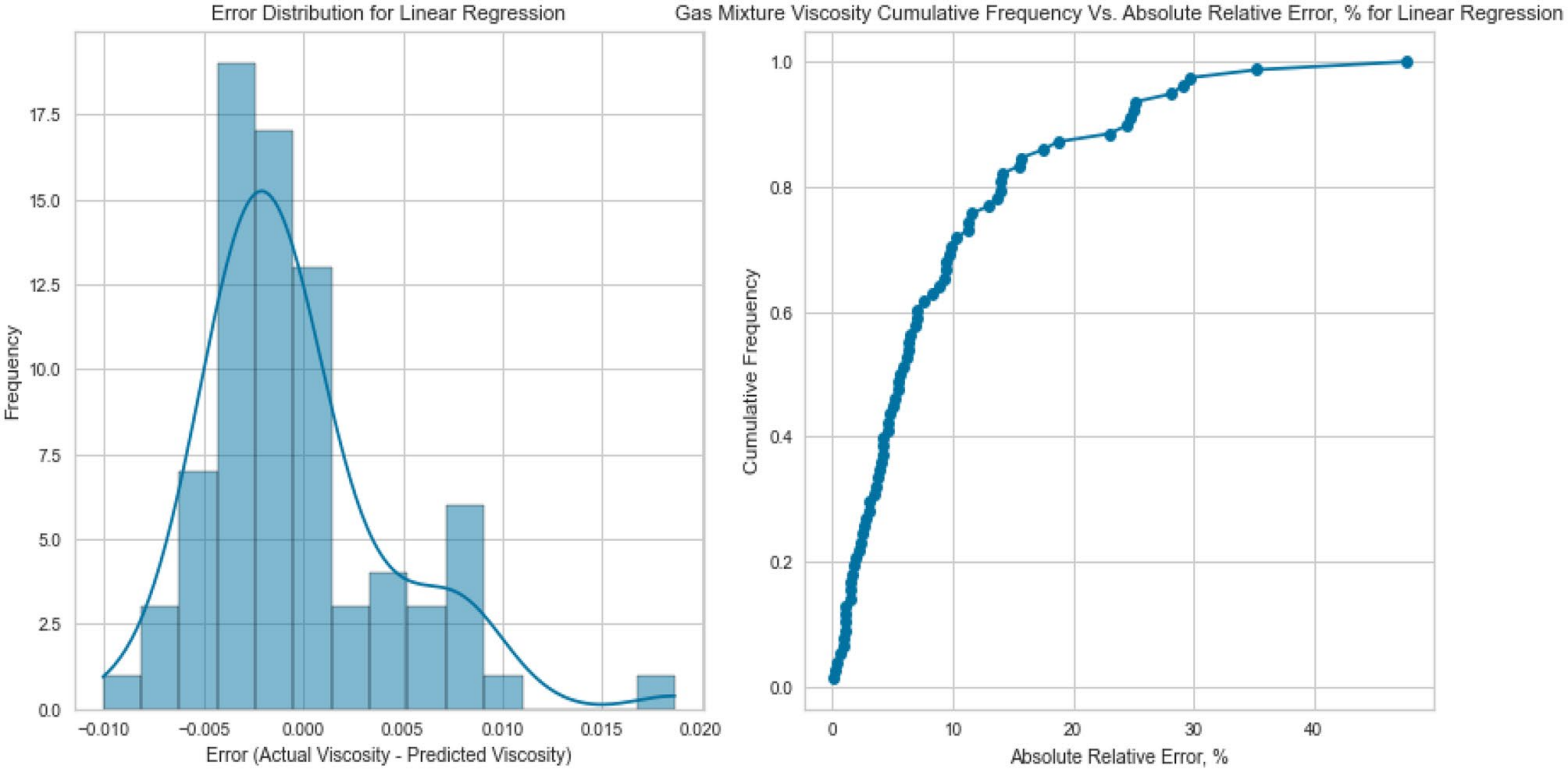
Error distribution with Q-Q plot for linear regression model (natural gas mixtures case).

Linear Regression, a fundamental regression method, was applied to capture the potential linear relationship between the inputs and the target variables. Despite its simplicity, Linear Regression exhibited satisfactory results in predicting gas viscosity as displayed in Figs. 7, 8, 9, 10, 11, and 12, achieving a respectable R^2^ score of 0.9437 and 0.9331 on the testing set for methane and natural gas mixtures respectively. The model’s performance, while commendable, indicates the potential presence of nonlinear relationships that could be better captured by more complex models. However, Linear Regression’s performance varied significantly when applied to Nitrogen gas (Figs. 9 and 10). In this case, the model exhibited a notable drop in performance, achieving a very poor R^2^ score of 0.0736. This discrepancy in model performance suggests that Nitrogen’s viscosity is influenced by factors that Linear Regression, primarily designed for linear relationships, might not effectively capture. The presence of complex nonlinear interactions in the Nitrogen dataset could explain this divergence. Consequently, this underscores the importance of selecting models that are well-suited to the underlying patterns within specific datasets. In the case of Nitrogen, more advanced modeling techniques are required to accurately represent the intricate relationships impacting gas viscosity.

#### Decision tree (DT)

**Figure 13:**
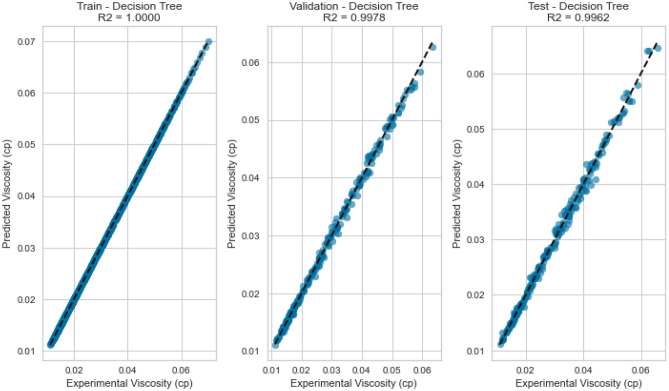
Cross plots of actual versus predicted values for methane viscosity (decision tree model).

**Figure 14:**
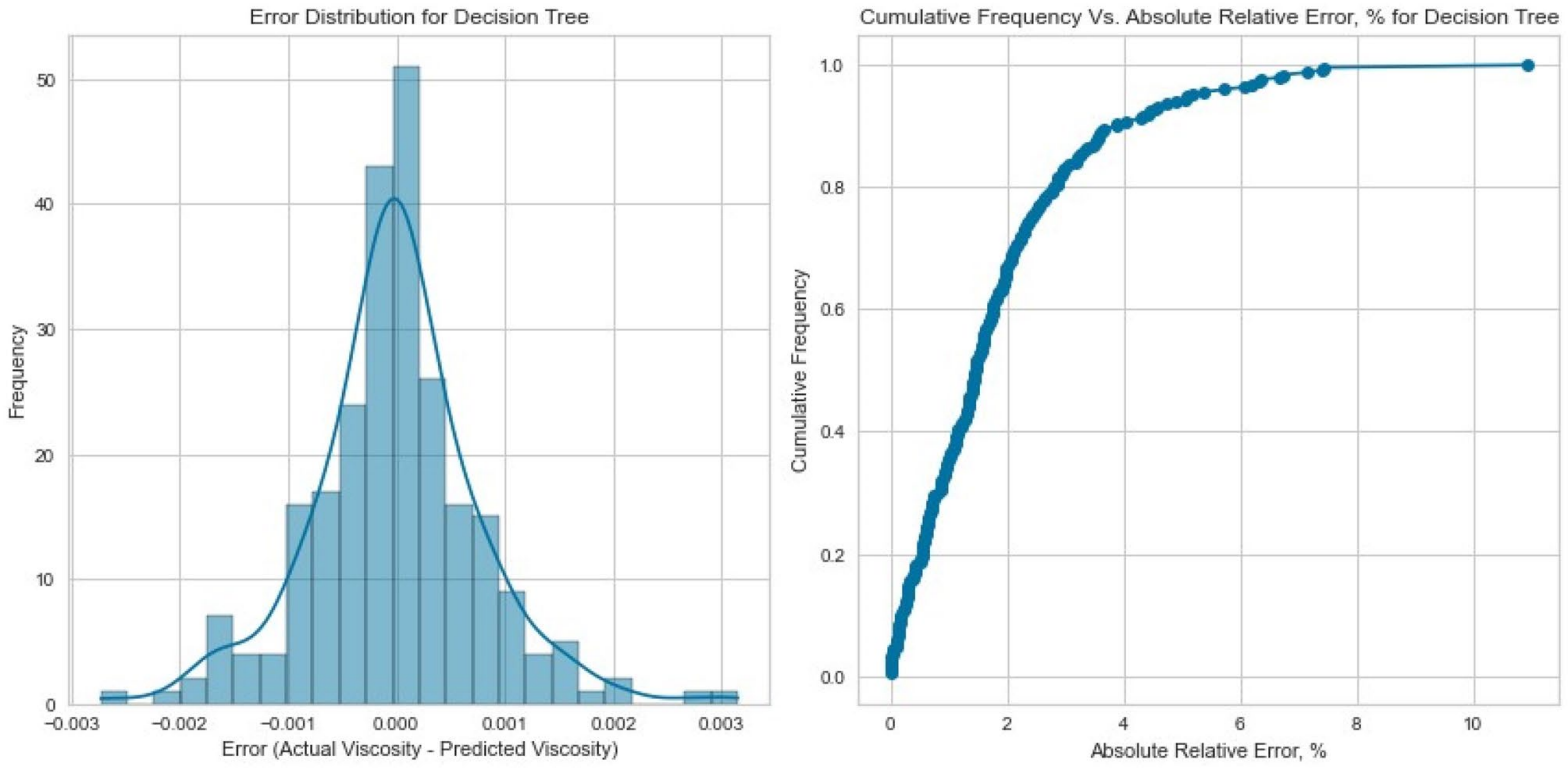
Error distribution for decision tree model (methane case).

**Figure 15:**
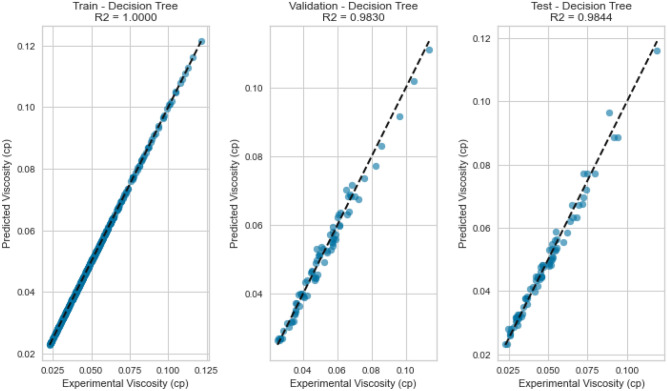
Cross plots of actual versus predicted values for Nitrogen viscosity (decision tree model).

**Figure 16:**
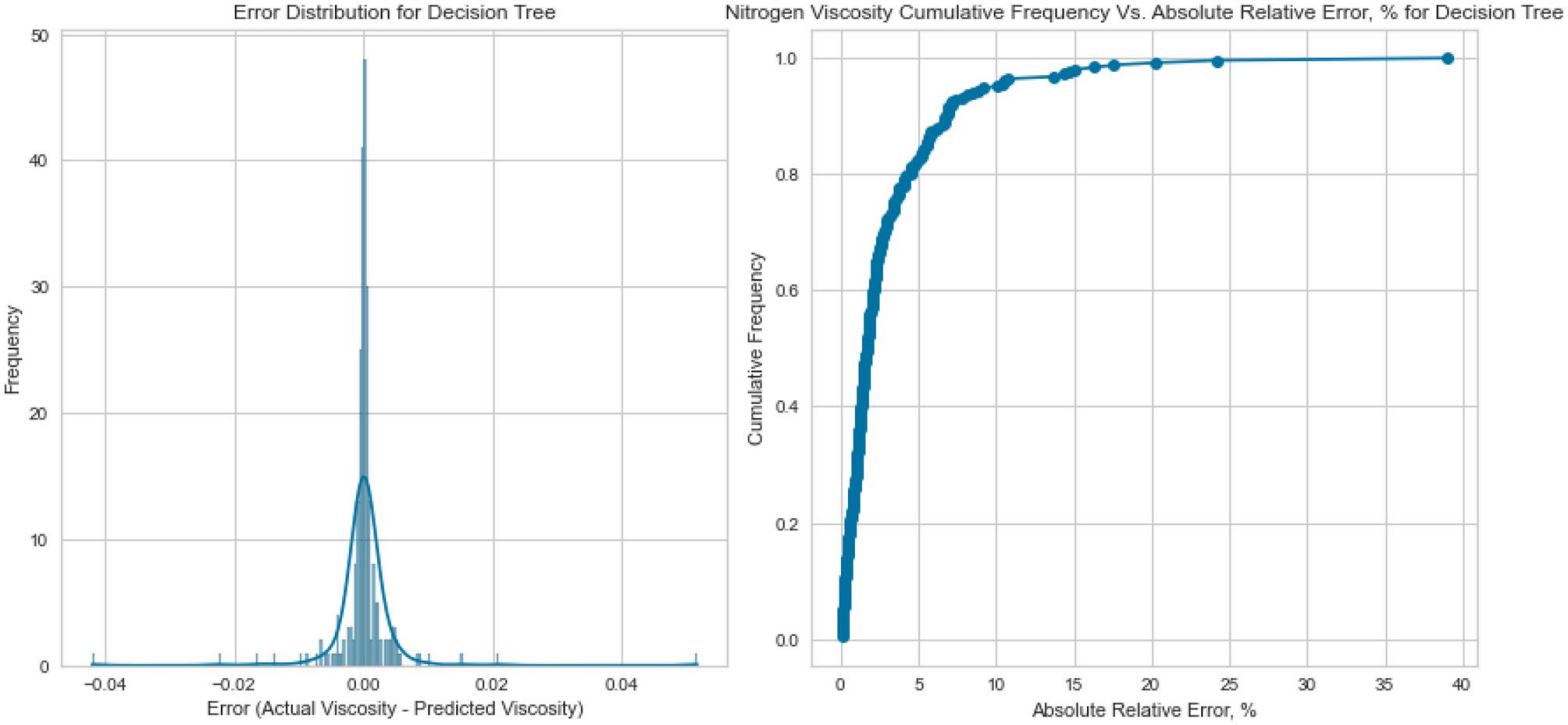
Error distribution for decision tree model (nitrogen case).

**Figure 17:**
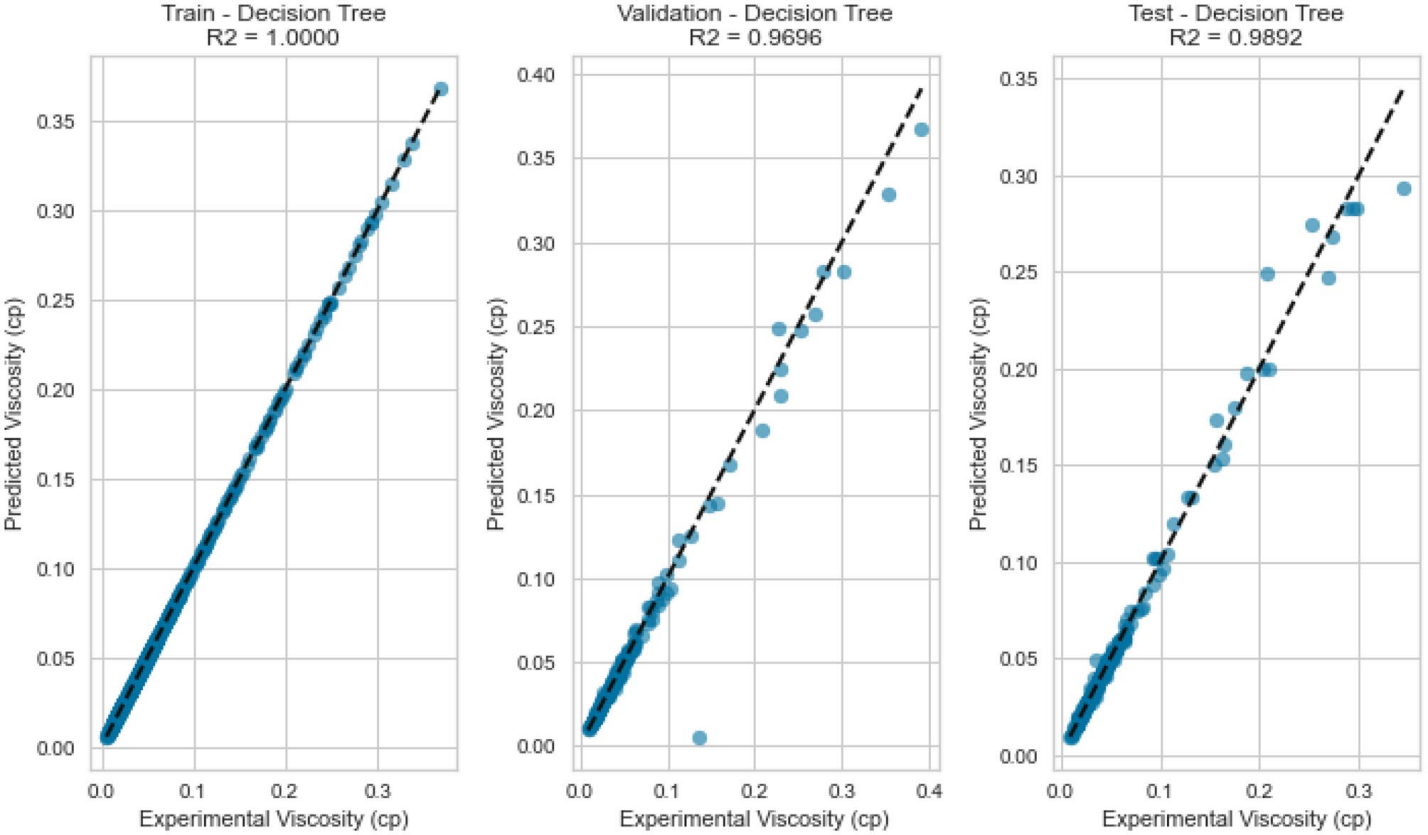
Cross plots of actual versus predicted values for natural gas mixtures viscosity (decision tree model).

**Figure 18:**
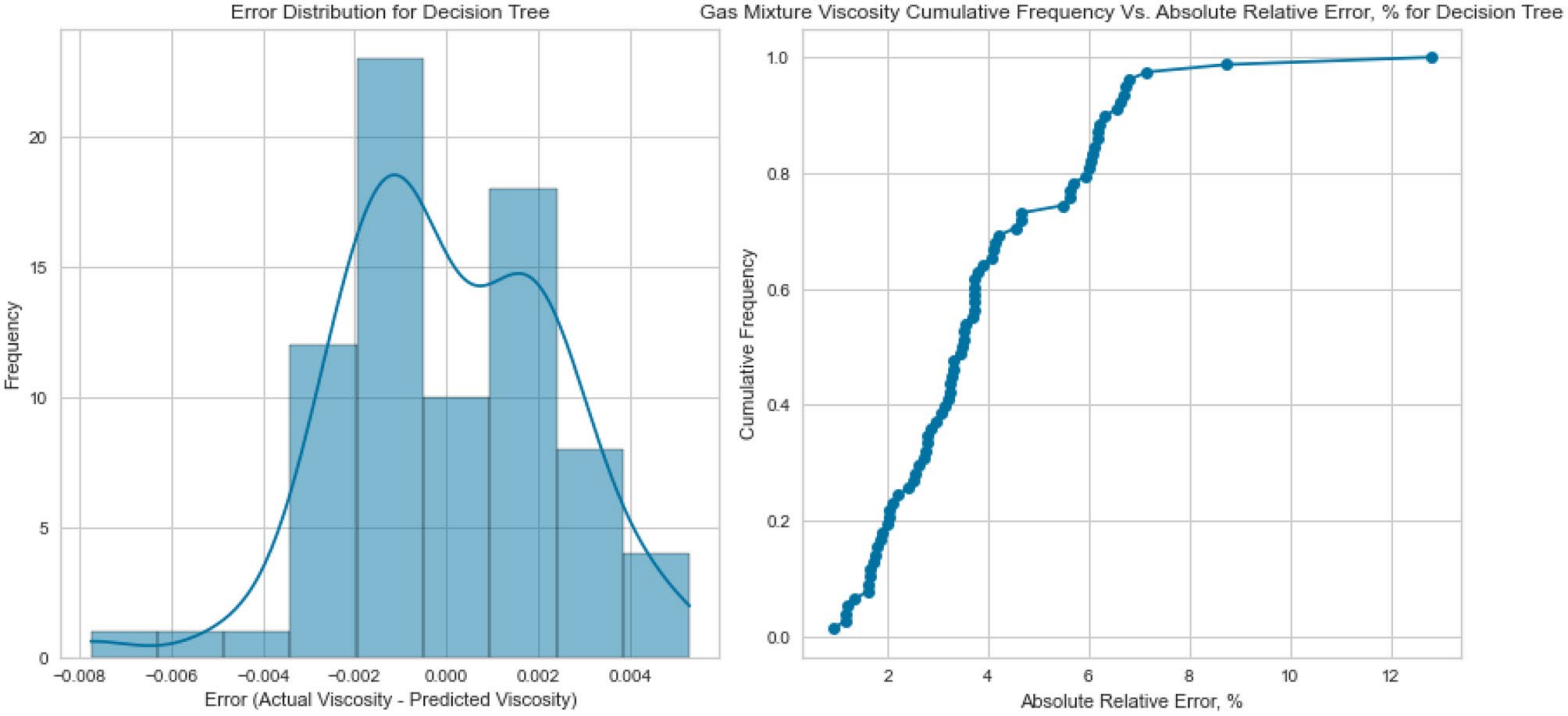
Error distribution for decision tree model (natural gas mixtures case).

Decision Trees offer a powerful framework for capturing intricate interactions within the data. Our Decision Tree model displayed impressive performance on the testing set as displayed in Figs. 13, 14, 15, 16, 17, and 18, achieving an R^2^ score of 0.9962, 0.9844, and 0.9892 for methane, nitrogen, and natural gas mixtures respectively. This suggests that the inherent decision boundaries learned by the model align well with the data’s underlying patterns, allowing it to achieve accurate predictions even for challenging conditions.

#### Random forest

**Figure 19:**
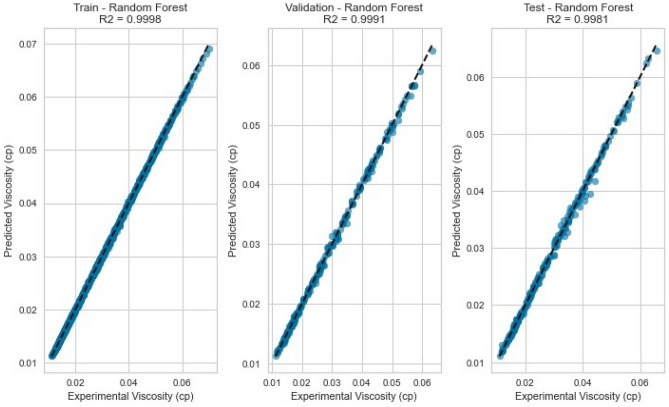
Cross plots of actual versus predicted values for Methane viscosity (random forest model).

**Figure 20:**
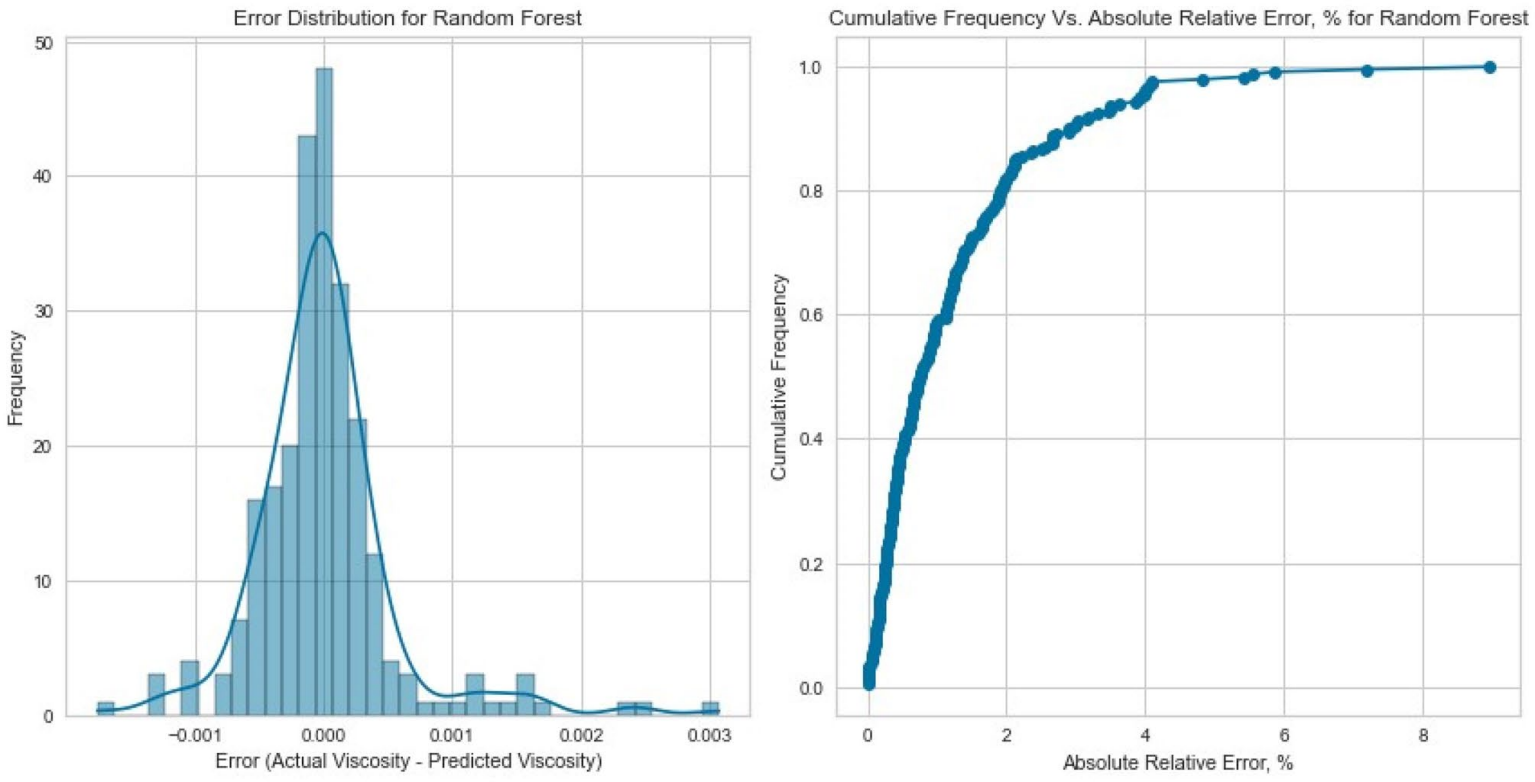
Error distribution for random forest model (methane case).

**Figure 21:**
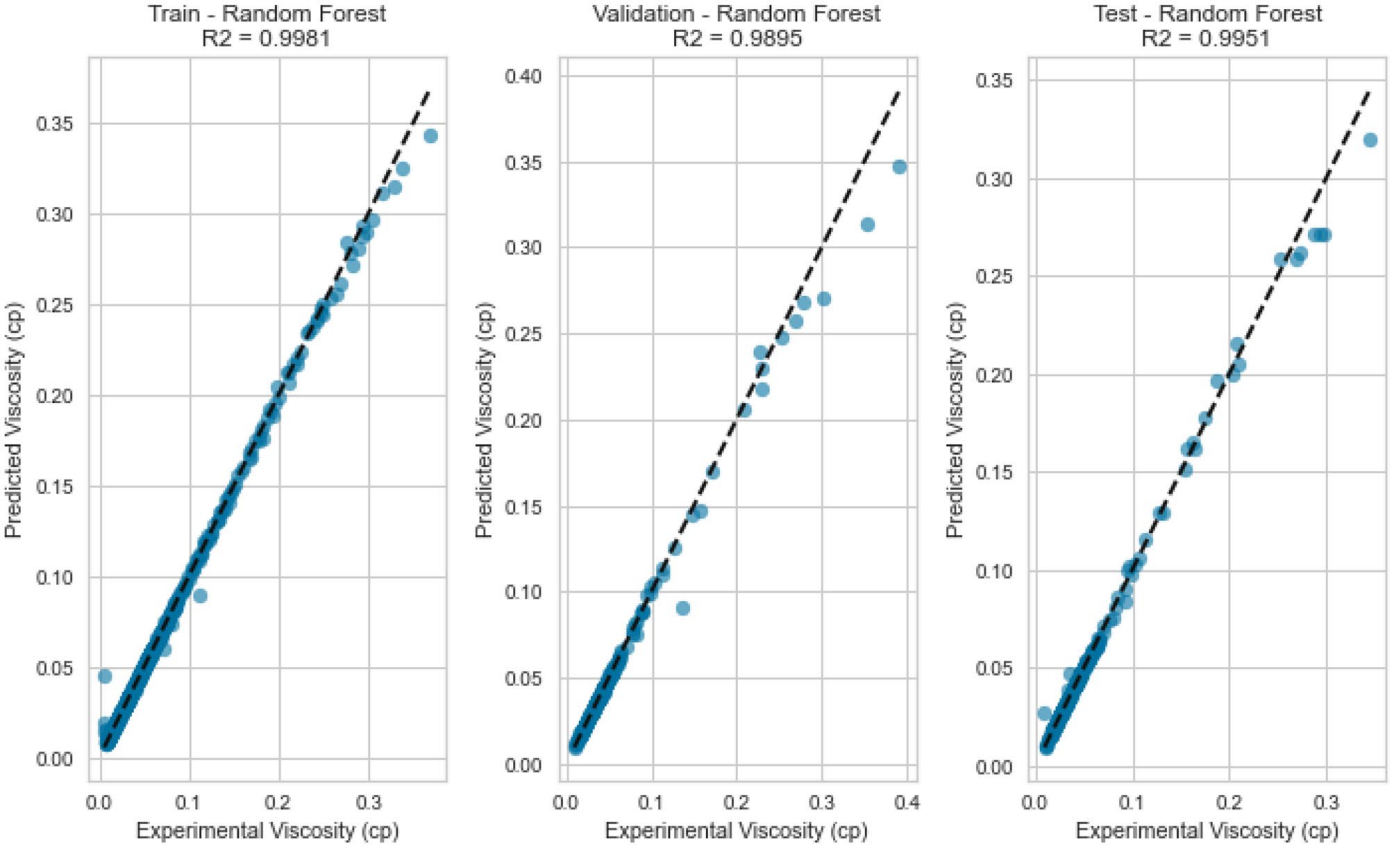
Cross plots of actual versus predicted values for Nitrogen viscosity (random forest model).

**Figure 22:**
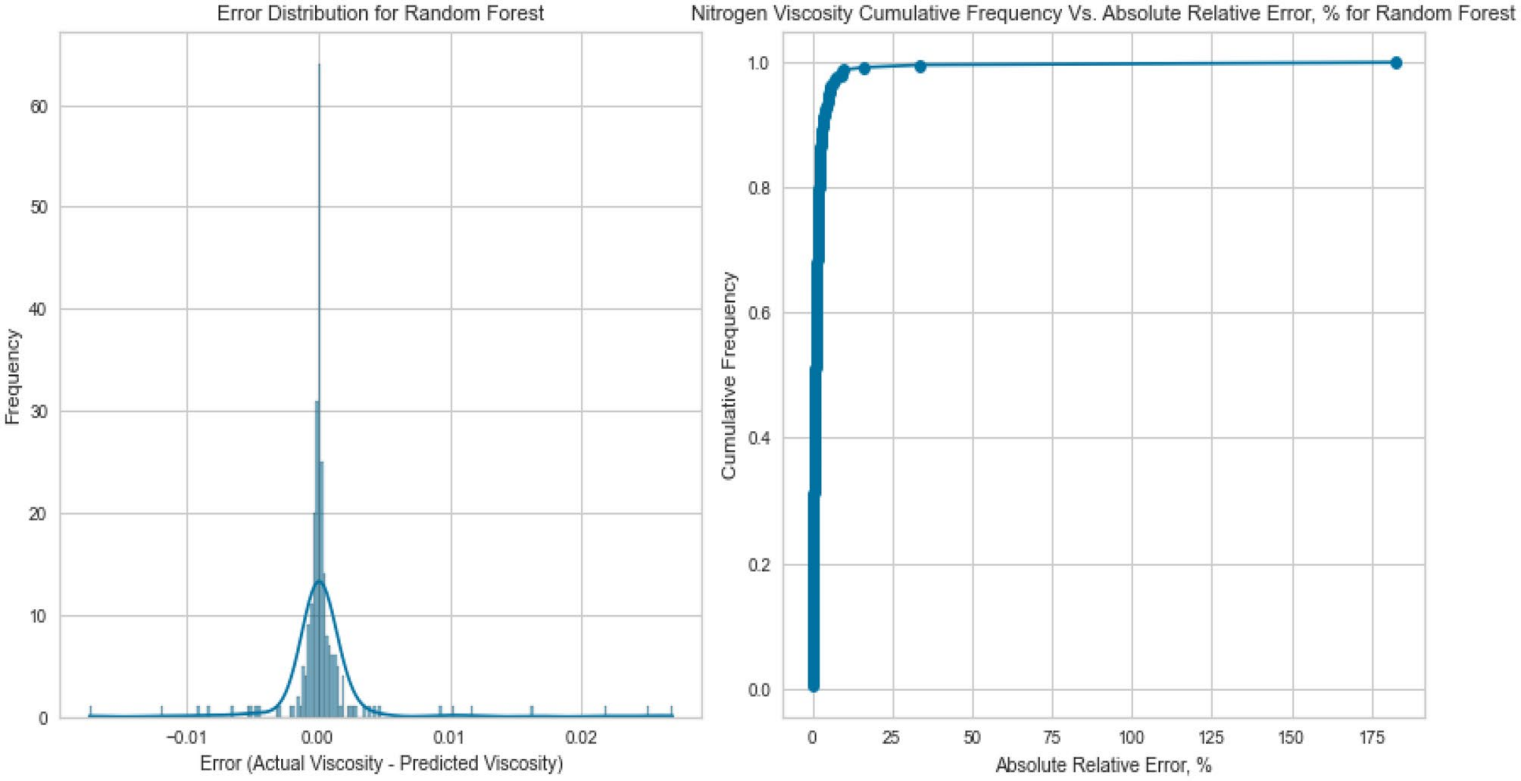
Error distribution for random forest model (nitrogen case).

**Figure 23:**
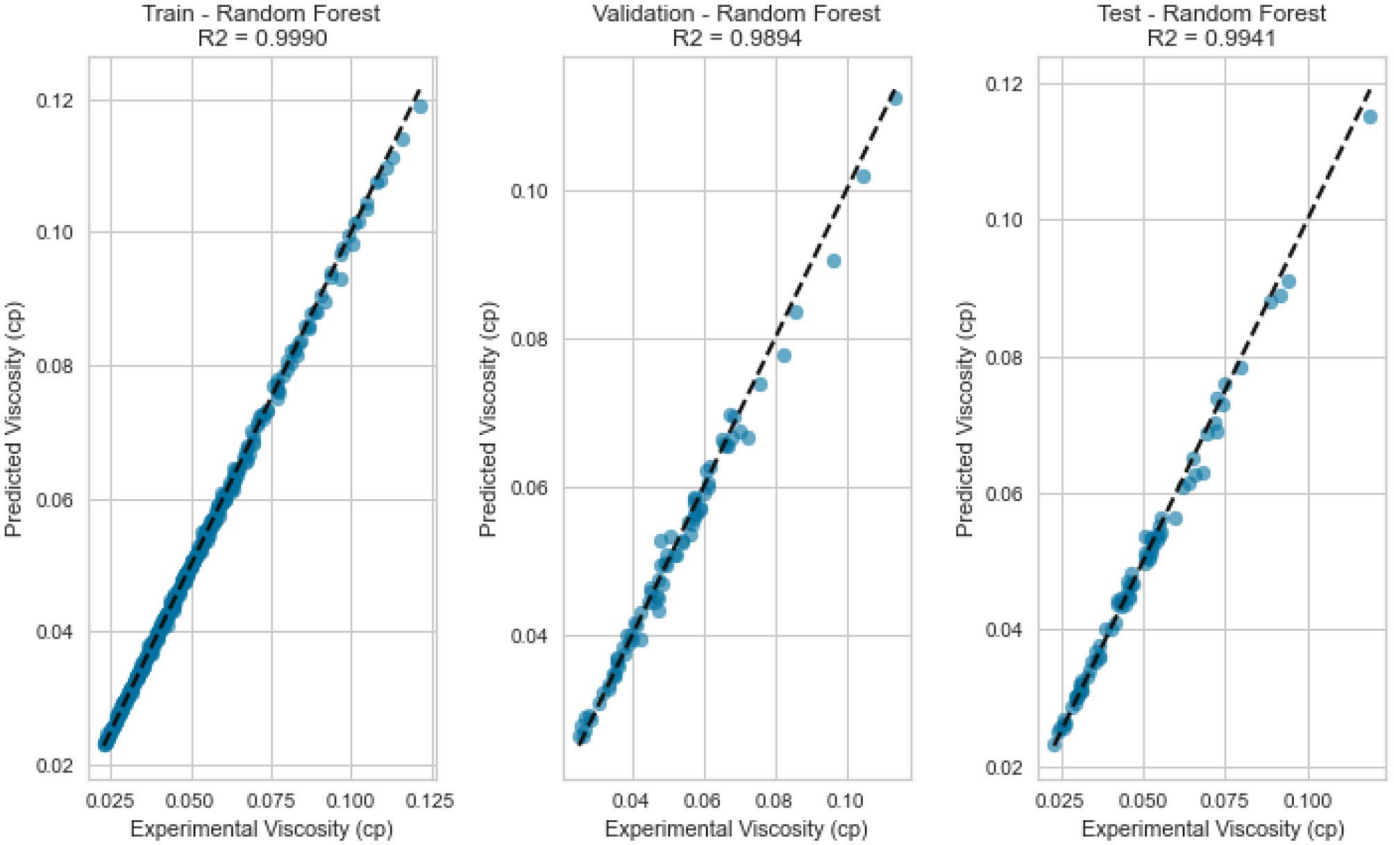
Cross plots of actual versus predicted values for natural gas mixtures viscosity (random forest model).

Ensemble techniques, like Random Forest, aim to alleviate overfitting and enhance predictive precision by gathering multiple decision trees. The Random Forest model delivered exceptional results with an R^2^ score of 0.9981, 0.9951, and 0.9941 for CH_4_, N_2_, and natural gas mixtures respectively on the testing set as displayed in Fig. 19, 20, 21, 22, 23, and 24. The ensemble’s ability to capture diverse feature interactions and provide robust predictions in the presence of noise and outliers contributed to its superior performance.

**Figure 24:**
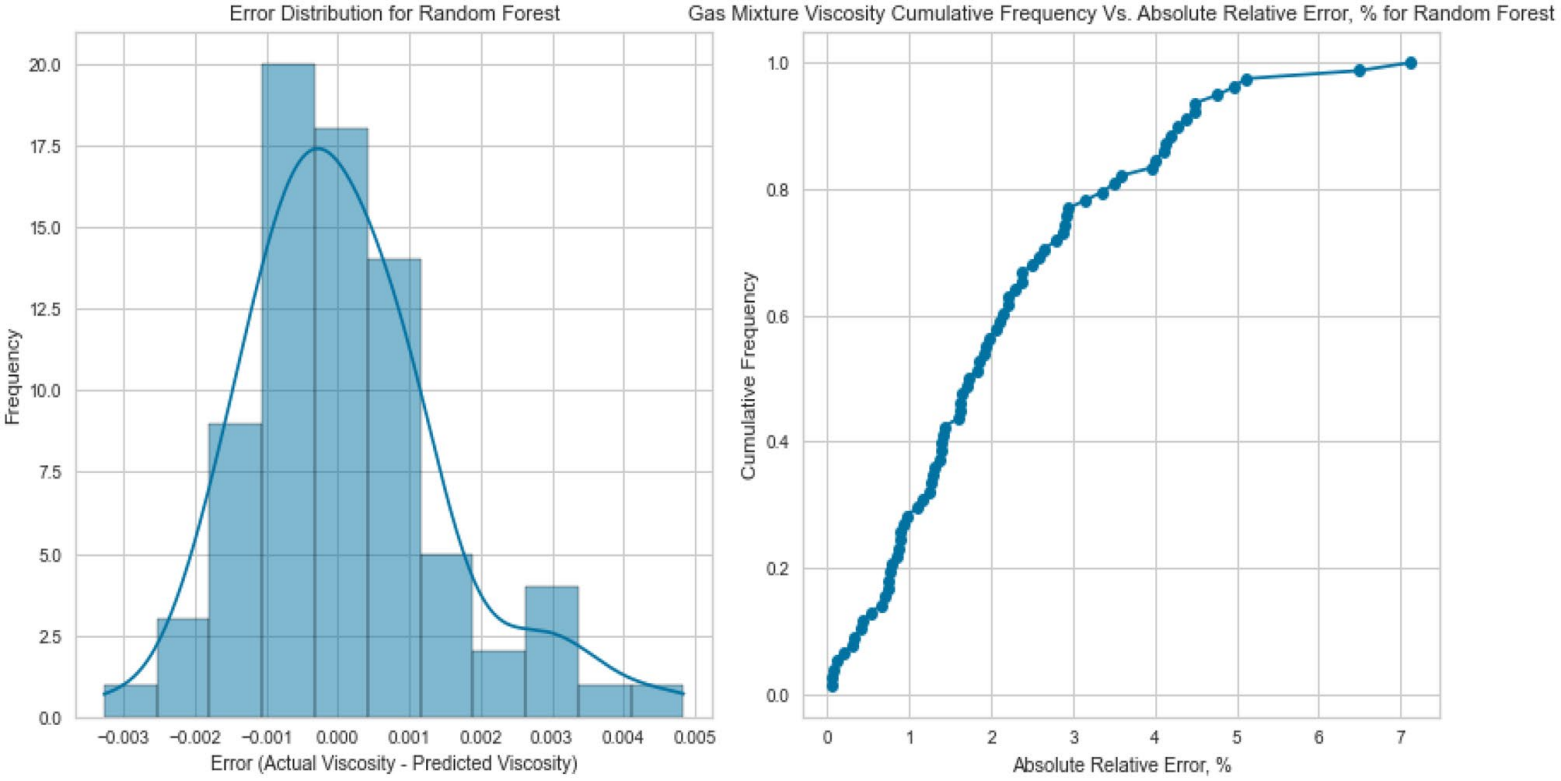
Error distribution for random forest model (natural gas mixtures case).

#### Gradient boosting (GB)

**Figure 25:**
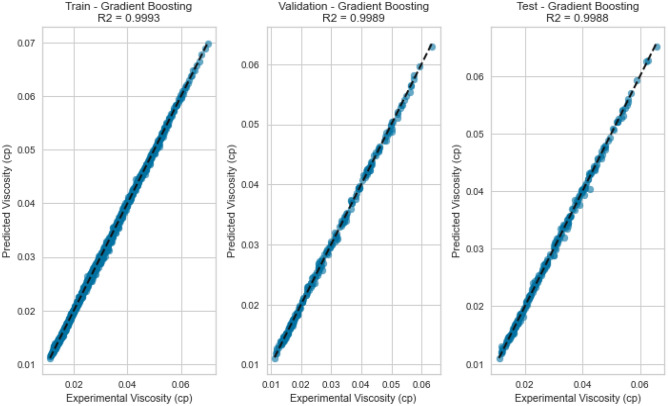
Cross plots of actual versus predicted values for methane viscosity (gradient boosting model).

**Figure 26:**
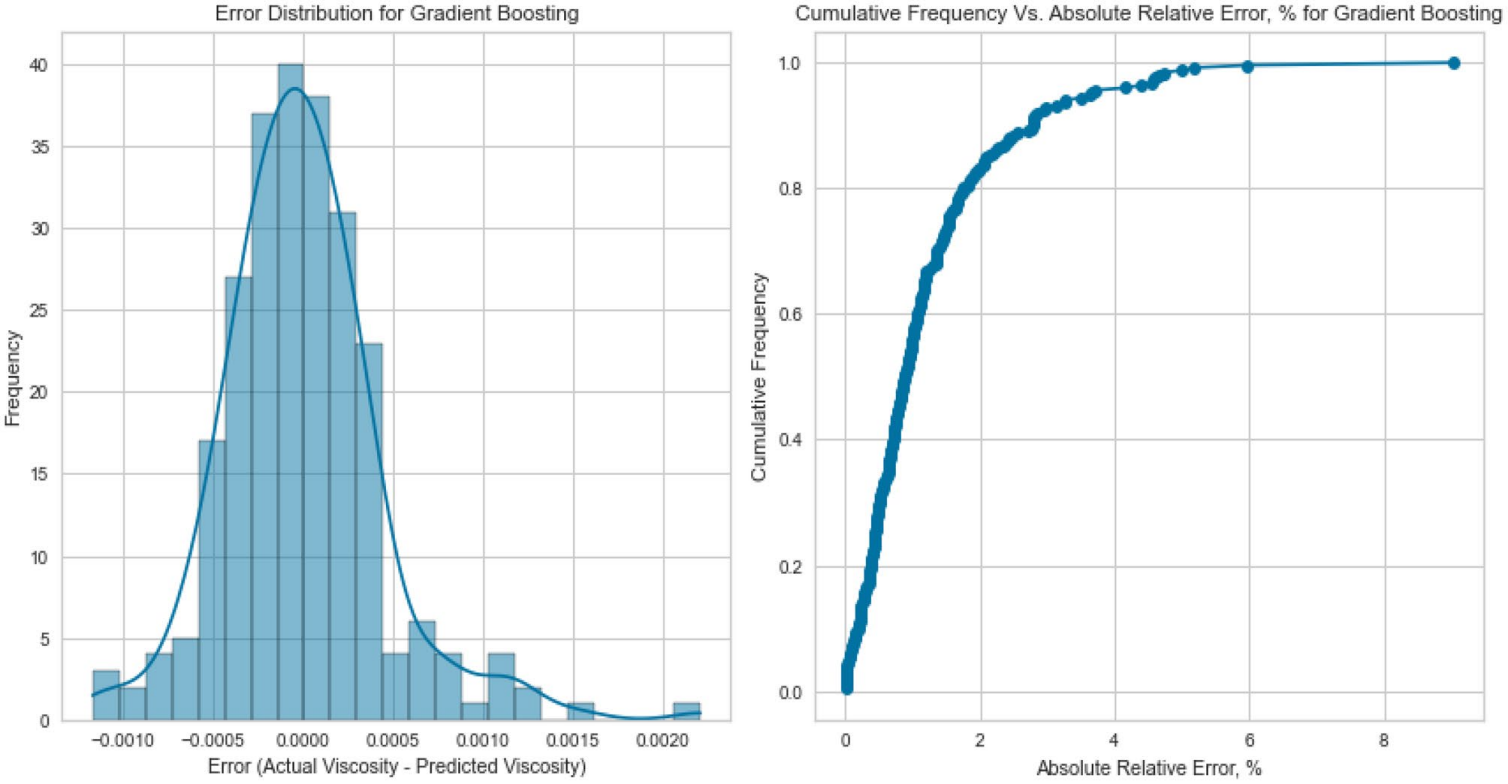
Error distribution for gradient boosting model (methane case).

**Figure 27:**
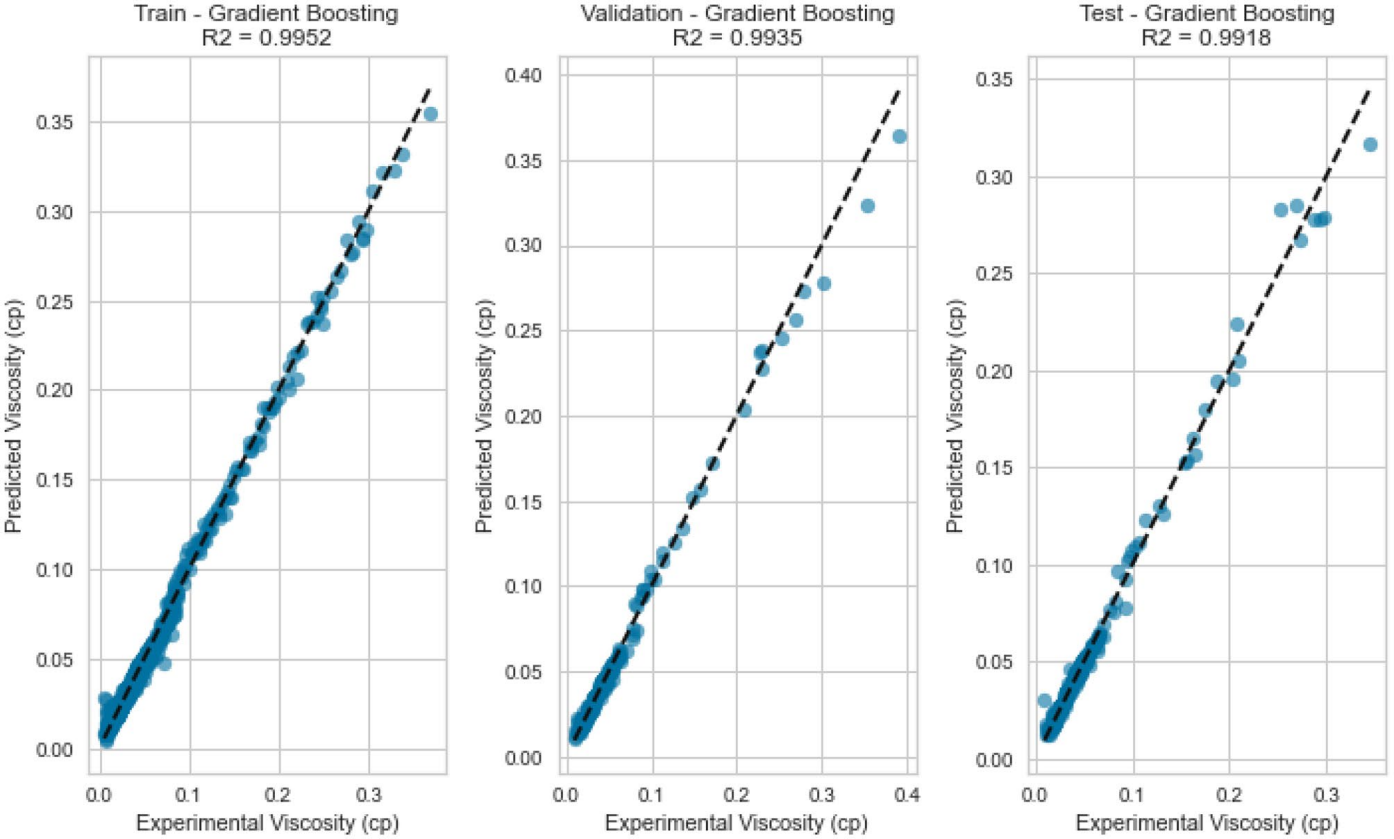
Cross plots of actual versus predicted values for nitrogen viscosity (gradient boosting model).

**Figure 28:**
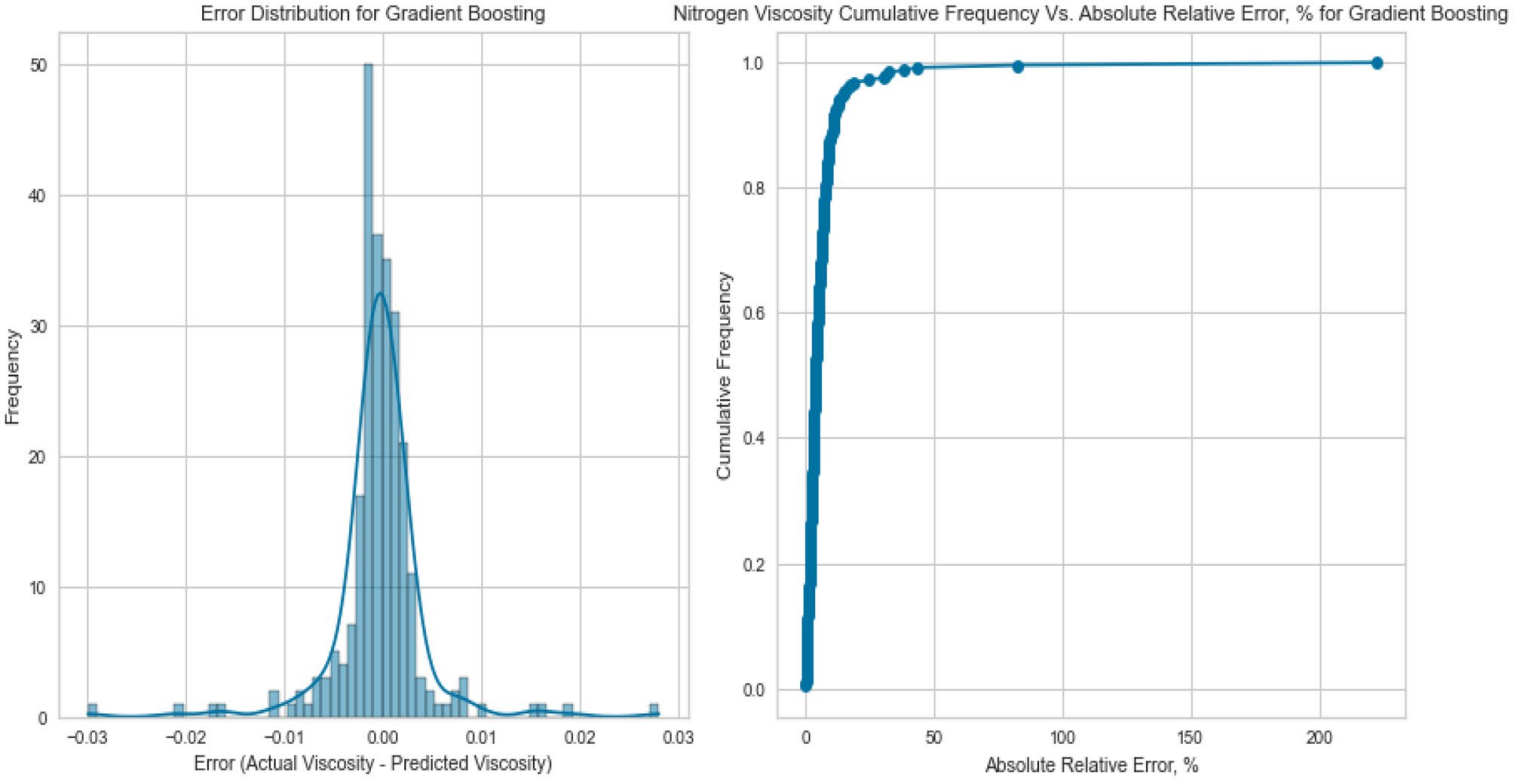
Error distribution for gradient boosting model (nitrogen case).

**Figure 29:**
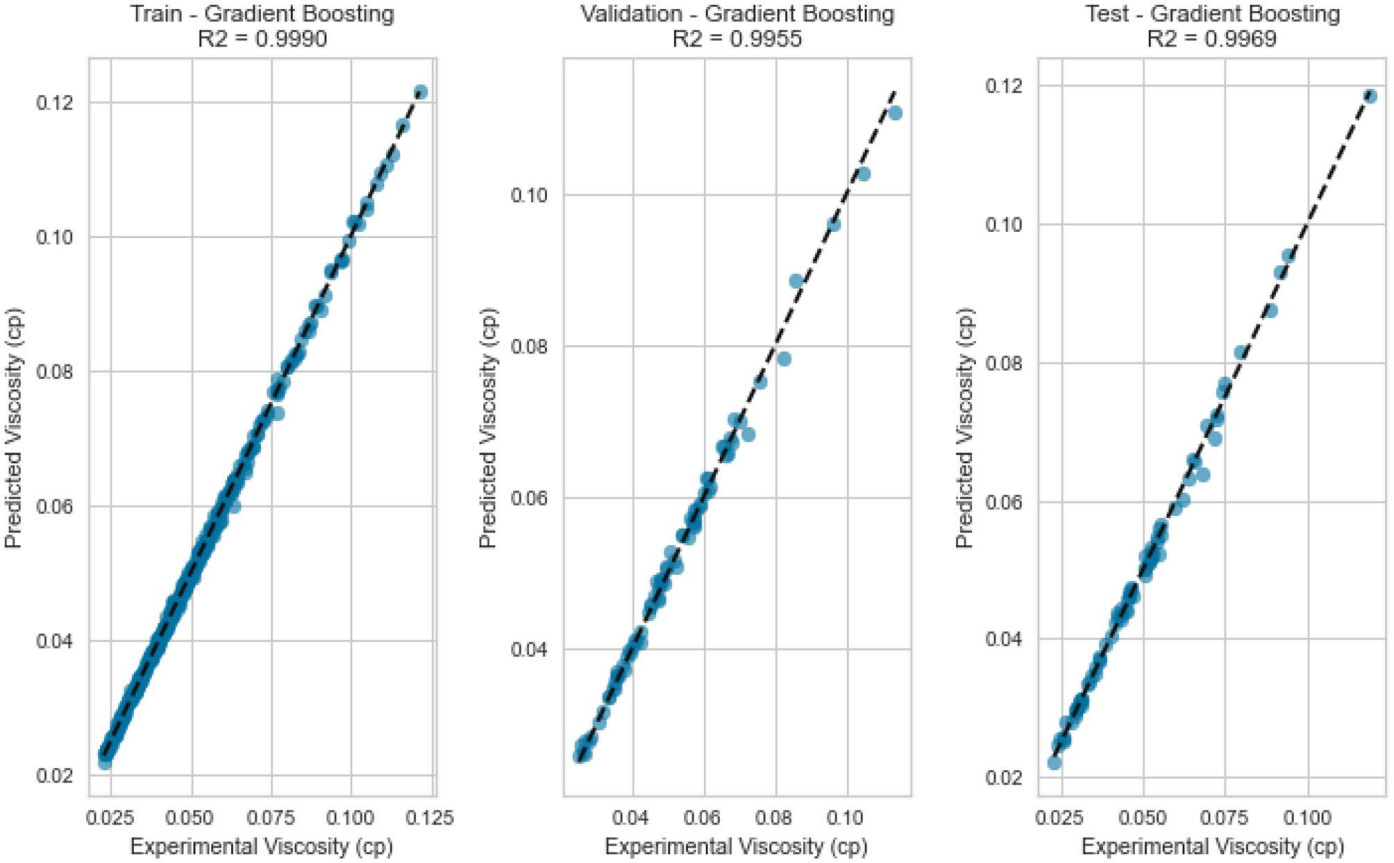
Cross plots of actual versus predicted values for natural gas mixtures viscosity (gradient boosting model).

**Figure 30:**
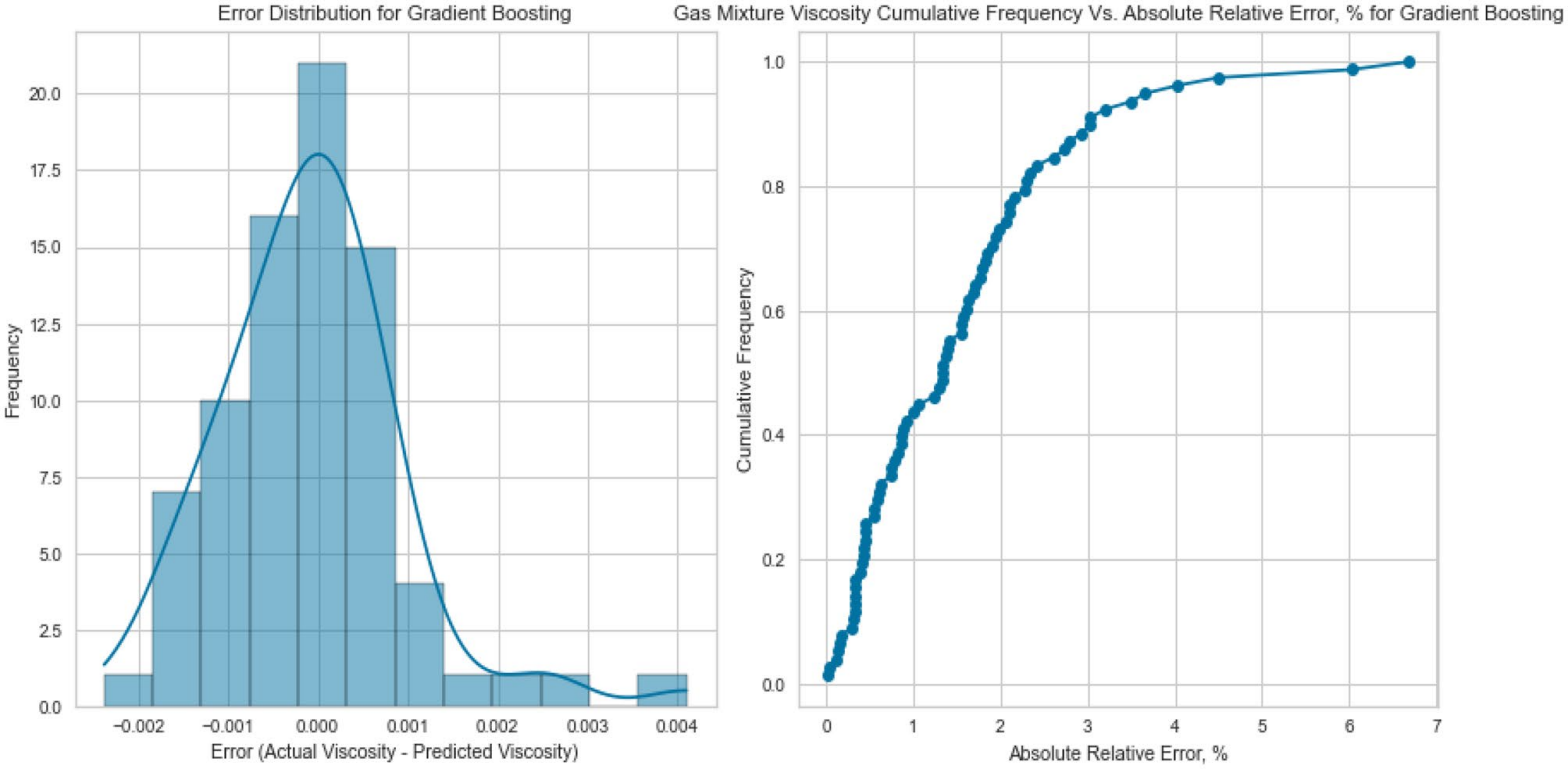
Error distribution for gradient boosting model (natural gas mixtures case).

Gradient Boosting is another sequential ensemble technique that iteratively improves predictions by focusing on samples that the previous iteration misclassified. GB adapts by adjusting subsequent models to correct for errors, culminating in a strong collective predictor. The Gradient Boosting model displayed the best performance, yielding an R^2^ score of 0.9988, 0.9969, and 0.9918 for methane, natural gas mixtures, and nitrogen, respectively on the testing set as displayed in Figs. 25, 26, 27, 28, 29, and 30. The iterative nature of boosting allows the model to focus on challenging instances, enhancing its predictive capabilities. A sequential ensemble technique that iteratively improves predictions by focusing on samples that previous iterations misclassified. GB adapts by adjusting subsequent models to correct for errors, culminating in a strong collective predictor.

#### Nu support vector regression (NuSVR)

**Figure 31:**
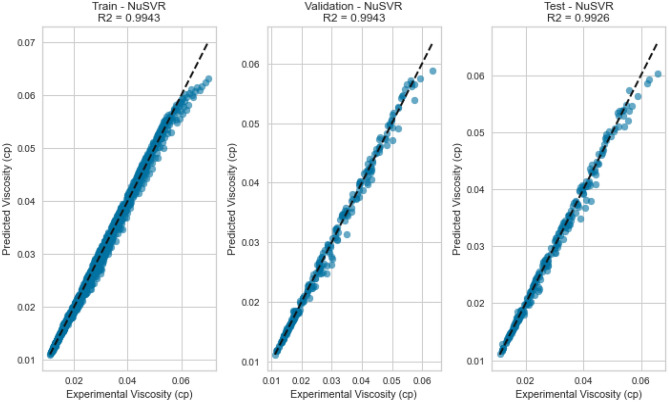
Cross plots of actual versus predicted values for methane viscosity (NuSVR model).

**Figure 32:**
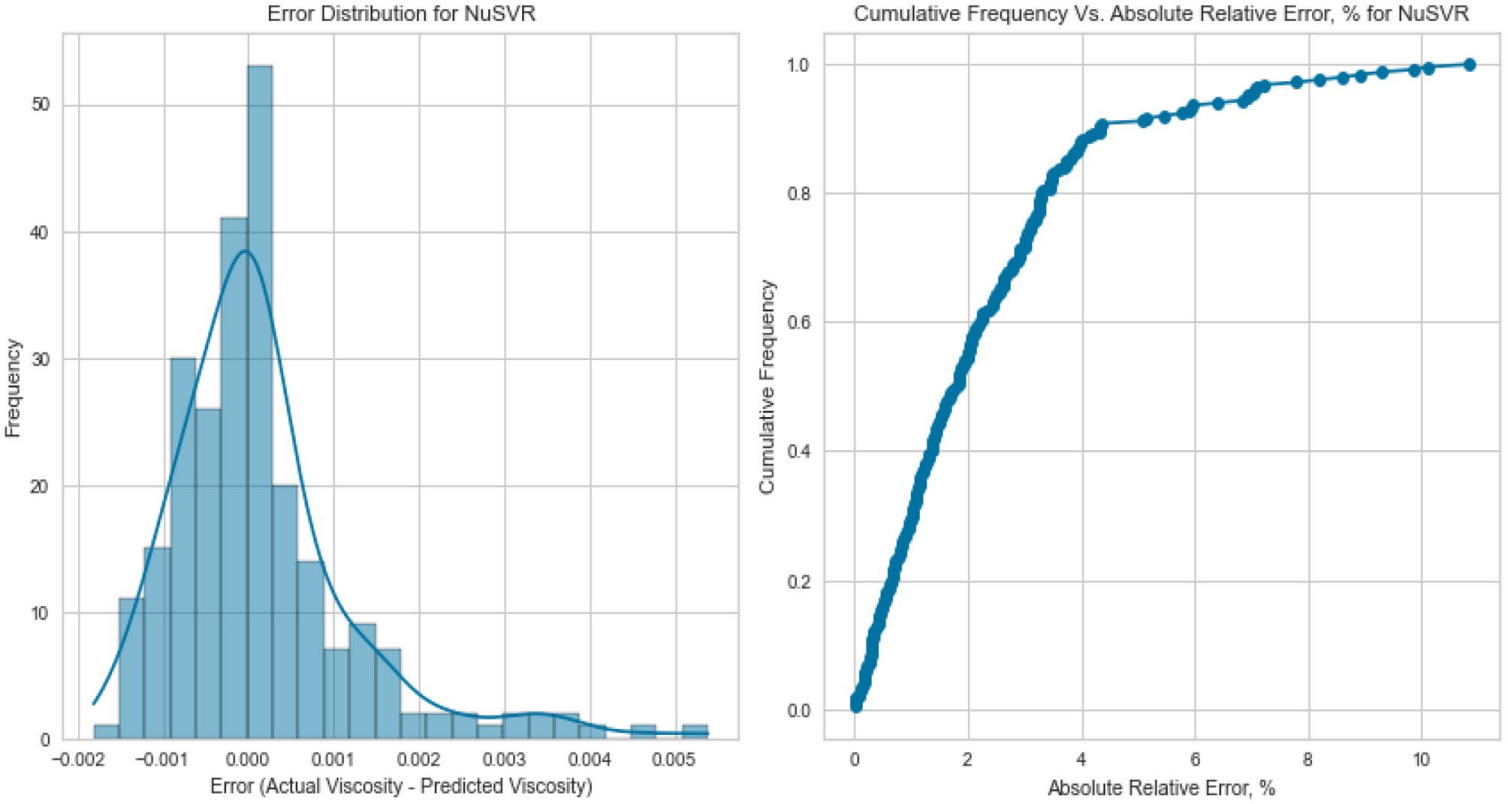
Error distribution for NuSVR model (methane case).

**Figure 33:**
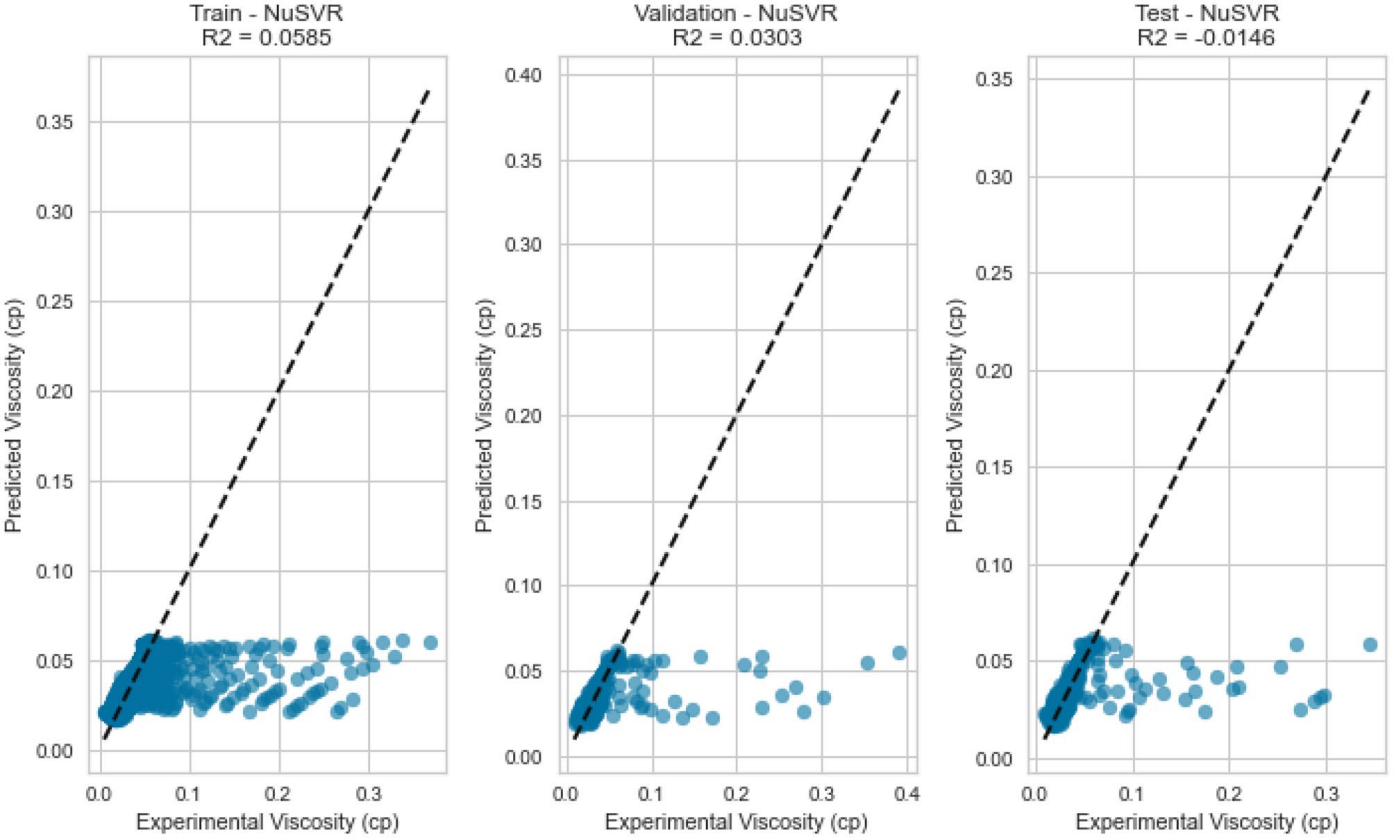
Cross plots of actual versus predicted values for nitrogen viscosity (NuSVR model).

**Figure 34:**
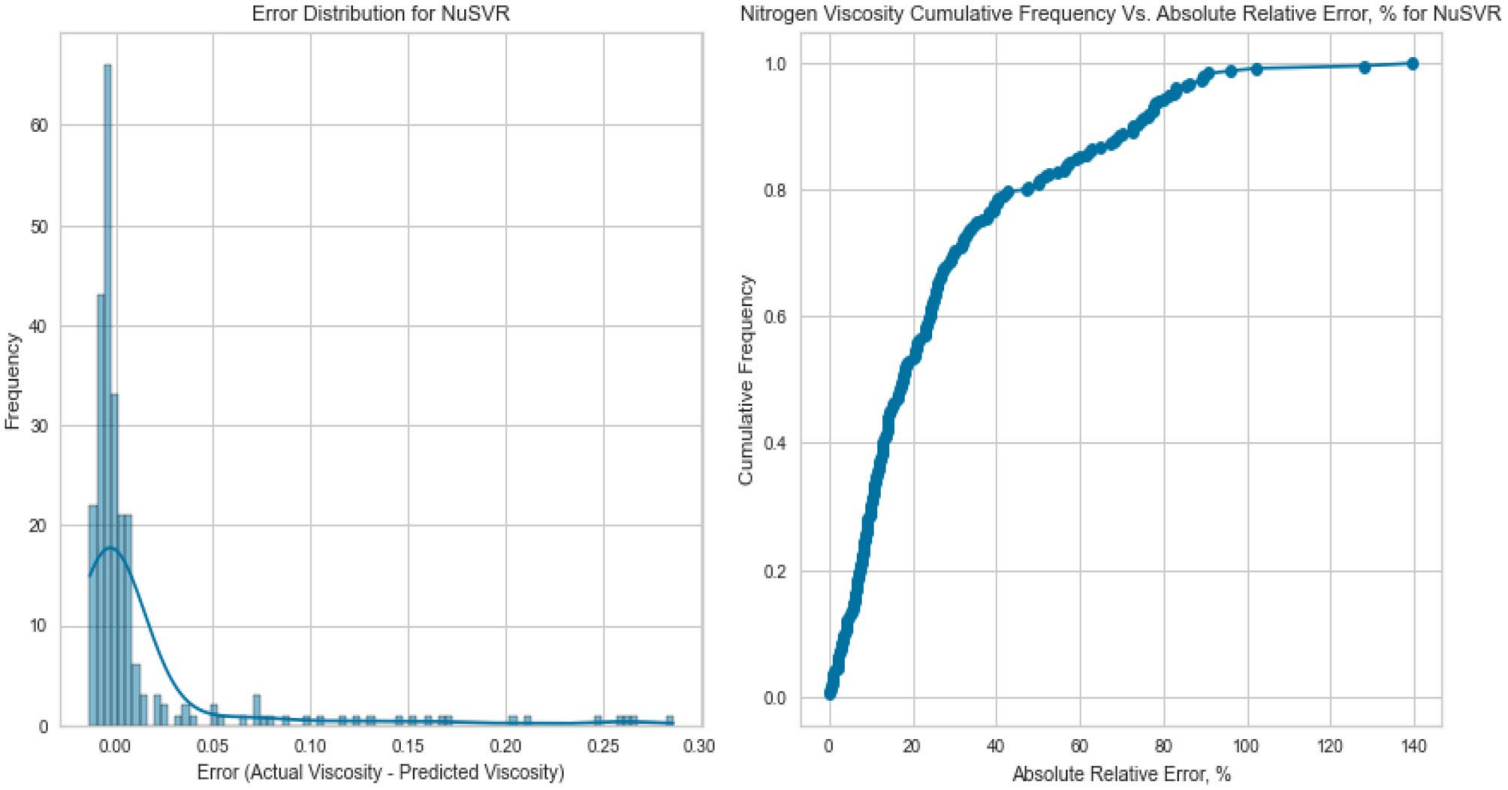
Error distribution for NuSVR model (nitrogen case).

**Figure 35:**
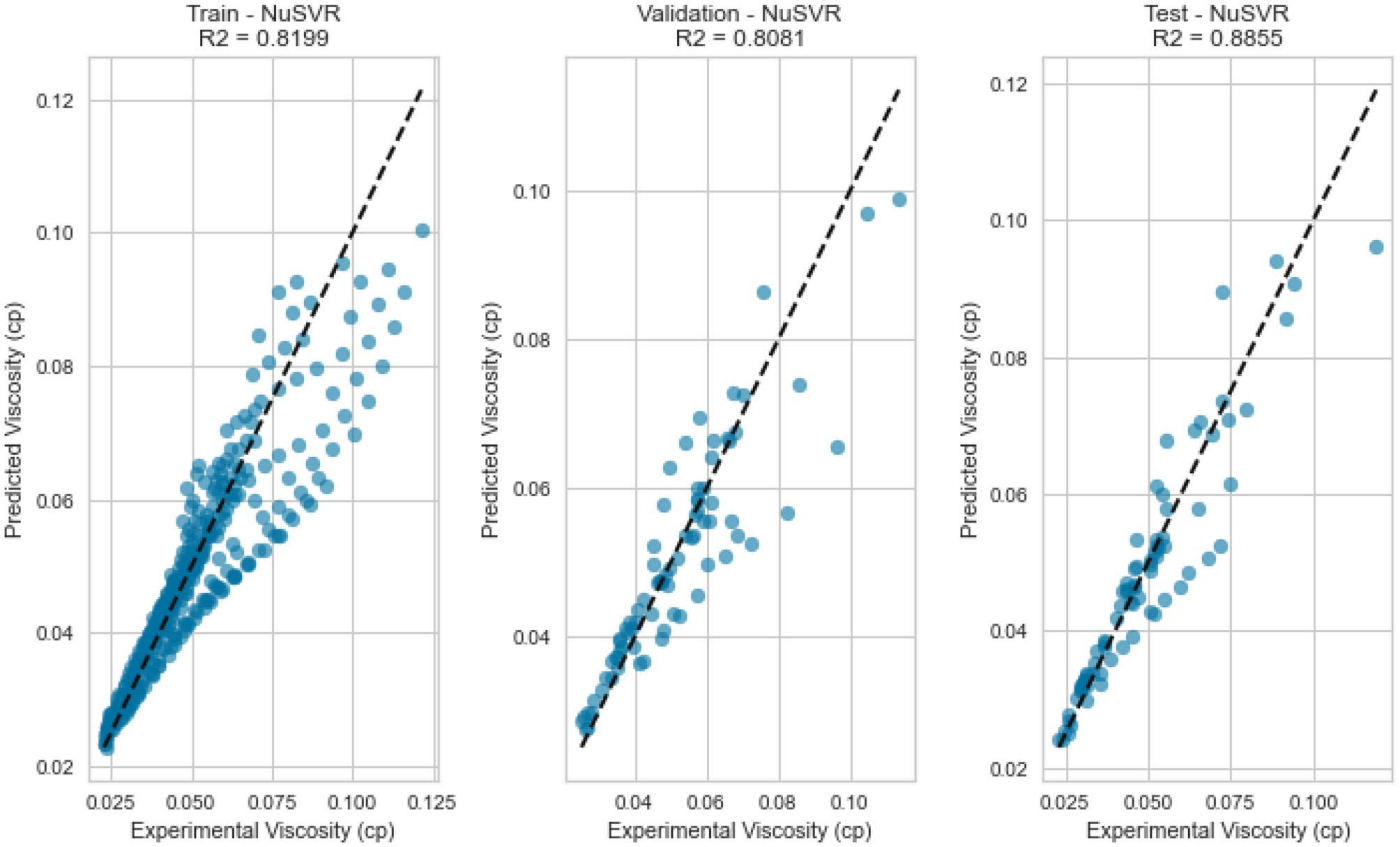
Cross plots of actual versus predicted values for natural gas mixtures viscosity (NuSVR Model).

**Figure 36:**
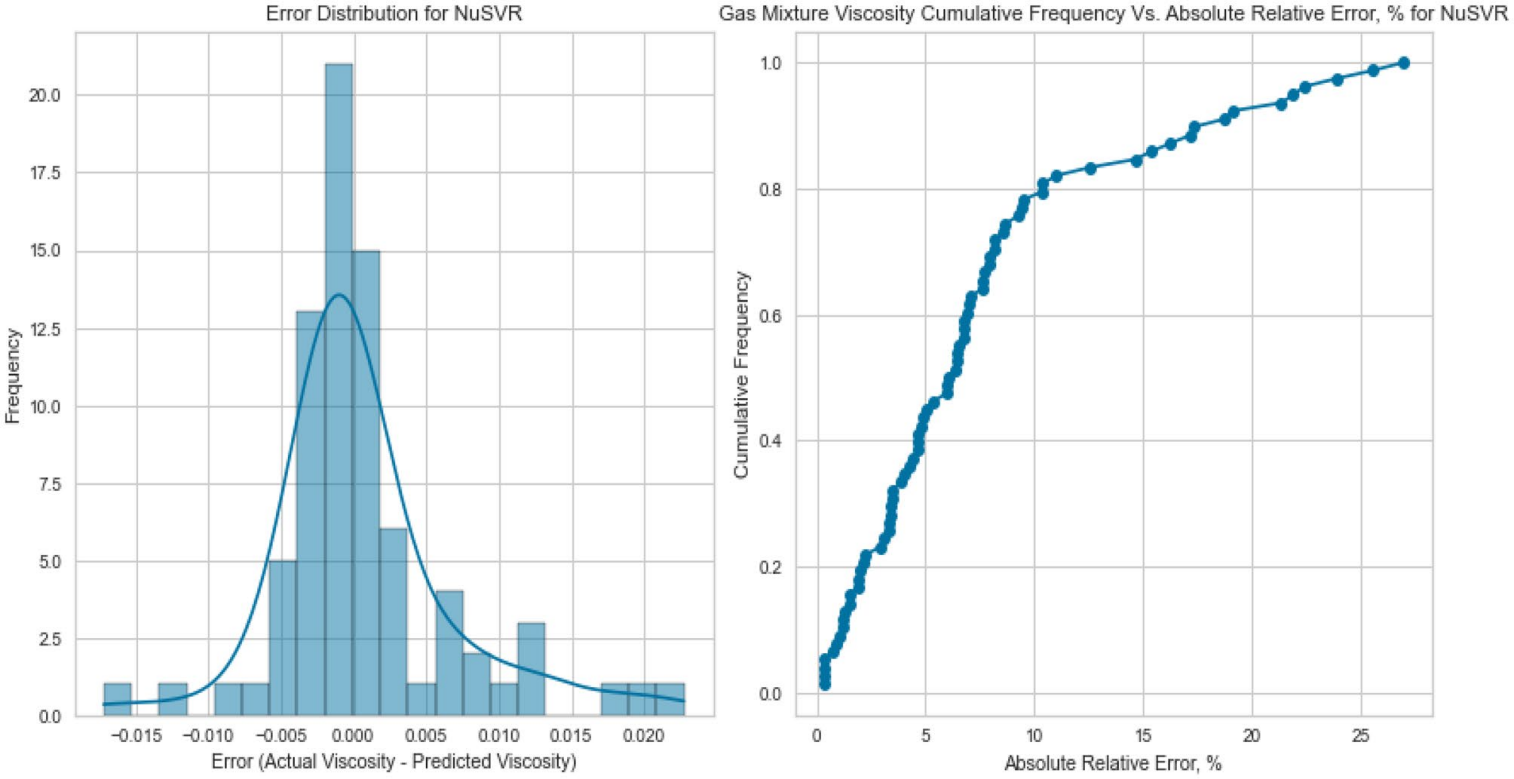
Error distribution for NuSVR model (natural gas mixtures case).

NuSVR, a variant of Support Vector Regression, is adept at capturing complex nonlinear relationships. The NuSVR model achieved a respectable R^2^ score of 0.9926 for methane on the testing set. While not as high as some other models, NuSVR still demonstrated its ability to capture intricate patterns in the data and provide accurate predictions under various conditions. However, the performance of NuSVR did exhibit some degree of variability, most notably evident in its R^2^ scores of 0.8855 for natural gas mixtures and 0.0146 for nitrogen as displayed in Figs. 31, 32, 33, 34, 35, and 36. The relatively high R^2^ score for natural gas mixtures underscores NuSVR’s capacity to grasp the underlying intricacies within the data and make precise predictions. A score of 0.8855 implies that the model accounted for 88.55% of the variance in the natural gas mixtures’ viscosity, indicating a substantial level of accuracy. It is noteworthy that the dataset for natural gas mixtures could involve more predictable patterns or perhaps align more closely with the underlying assumptions of the NuSVR model. Conversely, the notably lower R^2^ score of 0.0146 for nitrogen suggests a diminished ability to capture the complex relationships specific to nitrogen’s viscosity, accounting for only 1.46% of the variance. This discrepancy is indicative of the model’s sensitivity to variations in data patterns and underscores the importance of understanding the specific characteristics of the dataset. In the case of nitrogen, the dataset may comprise more intricate, non-linear relationships that may challenge the model’s predictive capabilities. Overall, NuSVR’s performance emphasizes its adaptability, demonstrating a commendable ability to capture complex data patterns and provide precise predictions. However, the variation in its performance across different datasets underscores the model’s sensitivity to the specific characteristics of the data it encounters. This nuanced understanding can inform future applications of NuSVR in scenarios with distinct data profiles.

#### K-nearest neighbours (KNN)

**Figure 37:**
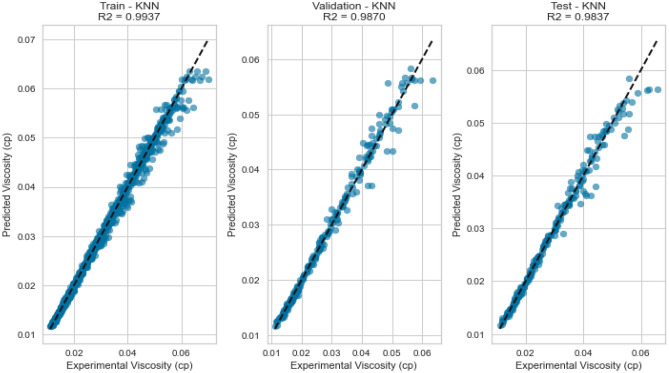
Cross plots of actual versus predicted values for methane viscosity (KNN model).

**Figure 38:**
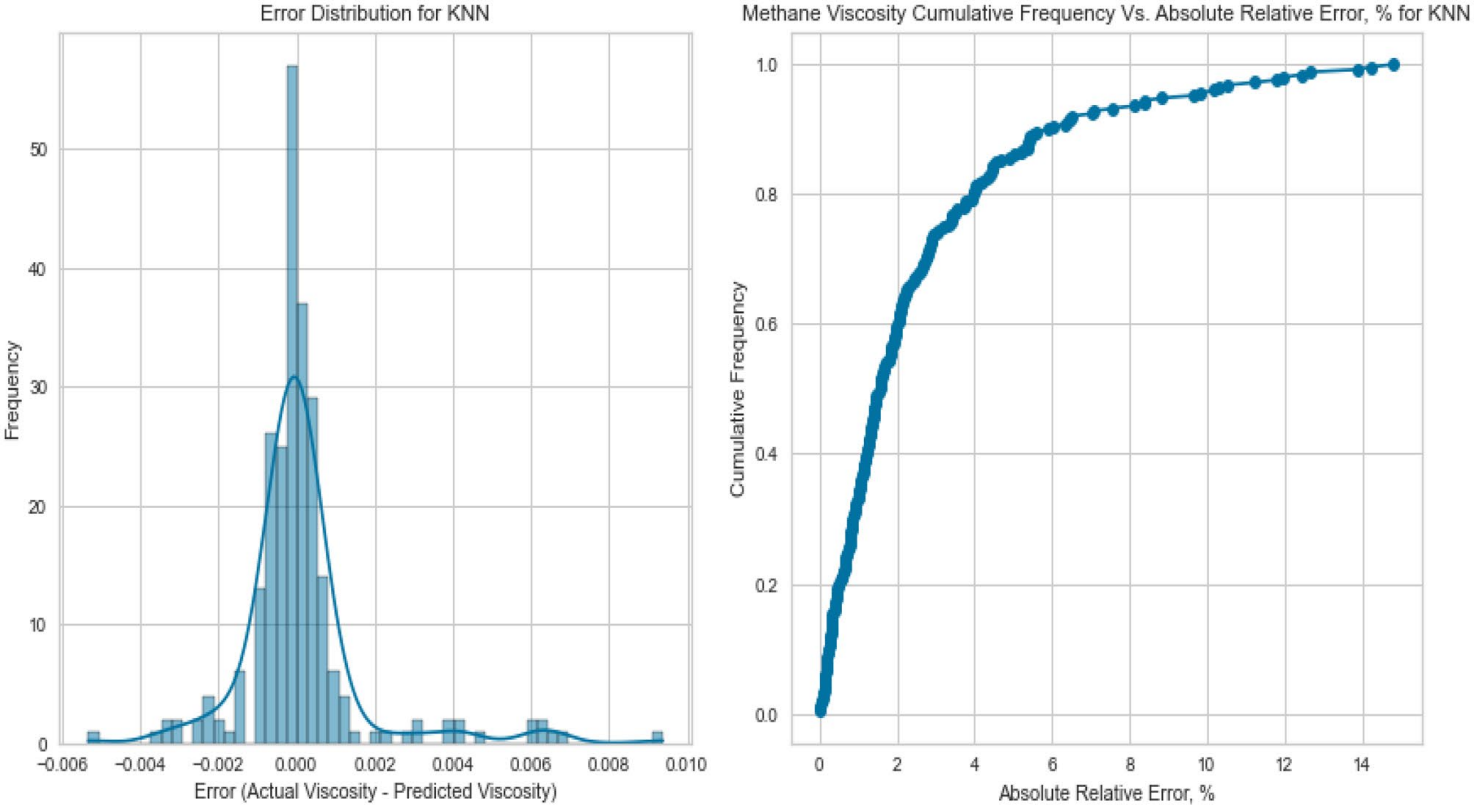
Error distribution for KNN model (methane case).

**Figure 39:**
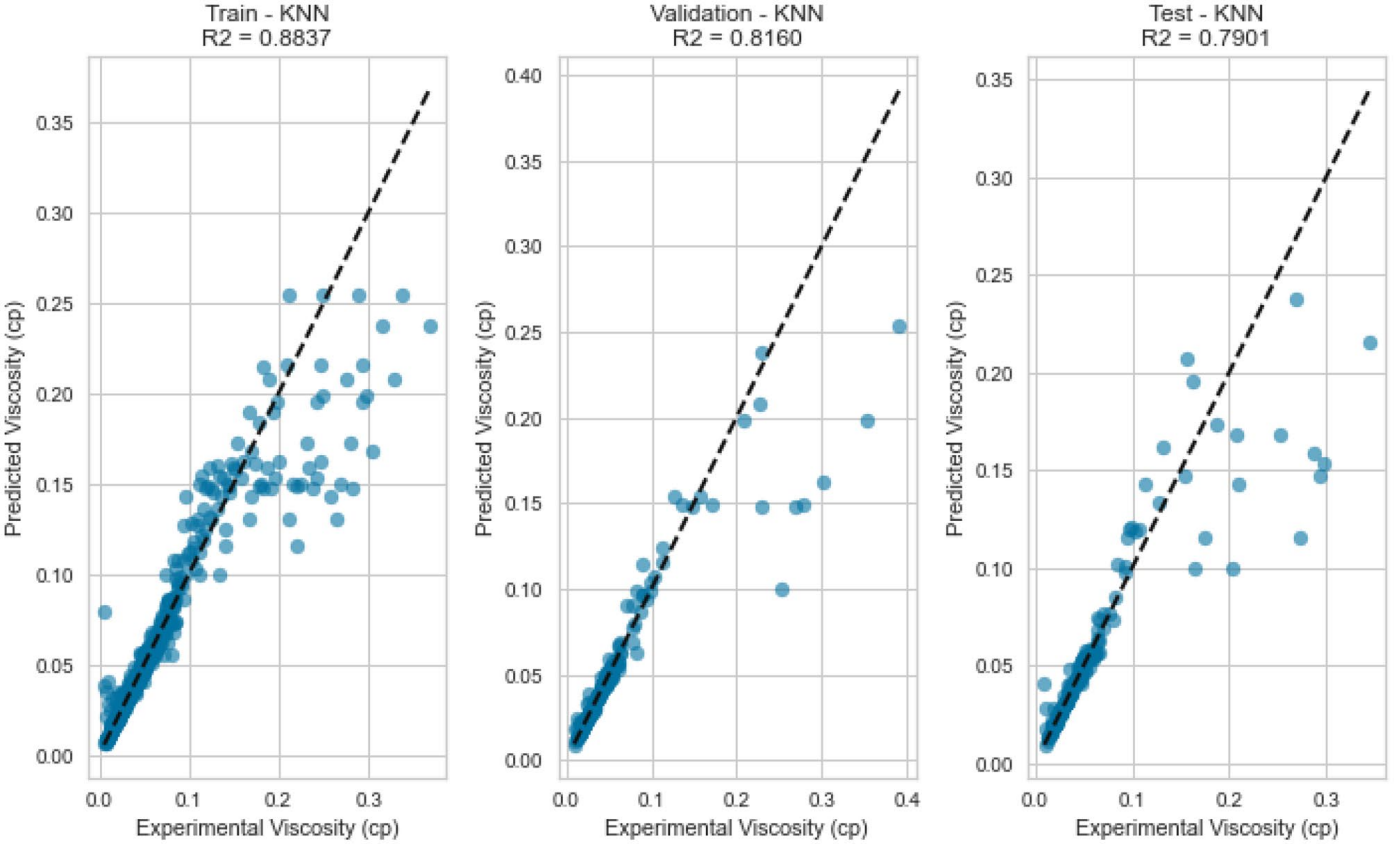
Cross plots of actual versus predicted values for Nitrogen viscosity (KNN model).

**Figure 40:**
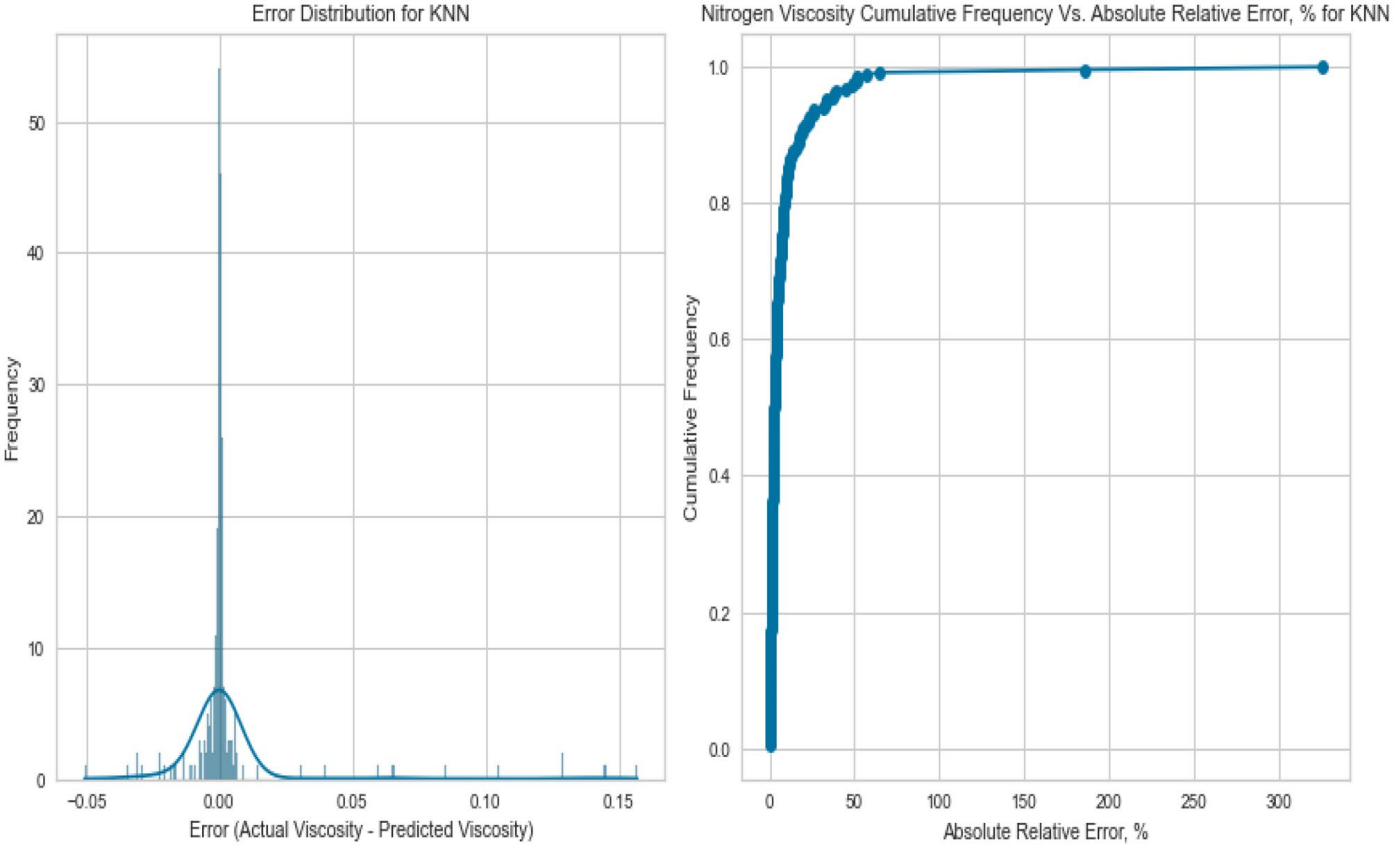
Error distribution for KNN model (nitrogen case).

**Figure 41:**
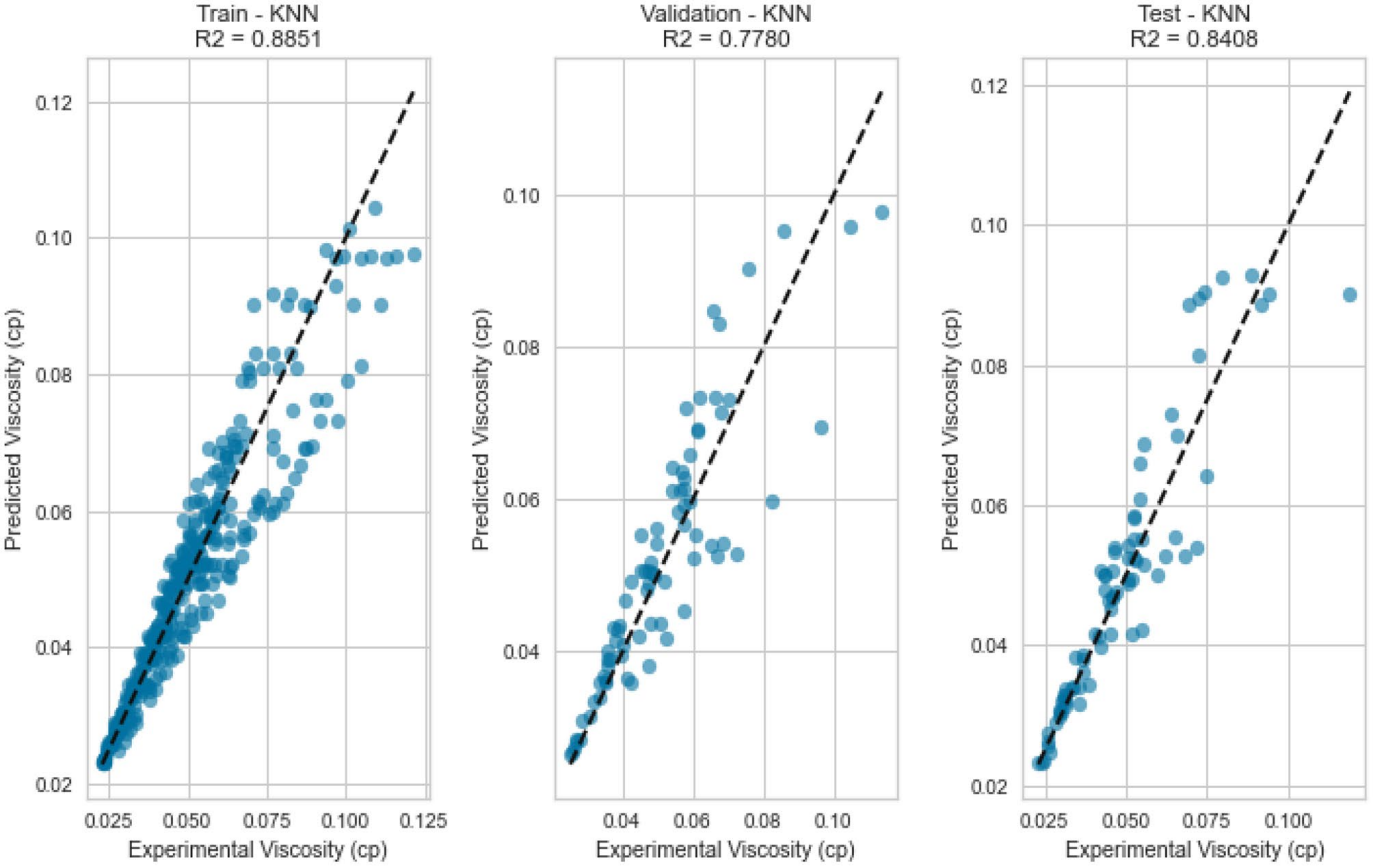
Cross plots of actual versus predicted values for natural gas mixtures viscosity (KNN model).

**Figure 42:**
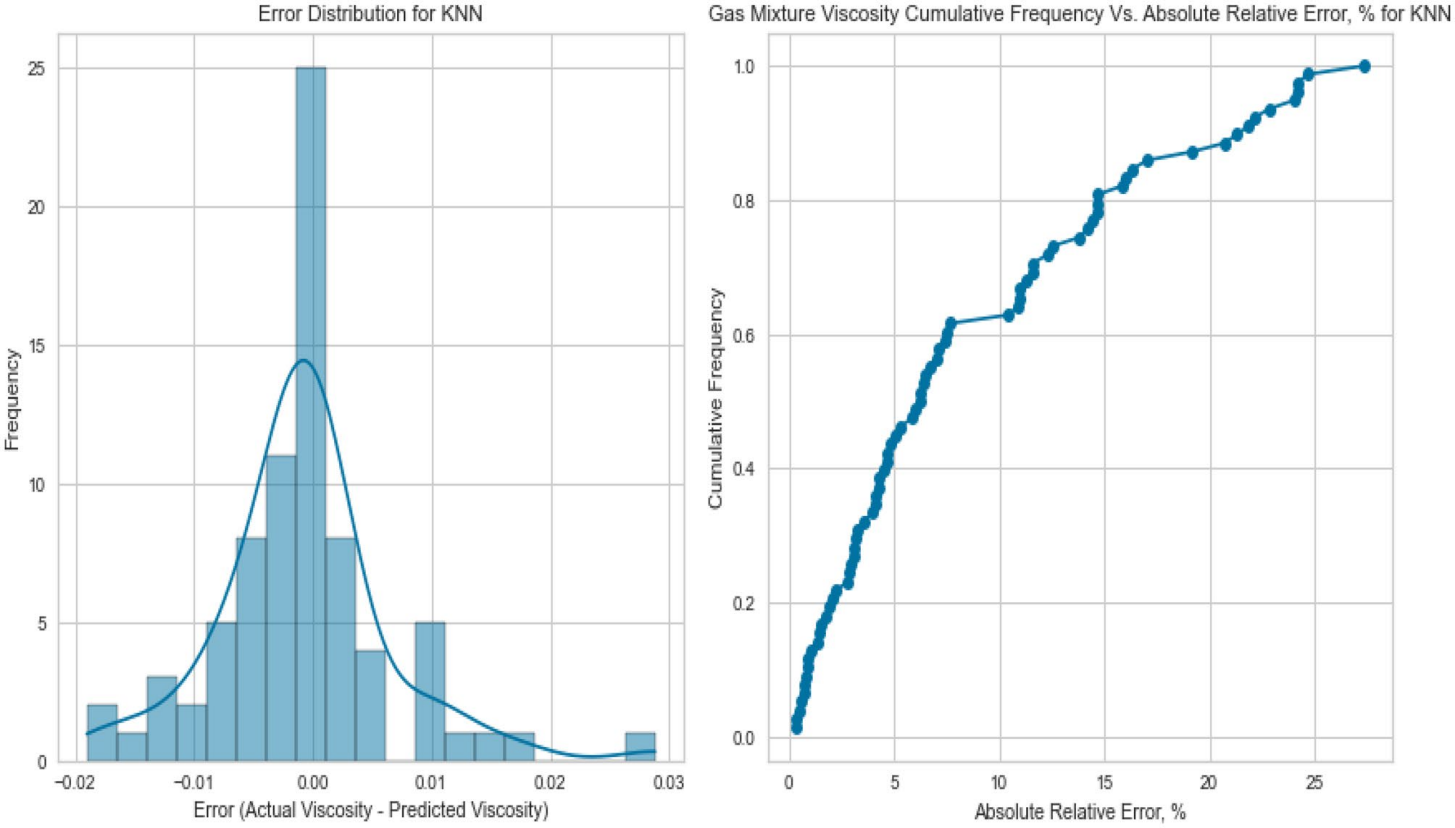
Error distribution for KNN model (natural gas mixtures case).

K-Nearest Neighbours leverages the proximity of data points to make predictions. Despite its simplicity, KNN yielded competitive results with an R^2^ score of 0.9837 for methane on the testing set. However, its performance fell slightly below that of more sophisticated models, potentially due to the inability to capture intricate nonlinear relationships. This is evident in its R^2^ scores of 0.8408 and 0.7901 for natural gas mixtures and nitrogen, respectively as displayed in Figs. 37, 38, 39, 40, 41, and 42. These findings underscore KNN’s proficiency in various scenarios, but its performance may vary based on the complexity of the dataset.

Considering the outcomes of our extensive evaluation using Coefficient of Determination (R^2^), Mean Squared Error (MSE), and Mean Absolute Error (MAE) reported in Table 2, the ensemble techniques, Random Forest and Gradient Boosting, emerged as the most promising models for accurately predicting gas viscosity across varying conditions. The ensemble nature of these methods contributed to their robustness and adaptability to the complex interactions present in the data. These findings underscore the significance of leveraging sophisticated machine learning approaches to enhance the precision of viscosity predictions, thereby facilitating the optimization of gas processing and reservoir management in the petroleum industry.

### Comparison of the performance of machine learning techniques (non-ANN models)

**Figure 43:**
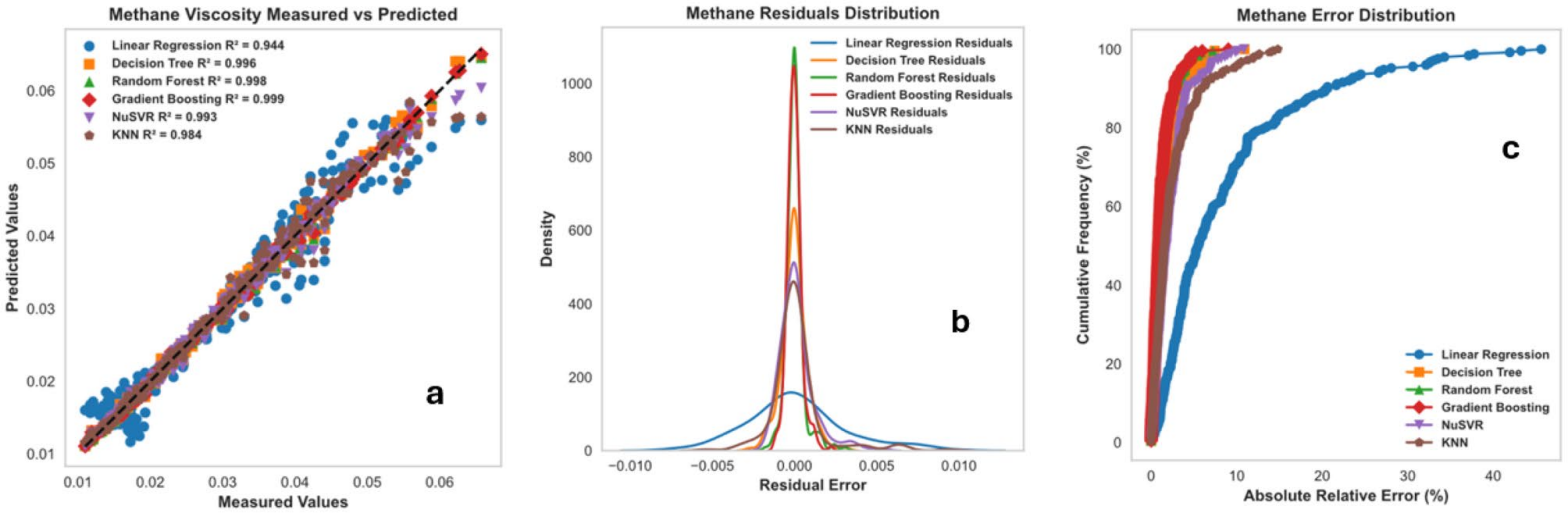
Prediction performance of the ML techniques (non-ANN) for viscosity prediction of methane: (**a**) cross plot, (**b**) residual error distribution, and (**c**) cumulative error frequency.

**Figure 44:**
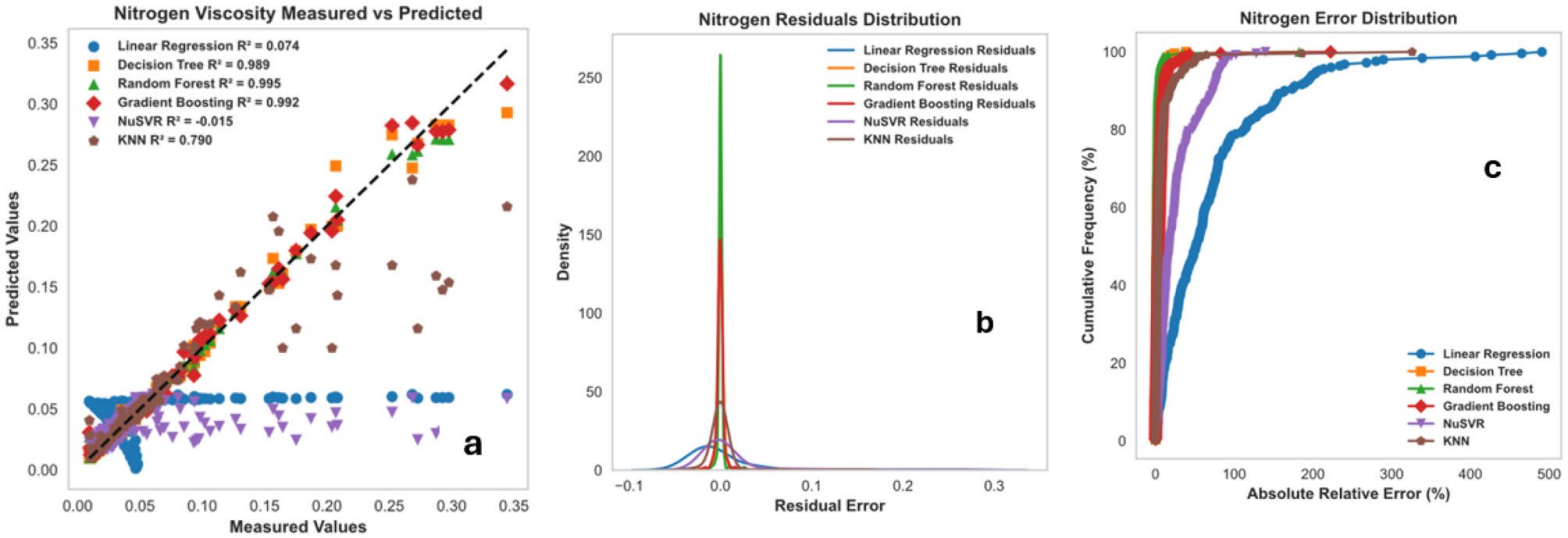
Prediction performance of the ML techniques (non-ANN) for viscosity prediction of Nitrogen: (**a**) cross plot, (**b**) residual error distribution, and (**c**) cumulative error frequency.

**Figure 45:**
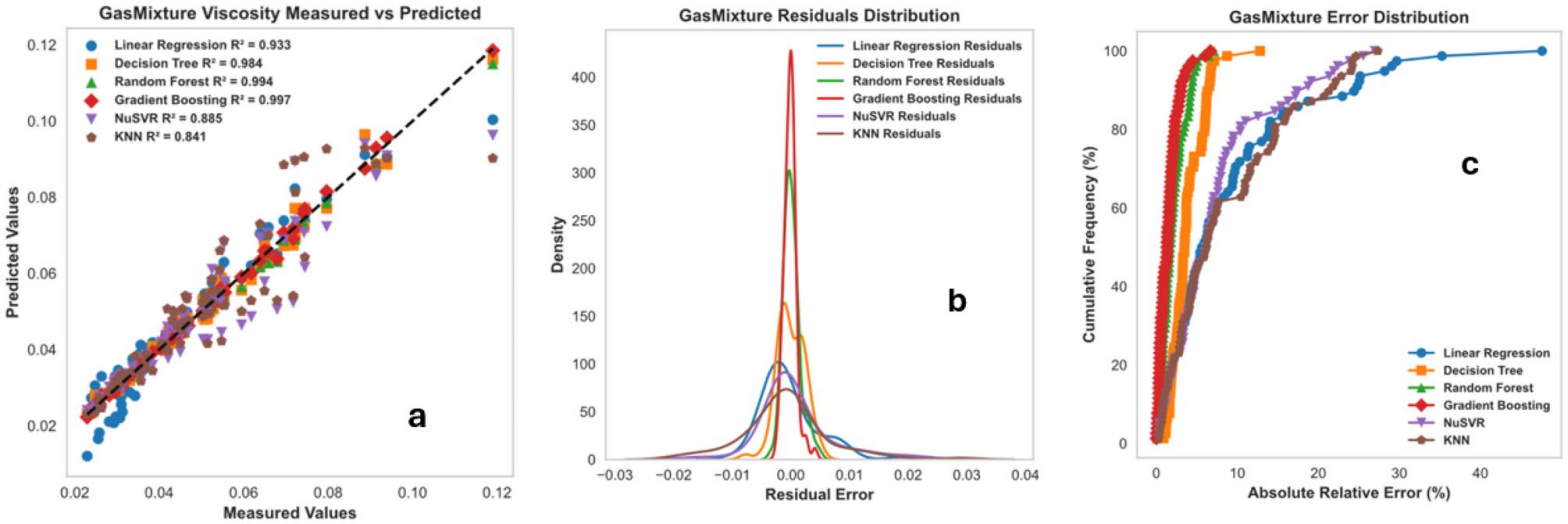
Prediction performance of the ML techniques (non-ANN) for viscosity prediction of Gas mixture: (**a**) cross plot, (**b**) residual error distribution, and (**c**) cumulative error frequency.

In this section, the performance of the RF, DT, Gradient Boosting, KNN, NuSVR, and linear regression were compared. Table 6 lists a summary of calculability efforts (such as CPU time and memories). It is shown that the proposed ML saves time. Moreover, Table 7 shows the tuned hyperparameters of each method in terms of the computational process of the developed model. The compared performance of RF, DT, Gradient Boosting, (KNN), and (NuSVR) was stated through cross plots and error distribution curves. Figure 43a displays the testing cross plots of methane using the mentioned ML techniques with their corresponding R^2^ values. The error performance is shown in Fig. 43b,c. The performance of Gradient Boosting and Random Forest was excellent during the testing process of the model. The Gradient Boosting and Random Forest methods resulted in R^2^ values of 0.993, and 0.991 during testing phases, respectively. The Decision Tree and NuSVR also resulted in superior performance with R^2^ values of 0.986 and 0.987 during testing. The KNN and Linear Regression resulted in good prediction, but they had the lowest performance with R^2^ values of 0.945 and 0.943 during the testing process. Similarly, Fig. 44a displays the testing cross plots of nitrogen using the mentioned ML techniques with their corresponding R^2^ values. The error performance was presented in Fig. 44b,c. The performance of Random Forest, Gradient Boosting, and Decision Tree was excellent during the testing process of the model. Moreover, the Random Forest, Gradient Boosting, and Decision tree resulted in R^2^ values of 0.995, 0.991, and 0.989 during the testing phases, respectively. The KNN resulted in intermediate performance with R^2^ values of 0.79 during testing. The NuSVR and Linear Regression resulted in very poor prediction with R^2^ values of 0.0146 and 0.0736 during the testing process. Likewise, the performance of the gas mixture was compared using the mentioned ML techniques. Figure 45a displays the testing cross plots of the gas mixture using the mentioned ML techniques with their corresponding R^2^ values. The error performance was presented in Fig. 45b,c for the prediction of the gas mixture viscosity. The performance of Random Forest, Gradient Boosting, and Decision Tree were excellent during the testing process of the model. The Random Forest, Gradient Boosting and Decision tree resulted in R^2^ values of 0.994, 0.996, and 0.984 during the testing phases, respectively. The KNN resulted in fair performance with R^2^ values of 0.79 during testing. The Linear Regression, NuSVR, and KNN resulted in the lowest performance with R^2^ values of 0.933, 0.885, and 0.84 during the testing process.

**Table 6:** Summary of run time and memory usage for machine learning models used in this study.

ML	Run time (s)			Memory usage, (MB)		
	Methane viscosity	Nitrogen viscosity	Gas mixture viscosity	Methane viscosity	Nitrogen viscosity	Gas mixture viscosity
Decision trees (DT)	2.03	2.02	2.02	0.098	0.088	0.004
Random forest (RF)	2.36	2.52	2.51	0.367	0.238	0.027
K-nearest neighbors (KNN)	2.02	2.02	2.02	0.004	0.003	0.01
XGBoost (extreme gradient boosting)	2.11	2.17	2.15	0.008	0.006	0.02
NuSVR	2.28	2.26	2.18	0.242	0.089	0.0625

**Table 7:** Optimized hyperparameters for machine learning models used in this study.

Techniques	Hyperparameters	Range	Optimized values for methane viscosity	Optimized values for nitrogen viscosity	Optimized values for gas mixture viscosity
Decision trees (DT)	Maximum depth	5–25	15	22	11
	Minimum samples split	2–10	2	2	2
	Minimum samples leaf	1–5	1	1	1
Random forest (RF)	Number of trees	10–150	100	100	100
	Maximum depth	None, 5, 10	None	None	None
	Samples split minimum	2–10	2	2	2
	Samples leaf minimum	1–5	1	1	1
	Number of Features	2, 3	2	2	3
K-nearest neighbors (KNN)	Number of Neighbors	5–20	5	5	5
	Distance metric	Minkowski, Euclidean, Manhattan	Minkowski	Minkowski	Minkowski
XGBoost (extreme gradient boosting)	Number of trees	10–150	100	100	100
	Maximum depth	None,5, 10	3	3	3
	Learning rate	0.01–1.0	0.1	0.1	0.1
	Subsample	0.5–1.0	1.0	1.0	1.0
	Column sample by tree	0.5–1.0	1.0	1.0	1.0
NuSVR	Regularization parameter; C	0.01–10	1.0	1.0	1.0
	Kernel	Linear, poly, rbf	rbf	rbf	rbf
	Gamma	Scale, auto	Scale	Scale	Scale

## Development of ANN models

**Figure 46:**
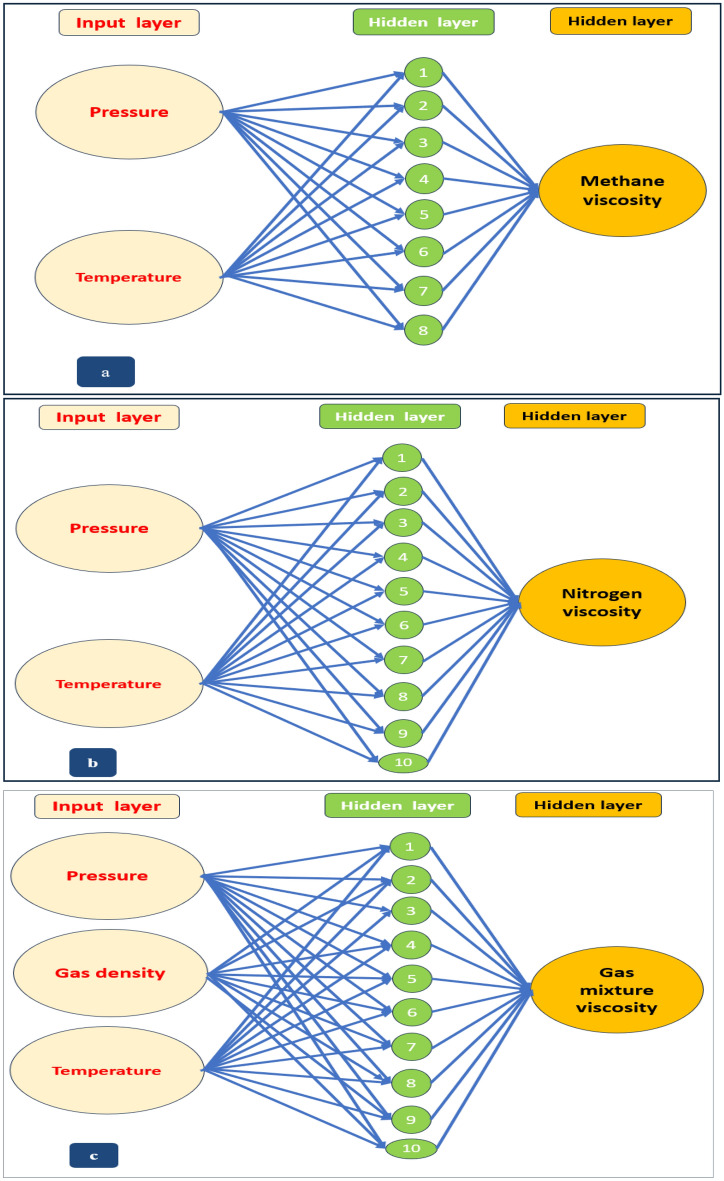
Three-layered ANN model consists of (input, hidden, and output layers) for prediction viscosity of (**a**) methane, (**b**) nitrogen, (**c**) gas mixture.

An artificial neural network is a brain-like system that performs computational models on numeric inputs which are considered interconnected nodes (neurons) and generates a single complex functional output relevant to the inputs^2,31^. ANN is considered an artificial intelligence technique used in numerous science and technology fields that comprises learning, storing, and information recalling depending on the training dataset^11^. Intelligent algorithms such as (ANN), (PN), (GA), (GRN), and (GMDH)^2^ ease the complexity of computational calculations by altering complex formulations to simple ones^3^. One type of neural network is the (MLF), in which neurons of hidden layers are determined, while the (MFFNN) is the most used neural network for function approximation systems^9^. A three-layered MFFNN encompasses input, hidden, and one output layer displayed in Fig. 46 used to approximate complex functions^37^. The characteristics of the ANN model for modeling the viscosities of CH_4_, N_2_, and natural gas mixture, as well as the number of training, validation, and testing datasets for each model, are summarized in Table 8. To select the optimum model that represents the collected data, many trials have been done by changing the transfer function (Logistic-sigmoid/Tan-sigmoid), several neurons of the hidden layer (5, 6, 7, 8, 9, 10), and several hidden layers using the Levenberg–Marquardt technique as a training algorithm as discussed in the following sections. LM algorithms are one of the most popular MLPNN modeling which uses the nonlinear least-squares techniques^1^.

**Table 8:** The ANN model’s characteristics of CH_4_, N_2_, and natural gas mixture viscosities.

Parameter	Description		
	Methane	Nitrogen	Natural gas mixture
Learning algorithm	Levenberg–Marquardt		
The hidden layer’s activation function	Logistic-Sigmoid		
The output layer’s activation function	Pure-linear		
layers number	3	3	3
Number of neurons in the 1st layer	2	2	3
Number of neurons in the hidden layers	8	10	10
No. of dataset	1664	1672	968
No. of training dataset	1166 (70%)	1172 (70%)	678 (70%)
No. of validation dataset	249 (15%)	250 (15%)	145 (15%)
No. of testing dataset	249 (15%)	250 (15%)	145 (15%)

### Development of ANN model for calculating methane viscosity

A logistic sigmoid with eight neurons in the hidden layer is the optimum transfer function. The selected optimum model predicts the methane viscosity with high precision. This model achieves R^2^ = 0.99918, a standard deviation of 1.47, RMSE of 0.00038, MPE of − 0.01%, and MAPE of 1.10% as presented in Table 9. The complete description of the model as described in Table 8 indicates that the proposed model consists of 3 layers. The 1st layer has two neurons for the inputs (pressure and temperature), the hidden layer has 8-neurons, and the third layer has one neuron for the output (methane viscosity).

**Table 9:** Statistics for selecting the optimum ANN model of methane viscosity.

	5	6	7	8	9	10	8 T
R^2^	0.99888	0.99911	0.99911	0.99918	0.99916	0.99918	0.999159
SD	1.80446	1.54488	1.52523	1.47002	1.59462	1.53941	1.526387
RMSE	0.00044	0.0004	0.0004	0.00038	0.00038	0.00038	0.000385
RE	− 0.0253	− 0.0307	− 0.0304	− 0.0103	− 0.0213	− 0.0178	− 0.09632
AE	1.35063	1.15279	1.16752	1.10267	1.16623	1.13364	1.128152

The proposed ANN model for methane viscosity calculation can be illustrated as follows:

For i = 1 to none of the neurons and for j = 1 to none of the inputs, the hidden layer inputs are computed using the following formula:

9 $$S_{i,j} = \sum\limits_{j = 1}^{N} {(w_{i,j} x_{j} } ) + b_{j}$$

where $\text{represents}$ the normalized pressure and temperature respectively which can be formulated as:

10 $$P_{n} = 0.00008P - 1.001176$$

11 $$T_{n} = 0.004938T - 1.3802475$$

The viscosity of methane can be formulated as follows:

12 $$\mu = 0.055806\left[ {\sum\limits_{i = 1}^{N} {\left( {\frac{{w_{hoi} }}{{1 + \exp \left( { - S_{i} } \right)}}} \right) + b_{ho} } } \right] + 0.065545$$

The model weights and bias coefficients between the layers are explained in Table 10.

**Table 10:** Model coefficients between the layers for methane viscosity modeling.

Neuron #	${w}_{i,j=1}$	${w}_{i,j=2}$	${b}_{i}$	${w}_{hoi}$	${b}_{ho}$
1	− 0.9263	− 1.155	4.6572	− 4.4534	− 3.8254
2	3.7563	5.3226	− 6.128	− 0.24667	
3	0.6472	− 3.359	− 4.386	3.7625	
4	0.7993	− 0.249	0.8579	5.1395	
5	3.6784	− 0.05	2.651	4.7614	
6	− 4.4112	− 0.441	− 2.611	1.3221	
7	− 0.2755	3.3276	4.9759	4.6478	
8	2.716	− 0.426	2.4924	− 5.1641	

### Development of ANN model for calculating nitrogen viscosity

The optimum model includes two hidden layers with ten neurons for each and the transfer function is Logistic sigmoid. The selected optimum model predicts the nitrogen viscosity with high accuracy. This model achieves R^2^ = 0.997, a standard deviation of 35.05, RMSE of 0.0025, MPE of − 1.08%, and MAPE of 3.22% as presented in Table 11. The complete description of the model as presented in Table 8 includes that the proposed model consists of four layers. The first layer has two neurons for the inputs (pressure and temperature), two hidden layers have ten neurons for each, and the third layer has one neuron for the output (nitrogen viscosity).

**Table 11:** Statistics for selecting the optimum ANN model of nitrogen viscosity.

	5	6	7	8	9	10	10 T	10SSP
R^2^	0.98631	0.98553	0.98961	0.98477	0.98255	0.99002	0.988776	0.997282
SD	68.4435	80.9865	61.1549	75.99428	88.7061	56.3272	57.67505	35.05219
RMSE	0.00559	0.00577	0.00487	0.005904	0.0063	0.00478	0.005082	0.002506
RE	− 4.5925	− 5.5482	− 4.0407	− 7.01165	− 8.0375	− 3.1012	− 3.93047	− 1.08431
AE	10.4979	10.323	8.54235	11.71631	12.0903	8.14484	8.857575	3.221568

The proposed ANN model for calculating the nitrogen viscosity can be illustrated as develops. The inputs for the first hidden layer are calculated, where the normalized pressure and temperature can be expressed as:

13 $$P_{n} = 0.000081632P - 1.001184$$

14 $$T_{n} = 0.0008997T - 0.691408$$

The outputs from the first hidden layer are calculated using the Logistic-sigmoid function as follows:

15 $$H_{i} = \frac{1}{{1 + \exp \left( { - S_{i} } \right)}}$$

The input of the second hidden layer is calculated using the following expression:

16 $$SS_{i,j} = \sum\limits_{j = 1}^{N} {\left( {w_{i,j} H_{j} } \right)} + b_{j}$$

The outputs from the second hidden layer are computed as follows:

17 $$HH_{j} = \frac{1}{{1 + \exp \left( { - SS_{j} } \right)}}$$

The nitrogen viscosity can be formulated as:

18 $$\mu_{N2} = 0.19294\left[ {\sum\limits_{i = 1}^{N} {\left( {HH_{j} w_{hoij} } \right) + b_{ho} } } \right] + 0.19846$$

The ANN model weights and biases between the layers are explained in Tables 12, 13, and 14.

**Table 12:** Model coefficients between the layers between the input and the first hidden layer.

Neuron #	${w}_{i,j=1}$	${w}_{i,j=2}$	${b}_{i}$
1	3.9617	− 9.531	− 6.425
2	4.9625	6.1014	− 7.94
3	− 10.945	29.632	17.707
4	5.0953	− 4.108	− 0.222
5	0.5023	1.5331	− 0.233
6	− 0.0981	− 8.291	− 6.497
7	− 5.064	− 0.697	− 5.634
8	0.8509	20.827	20.503
9	17.824	− 0.04	17.895
10	21.781	11.666	33.688

**Table 13:** Model coefficients between the first and the second hidden layers.

W1	W2	W3	W4	W5	W6	W7	W8	W9	W10	b
− 0.88253	1.8382	− 1.3036	− 2.5301	2.1458	6.7651	3.3736	− 4.2357	− 10.9149	1.2957	1.1044
− 4.519	− 3.3167	− 1.8767	0.77471	0.078324	− 4.3585	1.3972	− 0.94983	5.3246	− 3.1295	6.4301
3.2164	− 2.2709	21.2044	0.34493	− 1.7776	4.5619	1.8436	− 19.9233	6.6582	− 12.3119	− 5.9605
2.3312	− 3.2796	5.6372	− 3.5157	1.6309	− 6.8675	− 1.6736	7.0359	1.9375	11.7241	− 7.7165
2.7793	− 2.3727	− 1.5271	− 1.9545	1.6769	3.3274	3.5357	5.4488	− 3.8658	− 7.6838	− 0.23225
2.2131	2.2295	0.091128	0.51019	3.8452	− 1.5995	− 1.552	− 3.3308	0.26275	2.381	− 3.0239
2.6254	1.8697	− 1.8305	3.4868	0.33837	− 3.4281	2.455	− 0.29006	− 3.2851	− 0.58462	− 3.3005
− 1.6344	− 1.7562	5.1133	1.2104	− 3.1481	− 7.2707	− 0.47054	7.166	0.44856	10.604	− 5.4499
4.0064	2.6696	1.4004	− 0.29366	− 0.59161	3.5769	− 0.12439	− 1.8023	1.5135	− 14.208	6.3726
− 3.0234	− 4.2309	0.43802	− 0.68803	− 3.3328	0.76091	0.5076	− 1.3548	− 0.11573	− 1.4784	0.527

**Table 14:** Model coefficients between the second hidden layer and the output layer.

Neuron #	${w}_{hoi}$	${b}_{ho}$
1	− 10.62	− 3.811
2	3.2753	
3	− 9.093	
4	5.5889	
5	6.8493	
6	0.1016	
7	− 0.312	
8	− 5.855	
9	8.9775	
10	− 1.613	

### Development of ANN model for calculating gas mixture viscosity

The optimum model includes one hidden layer with ten neurons besides the input and output layers, and the transfer function is Logistic Sigmoid. The selected optimum model predicts the gas mixture viscosity with high accuracy. This model achieves R^2^ = 0.9998, a standard deviation of 1.75, RMSE of 0.00029, MPE of − 0.03%, and MAPE of 1.26. The complete description of the model as presented in Table 8 includes that the proposed model consists of three layers. The first layer has three neurons for the inputs (pressure, temperature, and gas density), one hidden layer has ten neurons, and the third layer has one neuron for the output (gas mixture viscosity). The proposed ANN model for calculating the gas mixture viscosity can be designated as follows. The inputs for the first hidden layer are calculated using Eq. (9). The normalized pressure, temperature, and gas density can be expressed as:

19 $$P_{n} = 0.00008P - 1.001176$$

20 $$T_{n} = 0.005692T - 1.048947$$

21 $$\rho_{n} = 0.166473\rho - 1.000385$$

The viscosity of the gas mixture can be calculated by the following function:

22 $$\mu = 0.055806\left[ {\sum\limits_{i = 1}^{N} {\left( {\frac{{w_{hoi} }}{{1 + \exp \left( { - S_{i} } \right)}}} \right) + b_{ho} } } \right] + 0.065545$$

The weights and biases between the layers are explained in Tables 15 and 16.

**Table 15:** Weights and biases between the input layer and the hidden layer.

Neuron #	${w}_{i,j=1}$	${w}_{i,j=2}$	${w}_{i,j=3}$	${b}_{i}$
1	− 3.8771	1.1535	− 11.29	− 11.28
2	− 0.132	− 7.485	1.7831	5.9847
3	− 0.2822	2.7924	− 10.58	13.997
4	− 0.0243	1.2417	0.9334	0.0813
5	− 0.9264	− 1.502	− 12.4	9.9113
6	0.3968	1.4546	5.1288	− 4.259
7	− 0.4419	− 1.818	− 0.841	− 1.45
8	6.9001	− 1.391	− 6.572	− 3.977
	− 1.2697	0.8053	8.8788	− 9.807
	− 0.2064	− 7.649	0.0859	7.5045

**Table 16:** Weights and biases between the second hidden layer and the output layer.

Neuron #	${w}_{hoi}$	${b}_{ho}$
1	− 0.908	5.3173
2	1.8097	
3	− 2.083	
4	− 9.73	
5	1.7001	
6	6.0999	
7	− 4.743	
8	− 1.803	
9	− 1.122	
10	− 1.722	

**Figure 47:**
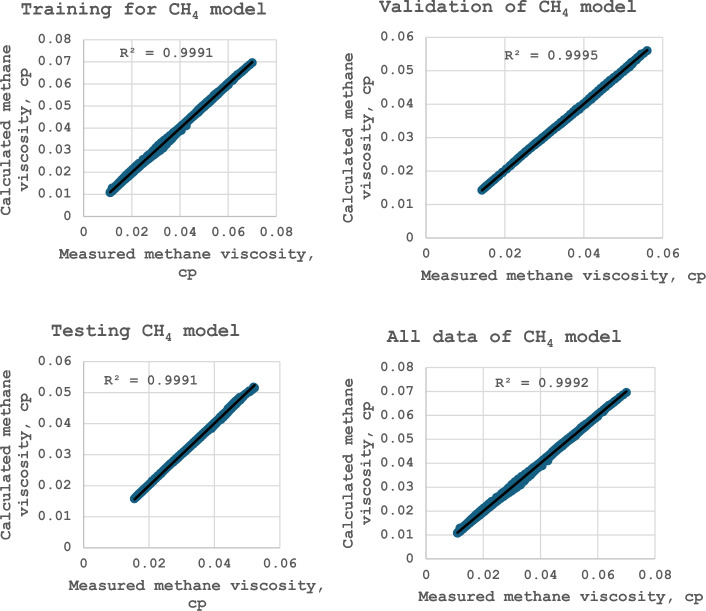
Cross plots of methane viscosity model (after this work).

**Figure 48:**
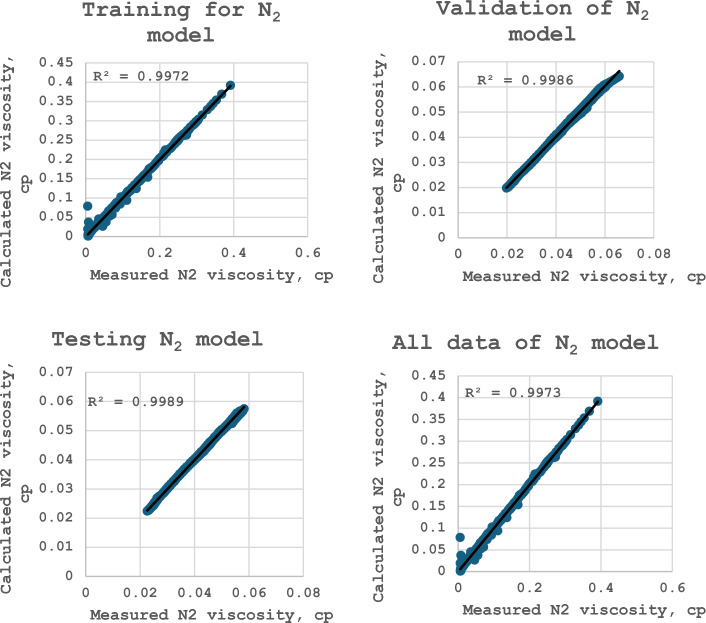
Cross plots of nitrogen viscosity model (after this work).

**Figure 49:**
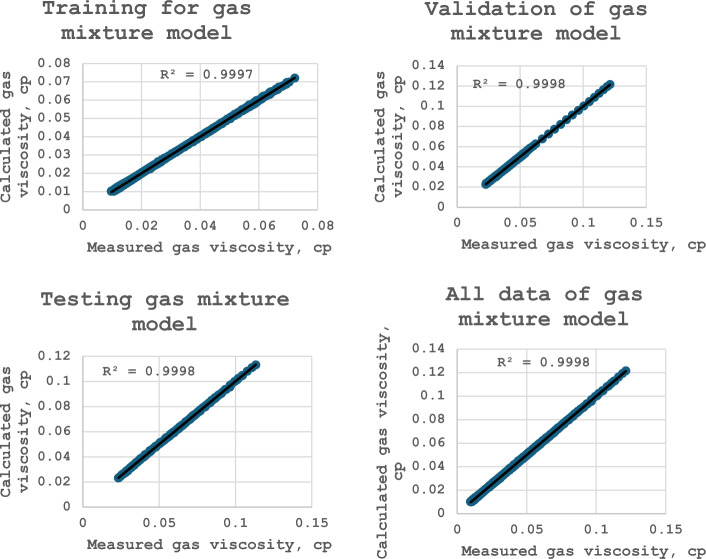
Cross plots of gas mixture viscosity model (after this work).

Figures 47, 48, and 49 show the outputs regression plots for training, validation, testing, and all datasets for the methane, nitrogen, and gas mixture viscosity model respectively. When the ANN outcomes match the targets, the data points should be close to the line of the unit slope for an optimal fit. In this work, the estimated values versus the experimental one fit well closely around the 45° lines for all data sets, including training, validation, and testing data which implies the strength of the proposed model^1^. The coefficient of determination values is more than 0.999, which demonstrates an outstanding arrangement between the predicted and measured values for the three proposed models^37^.

**Figure 50:**
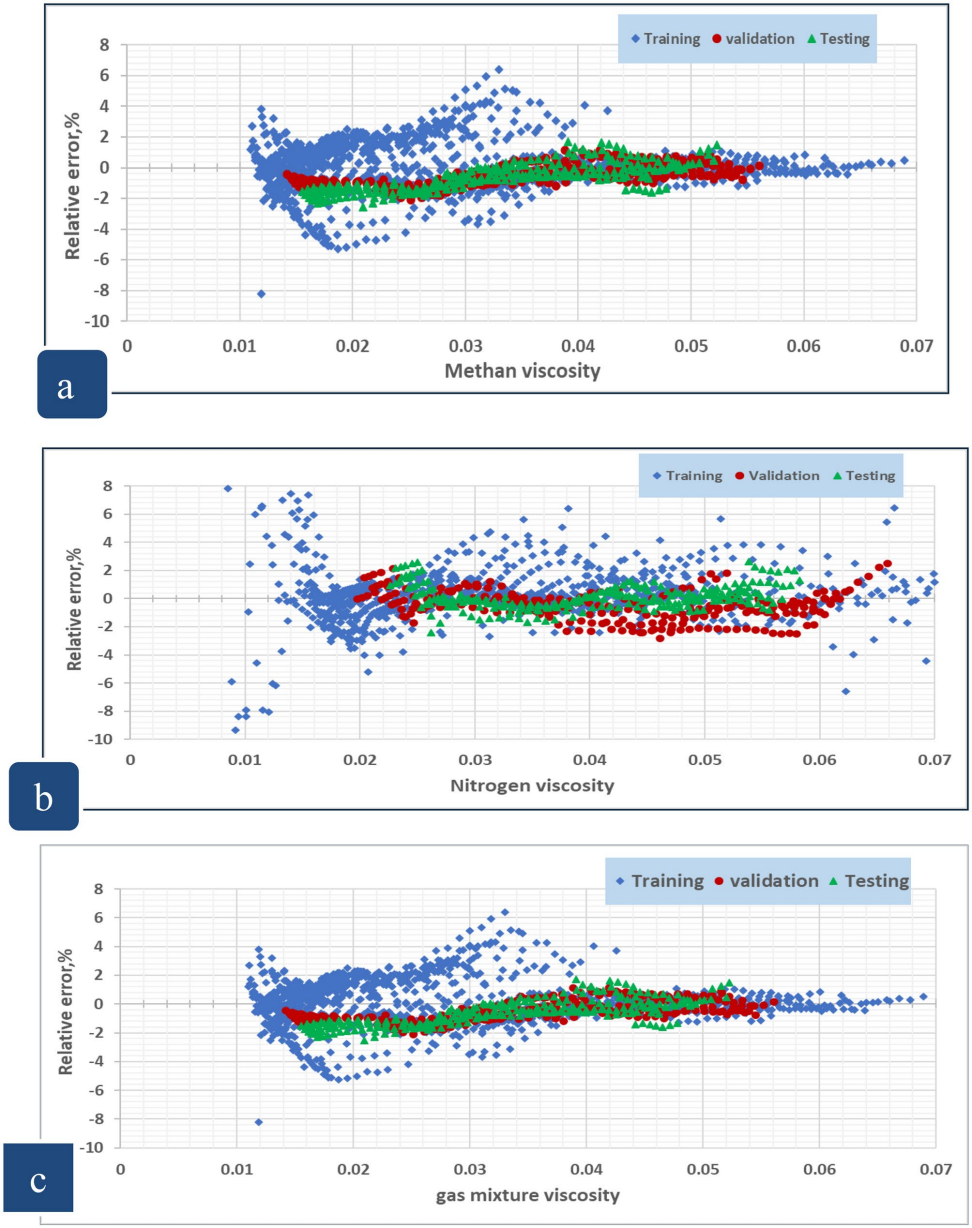
Relative error distribution for (**a**) methane viscosity predicted by ANN model, (**b**) nitrogen viscosity predicted by ANN model, (**c**) gas mixture viscosity predicted by ANN model.

**Figure 51:**
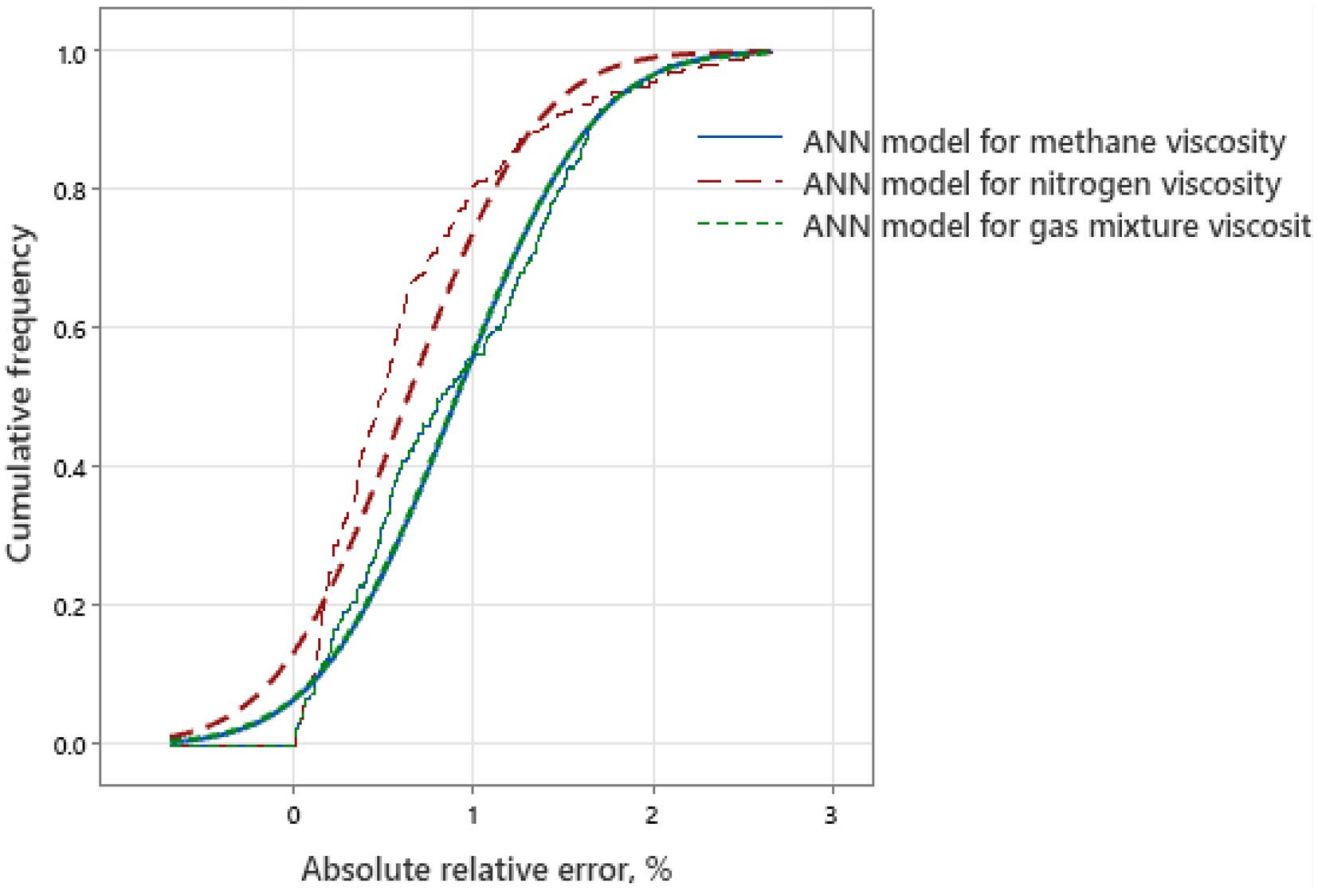
Cumulative frequency versus ARE% for the developed ANN models.

Furthermore, Fig. 50a–c shows the relative errors between the developed model’s outputs and the consistent experimental values for the training, validation, and testing sets. Since there is no discernible error trend in the predictions, error distribution curves show that the suggested models are capable of accurately estimating natural gas viscosity over a broad range of P, and T conditions^3^. The accuracy of the developed model is indicated by a denser cloud of data points surrounding the zero-error line. Additionally, at high P, and T conditions, these curves show that the suggested model does not exhibit a significant error trend^3^. Furthermore, Fig. 51 illustrates the cumulative frequency of the ARE related to the developed ANN models. According to the figure, more than 90% of the estimated viscosity values for methane, nitrogen, and gas mixture by the ANN model had an absolute relative error of less than 2%. Hence, these results demonstrate that ANN models had acceptable performance and could successfully estimate the gas viscosity for CH_4_, N_2_, and natural gas mixture.

## The potential advantages and limitations of the present study

The present study potentially presents faster and more effective techniques for predicting the viscosities of methane, nitrogen, and natural gas mixtures under normal and harsh conditions, compared to traditional experimental approaches. The developed ML models can certainly be employed for new data, granting the potential to predict viscosities for various mixtures and conditions without the need for further experimental measurements. The established ML models in the present study can also serve as a foundation for further improvement of the predictive models. As more data becomes available, their accuracy and reliability can be enhanced. It is important to add that the proposed ML algorithms can be considered as a substitution of the experimental measurements of PVT properties if the experimental parameters are within the considered ranges. From a limitation point of view, the developed Machine learning models would be better at interpolating within the range of the training data rather than extrapolating beyond it. Therefore, the accuracy of the predictions may decrease when applied to conditions significantly different from those present in the training data.

## Conclusion

Mathematical models based on machine learning models were used to predict the viscosities of methane, nitrogen, and gas mixtures at high temperatures and high-pressure conditions. Moreover, for effective gas viscosity prediction, several optimization tools including statistical and ML approaches were used. Afterward, several verification principles were implemented to evaluate the developed models. The ML models suggested in this research hold significant importance for researchers and industry specialists involved in natural gas simulation, processing, and recovery. In general, the following conclusion can be drawn:

- The proposed ML models in this work effectively addressed the predicting gas viscosity under high-temperature and high-pressure circumstances.
- A database consisting of 4304 sets of experimental data was collected from the literature before the development of the ML models.
- Three novel correlations have also been developed for CH_4_, N_2_, and natural gas viscosities using ANN. The measured and ANN-estimated viscosity values showed excellent agreement with R^2^ = 0.99 for testing data sets.
- The performances of the ANN, RF, and Gradient boosting were outstanding prediction modeling during the training and testing phases of CH_4_, N_2_ gas, and gas mixture viscosities.
- The ANN modeling resulted in R^2^ values of 0.99 and 0.99 for the training and testing of the CH^4^, N^2^ gas, and gas mixture models, respectively.
- Linear regression, NuSVR, and KNN were the least performance ML models for predicting CH_4_, N_2_ gas, and gas mixture viscosity models, whereas Linear regression and NuSVR models were unable to predict N_2_ gas viscosity.
- The inclusive efficacy of the ML models, ordered from high ranking to lowest ranking, was ANN > Gradient Boosting > RF > DT > KNN > NuSVR > Linear Regression, suggesting that ANN, Gradient Boosting, RF, and DT perform better than KNN, NuSVR, and Linear Regression.
- The study outcomes indicate that data-based ML could be a beneficial model for precisely predicting the viscosity of CH_4_, N_2_ gas, and gas mixture under normal and hard conditions, gaining enhancement in the modeling of natural gas operations.

## Data Availability

The datasets used and/or analysed during the current study are available from the corresponding author on reasonable request.

## Abbreviations

Pr: Reduced pressure
Tr: Reduced temperature
Ppr: Pseudo-reduced pressure
Tpr: Pseudo-reduced temperature
Pc: Critical pressure
Tc: Critical temperature
AARE%: Average absolute relative error percent
ARE%: The average relative error percent
GMDH: Group method of data handling
ANFIS: Adaptive neuro-fuzzy inference system
SVM: Support vector machine
ANN: Artificial neural network
GA: Genetic algorithm
Pres: Reservoir pressure, psi
Tres: Reservoir temperature, °F
MLF: Multi-layer feed forward network
GRN: Generalized regression neural nets
PN: Probabilistic neural nets
MFFNN: Multilayer feed-forward neural network
P_n_ and P: The normalized and actual parameters, respectively
P_max_ and P_min_: The maximum and minimum values of P, respectively
RMSE: Root means square error
SD: Standard deviation
R^2^: Coefficient of determination
